# Supplementary material for: Factors that influence the feasibility and implementation of a complex intervention to improve the treatment of peripheral arterial disease in primary and secondary care: a qualitative exploration of patient and provider perspectives
Source: BMJ Open. 2023 Jan 23;13(1):e066883. doi: 10.1136/bmjopen-2022-066883 (PMC9872459; doi:10.1136/bmjopen-2022-066883)
Supplement: Supplementary data [file bmjopen-2022-066883supp003.pdf]

Full coding framework

Theme 1: The potential to make a difference

1.1 An opportunity to meet patient need

1.1.1 Improves care and outcomes
1.1.1.1 Standardises a minimum quality of care
1.1.2 Improves awareness and understanding of PAD
1.1.3 Improves awareness of existing NICE guidance
1.1.3.1 Provides a tool to support implementation of NICE guidance
1.1.3.1.1 The guidance is clear but we don't always follow it
1.1.4 Builds your relationship with the patient
1.1.4.1 Empowers patients by being informed

1.2 Shared primary and secondary care working

1.2.1 Working as a part of a whole team
1.2.1.1 Allows for secondary care oversight
1.2.1.2 An opportunity for collaborative working
1.2.1.2.1 Managing expectations and understanding each other's roles
1.2.1.2.2 We don't sing off the same hymn sheet
1.2.1.2.2.1 The patient is in the middle
1.2.1.3 Multidisciplinary working
1.2.1.3.1 Enabling specialist care from different people
1.2.1.4 Promoting continuity of care
1.2.1.4.1 There's lots of different clinics and different people involved in your care
1.2.1.4.2 Timely access back into secondary care if needed
1.2.2 Joining up and building relationships across the team
1.2.2.1 We don't ever see secondary care specialists
1.2.2.2 Access to the vascular team (or not)
1.2.2.2.1 A direct contact number for the vascular team
1.2.2.3 We've done our bit and now it's over to the GP
1.2.3 Clear open communication across community and hospital services
1.2.4 Working together and learning together
1.2.4.1 An opportunity for two-way learning
1.2.4.1.1 Secondary care can learn from this too
1.2.4.1.1.1 Support for screening questions on prescribing and contraindications
1.2.4.1.1.1.1 Holistic picture of medication profile, side effects, competing medication
1.2.4.1.1.2 Familiarity (or not) with reviewing cholesterol and HbA1c's – it's a GP thing

Theme 2: A solution for addressing the gap in no man's land

2.1 We need to target patients earlier

2.1.1 Patients presenting too late
2.1.1.1 Delayed referral to secondary care
2.1.1.1.1 We can't make a referral anyway
2.1.1.1.1.1 Secondary care referral thresholds are too high
2.1.1.1.1.1.1 Patients don't meet the criteria so have to wait until it gets worse
2.1.1.2 Waiting for secondary care to make the diagnosis
2.1.1.2.1 Support general practice to do some of the diagnostics
2.1.1.2.1.1 Lack of access to testing equipment in the community
2.1.1.2.1.2 Secondary care are gatekeepers for ABPIs and duplexes

2.2 Primary care needs to do more sooner

2.2.1 Adapt for use in general practice as part of a PAD prevention programme

2.2.1.1 Would promote earlier conversations with patients
2.2.1.2 Useful for managing patient expectations
2.2.1.3 Would support primary care to start best medical therapy
2.2.1.4 Could be used to provide additional information for referral to vascular team
2.2.1.4.1 Would support triage in secondary care
2.2.2 Use as an ongoing conversation guide
2.2.2.1 Existing relationships with patients will help to reinforce the message
2.2.2.2 Informing patients reduces the chance of presenting late or in crisis

Theme 3: Prioritising and making it happen

3.1 Prioritising alongside the burden of work
3.1.1 It shouldn't be seen as an extra thing
3.1.1.1 Previous experience of using intervention/checklists
3.1.1.2 Checklists are a quick quality check
3.1.1.3 Checklists are a useful memory prompt
3.1.1.4 Checklists are a threat to autonomy
3.1.1.5 Checklists are tick-box exercises rather than safety mechanisms
3.1.2 Confidence to implement guidelines
3.1.2.1 It's essentially what I always do
3.1.3 It takes time to build the relationship and have the conversation
3.1.3.1 Time to talk requires extra staff
3.1.3.2 Time burden of dictating long clinic letters
3.1.3.3 Limited access to blood patient monitoring and cholesterol tests in out-patient clinics
3.1.4 Impact of staff shortages
3.1.4.1 Reduced staffing due to covid
3.1.4.2 Reduced district nurse staffing for conducting ABPIs
3.1.5 Primary care is not an infinite resource
3.1.5.1 Long waiting times to be seen or to get tests
3.1.5.1.1 Sometimes it can take weeks to get them to answer a query over the phone
3.1.5.2 Sixty calls and forty letters a day
3.1.5.3 Patients don't always see the same GP
3.1.5.4 Championing the cause or doing the bare minimum and going home on time
3.1.5.4.1 Using research to demonstrate a difference

3.2 Working across primary and secondary care systems
3.2.1 Preventative medicine and health promotion is core to primary care practice
3.2.1.1 Existing systems in place for primary care medication management and monitoring
3.2.1.1.1 It can take weeks to get information on the system
3.2.1.1.2 Time delay between writing and receiving clinic letters
3.2.1.1.3 It can take up to three months to receive hospital letters
3.2.1.1.3.1 Provide 28 days medication to allow for delay in seeing GP
3.2.1.1.3.2 Patient difficulty ordering repeat medication
3.2.1.1.4 Avoid the extra step and order the test in secondary care
3.2.1.1.5 Secondary care to initiate smoking cessation referral
3.2.1.1.5.1 Local variation in community set-up of smoking cessation, dietary advice, exercise therapy support
3.2.1.1.5.1.1 No formal set-up in primary care to pick it up
3.2.1.1.5.1.2 Link with national protocol for weight reduction
3.2.1.1.6 It's not commissioned or financially viable
3.2.2 Provide a copy of the clinic/GP letter to the patient to reinforce shared messaging
3.2.2.1 Patients to hand deliver letters to general practice
3.2.3 Repeat GP action letter is a duplicate, adds to the workload, and sets the wrong tone
3.2.3.1 It gives the impression that secondary care do not trust GPs

3.3 Adapting the intervention to the environment
3.3.1 Fits the in-patient environment neatly
3.3.1.1 Incorporate into a universal admission/discharge bundle
3.3.1.1.1 Use at point of admission and discharge for in-patients
3.3.2 Use the intervention to support the outpatient clinic consultation
3.3.2.1 A record of the consultation conversation
3.3.2.2 A resource for referring back
3.3.3 Complexity in assessing patients remotely in the community

3.4 Training to support implementation
3.4.1 Teams are not static so require ongoing access to training
3.4.1.1 Prioritise junior staff – the people who do the day-to-day work
3.4.1.1.1 Consider including medical students
3.4.1.2 Written information for those unable to attend training
3.4.1.3 Webinar-ed out
3.4.2 Training delivered by vascular team to primary care
3.4.2.1 Involve all primary care staff and not just GPs
3.4.2.1.1 Highlight the link between PAD and cardiovascular death
3.4.2.1.2 Provide education on managing CLTI and non-CLTI patients in primary care
3.4.2.1.3 Taking an ABPI in primary care
3.4.2.1.4 Learning gaps in exercise and lifestyle modification
3.4.2.2 Visibility of vascular teams at primary care conferences
3.4.2.3 Use available supervision and de-briefing opportunities

Theme 4: Personalised information and supportive conversations for taking on the advice

4.1 Offer personalised care and tailor patient information to individual needs
4.1.1 Existing information does not suit all
4.1.1.1 Currently most likely to suit those with IC
4.1.1.2 Targets are impossible to reach
4.1.1.2.1 If I was younger and had more get-up-and-go
4.1.1.2.2 Availability of supervised exercise
4.1.1.2.3 Relying on others for food shopping
4.1.2 A different approach for each patient
4.1.3 Offer realistic targets or the advice rings hollow
4.1.4 Patient education leaflet is provided too late
4.1.4.1 Too far gone to talk about lifestyle modification
4.1.4.1.1 I can only do what I can do
4.1.4.2 Consider co-existing morbidities
4.1.4.3 Offer advice on moderating activity levels and pain management
4.1.4.4 Discuss concerns about medication side-effects

4.2 Everyone knows they need to stop smoking
4.2.1 Acknowledge and support patient preference and promote shared decision making
4.2.1.1 It's easy to bombard someone with information
4.2.1.2 Secondary care doctors tell people to do things
4.2.1.2.1 Consider the language and approach to engage patients
4.2.1.2.2 Be honest and explain the risks
4.2.1.2.2.1 Use available online tools to support the conversation
4.2.1.2.3 Use motivational interviewing techniques
4.2.1.2.4 Frank conversations for some
4.2.2 It's down to willpower at the end of the day
4.2.2.1 I like smoking, I like drinking, I like the telly

4.2.2.2 The only thing left is something nice to eat
4.2.2.2.1 Provide information on the benefits versus taking pleasure away
4.2.2.2.2 Strike a balance to optimise people's quality of life
4.2.2.2.2.1 You can't take everything away
4.2.3 Patient education leaflets are not the best way for everyone
4.2.3.1 I don't want any more leaflets
4.2.3.1.1 District nurses can answer my questions
