# Supplementary material for: Factors that influence the feasibility and implementation of a complex intervention to improve the treatment of peripheral arterial disease in primary and secondary care: a qualitative exploration of patient and provider perspectives
Source: BMJ Open. 2023 Jan 23;13(1):e066883. doi: 10.1136/bmjopen-2022-066883 (PMC9872459; doi:10.1136/bmjopen-2022-066883)
Supplement: Author's manuscript [file bmjopen-2022-066883.draft_revisions.pdf]

BMJ Open is committed to open peer review. As part of this commitment we make the peer review history of every article we publish publicly available.

When an article is published we post the peer reviewers' comments and the authors' responses online. We also post the versions of the paper that were used during peer review. These are the versions that the peer review comments apply to.

The versions of the paper that follow are the versions that were submitted during the peer review process. They are not the versions of record or the final published versions. They should not be cited or distributed as the published version of this manuscript.

BMJ Open is an open access journal and the full, final, typeset and author-corrected version of record of the manuscript is available on our site with no access controls, subscription charges or pay-per-view fees (http://bmjopen.bmj.com).

If you have any questions on BMJ Open's open peer review process please email info.bmjopen@bmj.com

Patient and provider perspectives on factors that influence the implementation of a community and hospital care bundle to improve the treatment of patients with peripheral arterial disease in primary and secondary care.

Journal:	*BMJ Open*
Manuscript ID	bmjopen-2022-066883
Article Type:	Original research
Date Submitted by the Author:	26-Jul-2022

Complete List of Authors:	Le Boutillier, Clair; King's College London<br>Saratzis, Athanasios; University of Leicester<br>Saha, Prakash; Guy's and St Thomas' NHS Foundation Trust<br>Benson, Ruth; University of Birmingham<br>Bridgwood, Bernadeta; University of Leicester<br>Watson, Emma; University of Leicester<br>Lawrence, Vanessa; King's College London, Health Services & Population Research Department

Keywords:	QUALITATIVE RESEARCH, VASCULAR MEDICINE, Change management < HEALTH SERVICES ADMINISTRATION & MANAGEMENT

SCHOLARONE™
Manuscripts

I, the Submitting Author has the right to grant and does grant on behalf of all authors of the Work (as defined in the below author licence), an exclusive licence and/or a non-exclusive licence for contributions from authors who are: i) UK Crown employees; ii) where BMJ has agreed a CC-BY licence shall apply, and/or iii) in accordance with the terms applicable for US Federal Government officers or employees acting as part of their official duties; on a worldwide, perpetual, irrevocable, royalty-free basis to BMJ Publishing Group Ltd ("BMJ") its licensees and where the relevant Journal is co-owned by BMJ to the co-owners of the Journal, to publish the Work in this journal and any other BMJ products and to exploit all rights, as set out in our licence.

The Submitting Author accepts and understands that any supply made under these terms is made by BMJ to the Submitting Author unless you are acting as an employee on behalf of your employer or a postgraduate student of an affiliated institution which is paying any applicable article publishing charge ("APC") for Open Access articles. Where the Submitting Author wishes to make the Work available on an Open Access basis (and intends to pay the relevant APC), the terms of reuse of such Open Access shall be governed by a Creative Commons licence – details of these licences and which Creative Commons licence will apply to this Work are set out in our licence referred to above.

Other than as permitted in any relevant BMJ Author's Self Archiving Policies, I confirm this Work has not been accepted for publication elsewhere, is not being considered for publication elsewhere and does not duplicate material already published. I confirm all authors consent to publication of this Work and authorise the granting of this licence.

For peer review only

Title: **Patient and provider perspectives on factors that influence the implementation of a community and hospital care bundle to improve the treatment of patients with peripheral arterial disease in primary and secondary care.**

Authors: Clair Le Boutillier[1,2], Athanasios Saratzis[3], Prakash Saha[4], Ruth Benson[5], Bernadeta Bridgwood[6], Emma Watson[3,6], Vanessa Lawrence*[1]

[1] Institute of Psychiatry, Psychology & Neuroscience, King's College London, UK
[2] Florence Nightingale Faculty of Nursing, Midwifery & Palliative Care, King's College London, UK
[3] University of Leicester, UK
[4]Guy's and St Thomas' NHS Foundation Trust, UK
[5]University of Birmingham, UK
[6]NIHR Cardiovascular Sciences, University of Leicester, Leicester, UK.

* Corresponding author. Email: vanessa.c.lawrence@kcl.ac.uk

Division of Methodologies, Florence Nightingale Faculty of Nursing, Midwifery & Palliative Care, King's College London, James Clerk Maxwell Building, 57 Waterloo Road, London SE1 8WA. Orcid iD: 0000-0003-3665-0315

Funding

The study was funded by the National Institute for Health Research (NIHR) – Research for Patient Benefit (RfPB) Programme (Reference: NIHR202008).

Category

Original article

For peer review only - http://bmjopen.bmj.com/site/about/guidelines.xhtml

Abstract

Objectives: Our aim was to examine the acceptability and feasibility of a complex intervention to improve the care of patients with Peripheral Arterial Disease (the LEGS intervention) from the perspective of patients, general practitioners and secondary care clinicians.

Design: A qualitative study involving semi-structured individual interviews with patients and providers to gain an understanding of the acceptability, usability, and relevance of the LEGS intervention as well the barriers and facilitators to implementation in secondary and primary care.

Setting: Primary and secondary care settings across two NHS Trusts.

Participants: Twenty-five semi-structured telephone interviews were conducted with i) patients who had received the intervention (n=11), ii) secondary care clinicians responsible for delivering the intervention (n=8), and iii) general practitioners (n=6).

Analysis Data were initially analysed using inductive descriptive thematic analysis. The consolidated framework for implementation research was then used as a matrix to explore patterns in the data and to map connections between the three participant groups. Lastly, interpretive analysis allowed for refining, and a final coding frame was developed.

Results: Four overarching themes were identified: i) The potential to make a difference, ii) A solution to address the gap in no man's land, iii), Prioritising and making it happen and iv) Personalised information and supportive conversations for taking on the advice. The impetus for prioritising and delivering the intervention was further driven by its flexibility and adaptability to be tailored to the individual and to the environment.

Conclusions: The LEGS intervention can be tailored for use at early and late stages of Peripheral Arterial Disease, provides an opportunity to meet patient needs and to promote shared working across the primary-secondary care interface.

For peer review only

Strengths and limitations

- A strength of this study is the focus on rigor and triangulation of participant perspectives.
- We used implementation theory to guide analysis and to provide a layered coding approach, to ensure that intervention and implementation recommendations are novel, grounded in the data, and useful (1).
- While the anonymity of telephone use can allow participants to disclose sensitive information, telephone interviews have received criticism for compromising interviewer/participant rapport and interaction, and for limiting contextual data due to the absence of face-to-face contact and visual cues.
- However, this method of data collection is convenient, in that it is flexible (in terms of time and location), accessible (i.e., remote research conducted during the COVID-19 pandemic), and allows for a wide reach (e.g., accessing a diverse population from both urban and rural areas) (2).

For peer review only

List of abbreviations

PAD	Peripheral Arterial Disease
IC	Intermittent Claudication
CLTI	Chronic Limb Threatening Ischaemia
GP	General Practitioner
CHABLIS	Community and Hospital cAre Bundle to improve the medical treatment of cLaudIcation and critical limb iSchaemia
LEGS	LEaflet Gp letter Structured checklist
UHL	University Hospitals of Leicester NHS Trust
GSTT	Guy's and St Thomas' NHS Foundation Trust
CFIR	The consolidated framework for implementation research
NHS	National Health Service
ODS	Online Data Supplement

For peer review only - http://bmjopen.bmj.com/site/about/guidelines.xhtml

Background

Peripheral Arterial Disease (PAD) affects a fifth of people over the age of sixty in the United Kingdom. This prevalence is also expected to rise due to sedentary lifestyles, poor dietary choices, a longer life-expectancy, and rising numbers of those with diabetes (3, 4). Patients with PAD who develop symptoms present either with Intermittent Claudication (IC) or in some cases, Chronic Limb Threatening Ischaemia (CLTI) where the reduction in blood flow is so severe that it causes pain at rest, ulceration, or gangrene (5). Patients with IC are more likely to suffer a cardiovascular event compared to age-matched individuals, and the one-year risk of limb amputation for those with CLTI is 30%. More than half of those diagnosed with symptomatic PAD are expected to die, have an amputation or a major cardiovascular event within five years (6-9).

The treatment of these high-risk individuals is shared between primary and secondary care. While established national and international guidance (10) outlines how PAD risk-factors should be addressed to prevent cardiac events and amputations, a study conducted by our group found that only a tenth of those diagnosed with PAD were prescribed appropriate preventive medication, and almost none received structured lifestyle or dietary support (11). As a result, and following patient and public engagement (that is, interviews and workshops with patients and a national consultation with primary and secondary care clinicians) and a review of the existing evidence, a complex intervention in the form of a care bundle was developed to support patients, General Practitioners (GPs), and secondary care clinicians to actively manage cardiovascular risk-factors, and to improve implementation of existing guidance when treating patients with PAD. The care bundle, called the LEGS intervention (Leaflet, Gp letter, Structured checklist) has three elements: i) a one-page clinical checklists for the ward and a separate one-page clinical checklist for the secondary care clinic, ii) a patient and carer education leaflet (developed with patients), iii) a GP action letter, that can be sent to GPs at regular intervals to inform them of the PAD diagnosis and corresponding

For peer review only - http://bmjopen.bmj.com/site/about/guidelines.xhtml

practice guidance. The LEGS intervention and full study protocol are available online (isrctn.com/ISRCTN13202085).

This qualitative study was embedded within the Community and Hospital cAre Bundle to improve the medical treatment of cLaudIcation and critical limb iSchaemia (CHABLIS) study. CHABLIS is a multicentre prospective mixed-methods cohort study that aims to determine the feasibility and effectiveness of using a PAD care bundle, called the LEGS intervention (LEaflet Gp letter Structured checklist). The aim of the qualitative study was to gain an understanding of the acceptability of the LEGS intervention, by exploring patient and provider perspectives on receiving and delivering the intervention (12). The objectives of the qualitative study were to 1) to explore patient perspectives on receiving the LEGS intervention, 2) to gather secondary care clinician views on acceptability and usability, and to understand factors that influence the success of implementation, and 3) to identify barriers and facilitators that influence the success of future implementation from the GP perspective.

METHODS

Study design

The study used qualitative methods to provide insights into the acceptability, usability, and relevance of the intervention as well as the complexities of implementation. Semi-structured individual interviews were conducted with patients and providers to gain an understanding of the barriers and facilitators to implementation in secondary and primary care.

To meet objective 1, semi-structured individual interviews were conducted with patients with symptomatic PAD (i.e., IC, ischaemic rest pain, or CTLI), who had received the intervention (either in an outpatient clinic or as a part of inpatient treatment). To meet objective 2, semi-structured individual interviews were conducted with secondary care clinicians responsible for delivering the intervention, including junior doctors, vascular nurses, and vascular surgeons. Staff and patients were recruited from two implementation sites: University Hospitals of

For peer review only - http://bmjopen.bmj.com/site/about/guidelines.xhtml

Leicester NHS Trust (UHL) and Guy's and St Thomas' NHS Foundation Trust (GSTT). To meet objective 3, semi-structured individual interviews were conducted with GPs to gain an understanding of the barriers and facilitators to future implementation in primary care and across the NHS. The research was reviewed and approved by the NHS Wales Research Ethics Committee (REC), reference number20/WA/0319 and the NHS Health Research Authority (HRA).

Participants were selected purposively (both those who were receiving or responsible for delivering the LEGS intervention) on the basis that they are the target adopters of behaviours that influence the success of future implementation and/or could offer a particular perspective on implementation. Participants were over 18, and proficient in English. All patients who received the intervention between June and October 2021 were eligible to take part. Eligible patients were approached and recruited by the local research team (for example, local principal investigator, research nurse), via face-to-face consultation as a part of outpatient clinic or hospital admission. All secondary care clinicians responsible for delivering the intervention were invited to take part by the local research team. GP participants were recruited from Clinical Commissioning Groups linked to the two secondary care implementation sites, chosen to enhance joined up working and to provide a local perspective on implementation across community and hospital settings. Potential GP participants were first approached by the local Clinical Research Network research delivery manager or local contacts, and subsequently recruited by the lead author via telephone or email. Written or verbal informed consent was obtained from each participant after a full explanation and information leaflet was given and time allowed for consideration. The right of each participant to refuse to participate without giving reasons was respected. All participants were free to withdraw from the study at any time without giving reasons and without prejudicing further treatment or employment.

Data collection

For peer review only - http://bmjopen.bmj.com/site/about/guidelines.xhtml

Interviews used flexible open-ended questions for early data collection to gather a rich and detailed understanding of participants' perspectives. The patient and provider interview schedules were revised iteratively in response to the priorities and concerns of participants and are included in Online Data Supplement 1. Interviews were conducted by telephone by the lead author (a qualitative research fellow), lasted around 45 minutes, were audio-recorded and transcribed verbatim. Researcher reflexive notes were kept after each interview to consider the interaction with the participant, and to detail initial thoughts. Interviews were conducted between September and November 2021.

Data Analysis

Data from the three participant groups (patients, secondary care clinicians and GPs) were first analysed separately using inductive thematic analysis, where analytical concepts and perspectives are generated from the data in a deliberate and systematic way (Braun & Clarke, 2006). Data analysis began with repeated re-reading of individual transcripts and re-listening of sound files for data immersion. This was followed by line-by-line open coding, where extracts were coded under one or several descriptive themes, that is organising the data according to semantic content, to capture their meaning and reflect the content of the data. Each theme was refined, and where data allowed, further sub-themes were developed (CL). The Consolidated Framework for Implementation Research (CFIR) was then used as a matrix to organise the early themes, to explore patterns and relationships in the data, and to map connections between the themes and the three participant groups (Damschroder et al, 2009). Lastly, interpretive analysis allowed for the refining of the specifics of themes and thematic patterns, and a final coding frame for patients and providers was developed. Refinements to the specifics of themes, and thematic patterns continued until a useful and meaningful analysis was achieved (13).

Data collection occurred concurrently with data analysis; NVivo QSR International qualitative analysis software (version 12) was used to manage the data in a way that supported data

For peer review only - http://bmjopen.bmj.com/site/about/guidelines.xhtml

analysis (14). The lead author directed the analysis. Coding by a second rater (VL) was undertaken to provide an opportunity to reflect on the coding approach, and to enhance the interpretive depth of the data (13).

Patient and public involvement

The LEGS intervention and study design were developed in PPI development sessions with eight patients with PAD. Ongoing PPI support was given by a PPI co-applicant who helped interpret the qualitative findings.

RESULTS

Participants

A total of 25 individual interviews were conducted with i) patients who had received the intervention (n=11), ii) secondary care clinicians responsible for delivering the intervention (n=8), and iii) GPs (n=6). Two of the eight secondary care clinicians were actively delivering the intervention (in an in-patient setting), and all GPs provided insights on future implementation. Six patients had received the intervention as a part of in-patient care and five patients provided perspectives on receiving the intervention from out-patient vascular, ulcer, or specialised CLTI clinics. Patient participants spoke about living with multiple conditions including diabetes, stroke, and amputation and receiving care from primary care (GPs, district nurses, diabetic nurses) secondary care (vascular teams), social care (carers), and family. Three patients were >70 years old but all patients were retired or unemployed and spoke about the impact of PAD on quality of life including managing pain, slow wound healing, poor sleep, reduced daily functioning, and limited mobility. Patient characteristics are shown in Table 1 and provider characteristics are detailed Table 2.

For peer review only - http://bmjopen.bmj.com/site/about/guidelines.xhtml

Table 1: Patient participant characteristics

**Patient characteristics** n (%)	Patients n=11
**Age**	
41-50 years	1 (9.1)
51-60 years	0 (0.00)
61-70 years	2 (18.2)
>70 years	8 (72.7)
**Gender**	
Male	8 (72.7)
Female	3 (27.3)
**Ethnicity**	
White British	9 (81.8)
White Irish	1 (9.1)
Black/Black British-Caribbean	1 (9.1)
**Marital status**	
Single	2 (18.2)
Married	7 (63.6)
Widowed	2 (18.2)
**Time since diagnosis**	
>1 year	3 (27.3)
1-4 years	4 (36.35)
5-9 years	0 (0.00)
10 years +	4 (36.35)
**No. of surgical interventions**	
None	1
Angioplasty	8
Bypass graft	4
Amputation	2

Table 2: **Provider participant characteristics**

**Provider characteristics** n (%)	Hospital staff n=8	GPs n=6
**Age**		
21-30 years	3 (37.5)	1 (16.7)
31-40 years	5 (62.5)	3 (50.0)
41-50 years	0 (00.0)	2 (33.3)
**Gender**		
Male	4 (50.0)	4 (66.7)
Female	4 (50.0)	2 (33.3)
**Ethnicity**		
White British	2(25.0)	3 (50.0)
White Other	1 (12.5)	1 (16.7)
Asian/Asian British-Indian	2 (25.0)	2 (33.3)
Mixed. White & Asian	1 (12.5)	0 (00.0)
Chinese	1 (12.5)	0 (00.0)
Other	1 (12.5)	0 (00.0)
**Core Profession**		

For peer review only

Nurse	1 (12.5)	0 (00.0)
Vascular surgeon	7 (87.5)	0 (00.0)
GP trainee	0 (00.0)	1 (16.7)
General practitioner	0 (00.0)	5 (83.3)
**Grade**		
Band 8a	1 (12.5)	0 (00.0)
Senior House Officer	2 (25.0)	0 (00.0)
Specialty trainee	1 (12.5)	1 (16.7)
Speciality registrar	2 (25.0)	0 (00.0)
Consultant	2 (25.0)	0 (00.0)
GP	0 (00.0)	5 (83.3)
**Time since qualification**		
0-2 years	0 (00.0)	1 (16.7)
2 years + -5 years	0 (00.0)	1 (16.7)
5 years + -10 years	3 (37.5)	2 (33.3)
10 years + -15 years	5 (62.5)	2 (33.3)
**Time in current post**		
>6 months	5 (62.5)	1 (16.7)
7-12 months	0 (00.0)	0 (00.0)
13-24 months	2 (25.0)	2 (33.3)
25-36 months	0 (00.0)	1 (16.7)
37-48 months	1 (12.5)	0 (00.0)
49-60 months	0 (00.0)	0 (00.0)
61-75 months	0 (00.0)	1 (16.7)
13 years	0 (00.0)	1 (16.7)

Of the patient participants contacted by the researcher (CL), 42% agreed to take part in an interview (fifteen people declined) and 82% of staff agreed to take part (1 person declined participation and 4 people did not respond to the invitation). Reasons for non-participation for patients included being unable to remember the intervention (n=5), deteriorating health (n=7), just home from an in-patient admission (n=1), being admitted for angioplasty (n=1) and getting back on with life (n=1). The reason provided by providers for declining participation was moving to a new role. Secondary care clinicians and GPs spoke about all three elements of the intervention and patients provided insights on the education leaflet and GP action letter. The descriptive themes were mapped to implementation theory using the CFIR, to explore connections between the themes, and to compare findings across participant groups. Four of the five overarching CFIR domains are represented across participant groups: intervention characteristics, outer setting, inner setting, and characteristics of individuals. The mapping of

For peer review only

themes across CFIR domains and participant groups is included in Online Data Supplement 2.

For intervention characteristics, all participant groups spoke about the relative advantage, adaptability, and design, quality, and packaging of the education leaflet, as well as the complexity in implementing the GP action letter. For outer setting, patient needs and resources (including barriers and facilitators to meet needs) were highlighted, with all participant groups explaining that a delayed referral to secondary care is a barrier to implementing the intervention. For inner setting, structural characteristics and networks and communications were identified by all groups as influences on implementation. For structural characteristics, participants explained that the ability to follow the advice in the education leaflet is often determined by the availability of supervised exercise programmes, difficulty ordering medication, and relying on carers for food shopping. For networks and communications, participants spoke about the need to work as a three-way team, and to have ongoing access to the specialist vascular team. For characteristics of individuals, staff values, attitude, knowledge, and skills to provide the intervention and patient readiness to take on the advice of the intervention were highlighted across participant groups. There was a particular emphasis on the individual stage of change sub-domain, with participants highlighting a need to engage patients in their care and to promote shared decision making. The patient-healthcare provider relationship was identified as a central influence relating to individual identification within the organisation, that is, how patients and staff perceive the organisation and their relationship with that organisation).

A final coding frame for patients, secondary care clinicians and GPs was developed following interpretive analysis. Because of space limitations, a summary of each theme is provided, and the subthemes are not elaborated in this article; the full coding framework is included in Online Data Supplement 3. Four overarching themes were identified: *the potential to make a difference, a solution to address the gap in no man's land, prioritising and making it happen*

For peer review only - http://bmjopen.bmj.com/site/about/guidelines.xhtml

and *personalised information and supportive conversations for taking on the advice.* Similarities and differences between participant groups are presented where they arise.

Theme 1: The potential to make a difference

GPs and secondary care clinicians felt that the LEGS intervention provides an opportunity to meet patient need and to support implementation of NICE guidance and spoke optimistically about using the intervention to improve patient outcomes and to standardise a minimum quality of care.

> *…it standardises the quality of care and standardisation is a big problem that we have in vascular surgery because guidance is so vague. So, I think it raises awareness and standardises that at least we ensure the patient gets a minimum of care* (QR15, secondary care clinician, UHL).

GPs and secondary care clinicians went on to explain how the intervention can be used to inform, empower, and support relationship building with patients.

> *I've gone through the leaflet with them… and knowing that the patient has understood I will spend a couple of minutes going through the leaflet to make sure. If they've got any questions I will try and take the opportunity to build my relationship with the patient so that they do understand* (QR08, secondary care clinician, UHL).

To further improve quality of care, providers identified the intervention as a way to support shared working across primary and secondary care, promoting a team approach and providing an opportunity for collaborative working. One GP explained how the '*patient is in the middle'* (QR23) and a secondary care clinician felt that '*we don't sing off the same hymn sheet'* (QR08). One GP explained that w*e don't ever see secondary care specialists* and a secondary care clinician explained *there's always this assumption that the GP will sort it out* (QR13). It was acknowledged that shared working provides an opportunity to understand each other's roles, to manage expectations and to learn from one another. Providers went on to report patient benefits in terms of access to specialist clinicians and working as a multi-disciplinary team, *I've always found that patients who have the opportunity to talk to multiple members of the medical team rather than a doctor or a nurse come out with better outcomes* (QR08, secondary care clinician, UHL).

For peer review only - http://bmjopen.bmj.com/site/about/guidelines.xhtml

Patients also spoke about the benefit of joined-up working, with one person stating;

> *…you attend lots of different clinics, you've got different people involved in your healthcare. It's not just one thing and everything's linked… it's what they call whole person care. It's not just your blood pressure, it's also the vascular team, it's the podiatrist, it's the diabetes specialist* (QR07, out-patient clinic, GSTT).

Theme 2: A solution to address the gap in no man's land

All participant groups spoke about a need to target patients earlier and identified a service provision gap in preventative medicine and early diagnosis. One secondary care clinician stated;

> *I think, almost targeting people earlier on, so claudicants would be really good… claudicants at the very first stage of their peripheral arterial disease, they're not really managed in secondary care, but they're also not really managed in primary care… this gap where they're in no man's land and they almost wait until it's severe enough to managed into secondary care* (QR18, secondary care clinician, UHL).

Patients explained how it took time to be referred to secondary care, and in one person's situation, it became an emergency;

> *…my doctor prescribed pills and some cream for my foot but they didn't do anything. And then it was only when I went back to podiatry after three months of constant pain, it was a case of just wait and see how things went… I went one day in the November, and they said you need to have your toe off almost immediately… they sent me to [hospital] and [hospital] say 'your toe needs to come off and it needs to come off in 20 minutes time'* (QR04, In-patient care, UHL).

Difficulties with patients presenting late, high secondary care referral thresholds resulting in delayed referrals to secondary care and a wait for secondary care to make the diagnosis, alongside lack of support for primary care to commence diagnostics in the community were also listed as barriers to accessing timely care. Some secondary care clinicians felt that the care of those with IC should be managed in primary care.

> *I think even the way that if the GP could start the patient, the first medical therapies, like, clopidogrel and statins, if he also could start the patient on exercise therapies. So, it should be implemented at the same kind of wave of the first medical therapy.* (QR19, secondary care clinician, UHL).

GPs offered their support and made a request to do more sooner. Some suggested using the intervention in general practice as part of a PAD prevention programme. They acknowledged the value of using the intervention to promote earlier conversations with patients, manage patient expectations, and support primary care to start best medical therapy. GPs also spoke

For peer review only - http://bmjopen.bmj.com/site/about/guidelines.xhtml

about how being involved sooner, and completing the clinical checklist, could be used to support triage of referrals to secondary care. GPs noted that preventative medicine and health promotion is core to their practice, and that sharing information across services and with patients reinforces the message and supports implementation.

Theme 3: Prioritising and making it happen

Secondary care clinicians spoke about the need to prioritise the intervention alongside limited resources and the burden of work. One secondary care clinician explained, *it shouldn't be seen as an extra thing* (QR08). Individual levels of confidence, attitude, knowledge and experience of the LEGS bundle, or a similar checklist/patient education intervention influenced prioritisation with some staff identifying checklists as a quick and easy-to-use memory prompt. However, another reported that;

> *…people feel that it's just a tick-box exercise rather than a safety mechanism, and therefore no engagement with it… [it's] another piece of paper… I think part of the challenge is engagement with clinicians. Clinicians don't like threats to their autonomy… doctors, surgeons in particular are reticent, or certainly some are reticent to engage with checklists because they feel that it is doubling down or they already know this or that, you know, this simplifies things* (QR13, secondary care clinician, UHL).

Secondary care clinicians spoke about the extra resources required to deliver the intervention; it takes time to build relationships and to have conversations with patients. One secondary care clinician stated that finding the time to talk would require extra staff, this in an already stretched environment with staff shortages and long waiting times.

> *That will take more time, to make it personalised, to make it meaningful… you could almost imagine that they have a consultation and then some of those patients might benefit from sitting down with one of the specialist nurses… you could almost benefit from a 20-minute appointment after the standard clinic appointment to go through all of that. But again, that's a resource burden, that means that you've got to have someone extra in clinic* (QR13, secondary care clinician, UHL).

Secondary care clinicians felt that the intervention fits the in-patient environment neatly and could be used as a record of the out-patient clinic consultation. GPs felt that it could support the complexity of assessing patients remotely in the community. The need for training was recognised;

For peer review only - http://bmjopen.bmj.com/site/about/guidelines.xhtml

> *I think there is a huge lack in primary care education, and I think that goes to show by the starting people coming in are only on like a 40% of them are on Best Medical Therapy so I think a big thing to target would be primary care to inform them* (QR18, secondary care clinician, UHL).

GPs went on to request that all primary care staff be involved and not just GPs, and that the primary care training be delivered by the vascular team.

Theme 4: Personalised information and supportive conversations for taking on the advice

Providers and patients spoke about the delivery of the intervention, recognising the importance of offering personalised care and tailoring patient information to individual needs. Secondary care clinicians felt that the intervention should be adapted to identify realistic targets and individual goals, *or the advice rings hollow* (QR13), and to offer a different approach for each patient. Staff reported;

> *I think the hard thing as well is that with the LEGS bundle, sometimes you're giving it to someone who's just had an amputation or about to have an amputation so it's just to be mindful you're talking about things like exercise they look at you very disparagingly and that can be a bit tricky… I think it's a really good checklist and bundle for claudicants and I think that's where it really shines but I think for when you get to the stage of CLTI, I think those individuals often feel that they're too far gone for you to talk to them about lifestyle modification and things like that* (QR18, secondary care clinician, UHL).

Patients also spoke about the importance of timing in terms of when they received the lifestyle information.

> *If I was a bit younger and I'd got more get-up-and-go, I would gladly have a leaflet and go through it and take up advice and what have you. But as I am at the moment, my health is far from good* (QR14, in-patient care, UHL).

> *…it is a bit too late in that respect so I can only do what I can do. I mean yesterday I was out in the garden doing some weeding but I have to use a seat to do it* (QR05, in-patient care, UHL).

Patients explained that they like to receive information face-to-face and suggested that the education leaflet could be used as a supportive conversation guide. They emphasised the importance of the conversation that goes alongside the advice and information, and the

For peer review only - http://bmjopen.bmj.com/site/about/guidelines.xhtml

relationship that they have with their health professional(s) in terms of asking questions and being able to talk about making changes like smoking cessation. One patient explained,

> *… the only thing I've got to do is drink and smoke and watch the tv – cause if I didn't have those three things, I would be pulling my hair out… to be quite honest, I'm quite happy at the moment to be carrying on the way I am. It might sound silly to you, but I enjoy smoking. I love a drink, I like my telly… if you're brow-beaten into packing up when you don't really want to, all this, it can have a great effect on people* (QR14, in-patient care, UHL).

GPs and secondary care clinicians acknowledged the need to support patient preference, to involve patients in the conversation and to share decision making.

> *…you have to strike a balance, and therefore taking the patient's preferences into account is important. We assume that patients want to live the longest life they can, I'm not sure that's always correct. Some patients would rather accept that they live a slightly shorter life but with the benefit of enjoying certain things that we all know are less healthy* (QR13, secondary care clinician, UHL).

It was acknowledged that everyone knows they need to stop smoking and it is easy to bombard people with information. Solutions such as motivational interviewing techniques were identified by one GP as a way to support engagement conversations. Secondary care clinicians felt that providing information on the benefits of following the advice versus taking pleasure away might help. For others, frank conversations that explain the risks help;

> *I think honesty is the most important aspect of this and bringing people in front of the data, highlighting the data, and actually not sugar coating anything. We know, for example, that if you have critical ischemia, so severe peripheral arterial disease, if your prognosis is worse, your five-year survival is worse than some kinds of cancer. So, if you tell someone that unless you actually take this intervention seriously, your likelihood of not being around in five years is very high. I know it sounds bleak, but it may be the wake-up call that someone needs. Or explaining that from the moment you have severe arterial peripheral disease, the likelihood of you losing your leg and being wheelchair bound may be an important thing to highlight as opposed to speaking in a circumspect way. I think just being honest and explaining the risks may be a way of engaging patients.* (QR15, secondary care clinician, UHL).

A patient explained how the honest frank information helped;

> *The doctor actually threatened me. He said to me, if I'm going to do this operation, you got to stop smoking. So, I did. That helped, or I could lose my leg. I thought it was brilliant the way [the doctor] come out with it and I've never stopped thanking him for it. It works* (QR06, out-patient care, GSTT).

Discussion

For peer review only - http://bmjopen.bmj.com/site/about/guidelines.xhtml

The aim of the study was to provide an understanding of the acceptability of the LEGS (LEaflet Gp letter Structured checklist) intervention, a complex intervention in the form of a healthcare bundle, designed to improve the care of patients with PAD in the NHS. Our inductive approach to analysis found four overarching themes: i) *the potential to make a difference, ii) a solution to address the gap in no man's land, iii) prioritising and making it happen* and *iv) personalised information and supportive conversations for taking on the advice.* We used the Consolidated Framework of Implementation Research (CFIR) to guide our analysis, and all five CFIR domains are represented (1).

All participants spoke positively about using the intervention to make a difference to patient care and as an opportunity to meet patient needs. Moreover, a way to enhance the quality of PAD care through improved working practices. This promotes a streamlined patient pathway from primary care to secondary care and to shared care. There was a consensus of optimism in participant accounts about the potential to make a difference. The findings confirm that the period between diagnosis of early-stage PAD (e.g., claudication) and subsequent potential diagnosis of advanced PAD with severe symptoms, is a time when patients do not receive any help, education, support, or appropriate medication to address their condition (11). As such, the benefits of the LEGS intervention were identified in terms of meeting the needs of this subgroup of patients, and to address the gap in care. GPs were optimistic that they can deliver the LEGS intervention with training and ongoing sustained communication with secondary care. The findings highlight the need to consider training requirements and systems for maintaining open primary-secondary care communication to support implementation. For example, education on tailoring the intervention for patients at both early and late stages of PAD, that can be used across primary and secondary care settings, similar to the model used with other chronic conditions (15).

While the three elements of the LEGS intervention have been designed to facilitate interaction between secondary and primary care following diagnosis of PAD, the intervention focus until

For peer review only - http://bmjopen.bmj.com/site/about/guidelines.xhtml

now, has been on secondary care services, that is, hospital admission or out-patient clinic. However, our findings indicate that the intervention can be used to address the gap in service provision for patients who do not yet meet the referral criteria for secondary care services. We therefore propose to extend the use of the LEGS intervention across the primary-secondary care interface. Our findings also confirm the need for individualised patient care, supportive communication and shared decision making in order to take on the advice in a more effective way (16). The finalised LEGS intervention should therefore promote effective conversation between patient and healthcare staff, both in primary and secondary care settings. The open approach to changing lifestyle and behaviour could be built on techniques like motivational interviewing, which is already commonly used in primary care practice (17). Lastly, our findings confirm that healthcare providers are optimistic and positive about the introduction of an intervention which will help them to deliver more consistent and guideline-based PAD treatment. The challenge now is to take this enthusiasm together with the lessons learned from this qualitative research to adapt the LEGS intervention before wider testing and adoption in the NHS. The main changes will relate to usability in primary care after diagnosis of early-stage PAD, facilitation of effective communication not only between primary and secondary care staff, but also patients and staff, as well as focussing on specific needs of patients by prioritising areas to focus on in an incremental or stepwise manner.

Conclusions

The LEGS intervention can be tailored for use at early and late stages of PAD, can be provided across primary and secondary care settings, and provides an opportunity to promote shared working across the primary-secondary care interface. These findings are important to the specifics of the CHABLIS study but can also go some way in informing the wider learning for other interventions that are implemented across the primary-secondary care interface.

Strengths and limitations

For peer review only

For peer review only - http://bmjopen.bmj.com/site/about/guidelines.xhtml

The paper adds new knowledge on the development and implementation of a community and hospital intervention for Peripheral Arterial Disease. It is important to note that the findings are specific to the CHABLIS study. It is possible, however, to enhance transferability by describing the research context and assumptions, and by making connections between the analysis of participants accounts and claims in the extant literature. A strength is the focus on rigor and triangulation of participant perspectives. We also acknowledged the complexity of intervention implementation across the secondary-primary care interface and therefore used a systems research perspective for addressing implementation at the outset of the research. We used implementation theory to guide analysis and to provide a layered coding approach, to ensure that intervention and implementation recommendations are novel, grounded in the data, and useful (1).

While the anonymity of telephone use can allow participants to disclose sensitive information, telephone interviews have received criticism for compromising interviewer/participant rapport and interaction, and for limiting contextual data due to the absence of face-to-face contact and visual cues. However, this method of data collection is convenient, in that it is flexible (in terms of time and location), accessible (i.e., remote research conducted during the COVID-19 pandemic), and allows for a wide reach (e.g., accessing a diverse population from both urban and rural areas) (2).

Funding

This article presents independent research funded by National Institute for Health Research for Patient Benefit (RfPB) Programme (funder’s reference: NIHR202008). The views expressed are those of the author(s) and not necessarily those of the NIHR or the Department of Health and Social Care.

Competing interests

The authors declare that they have no competing interests.

Authors' contributions

For peer review only

For peer review only - http://bmjopen.bmj.com/site/about/guidelines.xhtml

CL contributed to the qualitative study design, coordinated the study, led data collection and analysis, and drafted the manuscript. AS is principal investigator to the CHABLIS study, acquired funding, participated in the study design, contributed to data collection, and drafted the manuscript. PS contributed to data collection and reviewed the manuscript. RB and BB drafted the manuscript. EW contributed to data collection and reviewed the manuscript. VL designed the qualitative study, provided oversight on study coordination, data collection and analysis, and drafted the manuscript. All authors read and approved the final manuscript.

Consent for publication

Consent for publication has been obtained from all research participants.

Word count

4,775

Availability of data and materials

This study uses data (containing potentially identifying and/or sensitive information) collected from a small group of staff participants and a vulnerable patient population, and involves indirect identifiers (such as sex, ethnicity, location, etc.) that may risk the identification of study participants. Sharing data outside of the anonymised excerpts and quotations included in the paper will violate the agreement to which the participants consented.

Acknowledgements

We would like to thank all patients, secondary care staff and GPs who participated in this study and who generously gave their time and honest thoughts. We would like to thank the local research teams for their support in recruiting to this qualitative study and we are also grateful to the NIHR Clinical Research Network Research Delivery Managers that helped to recruit GP participants.

Ethics Statement

The research was reviewed and approved by the NHS Wales Research Ethics Committee (REC), reference number20/WA/0319 and the NHS Health Research Authority (HRA).

References

For peer review only

1. Damschroder LJ, Aron DC, Keith RE, Kirsh SR, Alexander JA, Lowery JC. Fostering implementation of health services research findings into practice: a consolidated framework for advancing implementation science. Implement Sci. 2009;4:50.

2. Irvine A. Duration, Dominance and Depth in Telephone and Face-to-Face Interviews: A Comparative Exploration. International Journal of Qualitative Methods. 2011;September:202-20.

3. Herrington W, Lacey B, Sherliker P, Armitage J, Lewington S. Epidemiology of Atherosclerosis and the Potential to Reduce the Global Burden of Atherothrombotic Disease. Circ Res. 2016;118(4):535-46.

4. Cea-Soriano L, Fowkes FGR, Johansson S, Allum AM, García Rodriguez LA. Time trends in peripheral artery disease incidence, prevalence and secondary preventive therapy: a cohort study in The Health Improvement Network in the UK. BMJ Open. 2018;8(1):e018184.

5. Dhaliwal G, Mukherjee D. Peripheral arterial disease: Epidemiology, natural history, diagnosis and treatment. Int J Angiol. 2007;16(2):36-44.

6. Parvar SL, Fitridge R, Dawson J, Nicholls SJ. Medical and lifestyle management of peripheral arterial disease. J Vasc Surg. 2018;68(5):1595-606.

7. Nehler MR, Duval S, Diao L, Annex BH, Hiatt WR, Rogers K, et al. Epidemiology of peripheral arterial disease and critical limb ischemia in an insured national population. J Vasc Surg. 2014;60(3):686-95.e2.

8. Dua A, Lee CJ. Epidemiology of Peripheral Arterial Disease and Critical Limb Ischemia. Tech Vasc Interv Radiol. 2016;19(2):91-5.

9. Morley RL, Sharma A, Horsch AD, Hinchliffe RJ. Peripheral artery disease. Bmj. 2018;360:j5842.

10. National Institute for Health and Care Excellence. Peripheral arterial disease: diagnosis and management. 2020.

11. Saratzis A, Jaspers NEM, Gwilym B, Thomas O, Tsui A, Lefroy R, et al. Observational study of the medical management of patients with peripheral artery disease. Br J Surg. 2019;106(9):1168-77.

12. Skivington K, Matthews L, Simpson SA, Craig P, Baird J, Blazeby JM, et al. Framework for the development and evaluation of complex interventions: gap analysis, workshop and consultation-informed update. Health Technol Assess. 2021;25(57):1-132.

13. Braun V, Clarke V. One size fits all? What counts as quality practice in (reflexive) thematic analysis? Qualitative Research in Psychology. 2021;18(3):328-52.

14. QSR International Pty Ltd. NVivo. 2018.

15. Smith SM, Cousins G, Clyne B, Allwright S, O'Dowd T. Shared care across the interface between primary and specialty care in management of long term conditions. Cochrane Database Syst Rev. 2017;2(2):Cd004910.

16. Barry MJ, Edgman-Levitan S. Shared decision making--pinnacle of patient-centered care. N Engl J Med. 2012;366(9):780-1.

17. Rubak S, Sandbaek A, Lauritzen T, Christensen B. Motivational interviewing: a systematic review and meta-analysis. Br J Gen Pract. 2005;55(513):305-12.

For peer review only

For peer review only - http://bmjopen.bmj.com/site/about/guidelines.xhtml

Interview Topic Guide for Patients

Title of the study: A **C**ommunity and **H**ospital c**A**re **B**undle to improve the medical treatment of c**L**aud**I**cation and critical limb i**S**chaemia. **(**The CHABLIS study)

Welcome and introduction

[Seek consent to continue and to audio-record the interview (if applicable). Participant has to provide written informed consent (see separate form). Let them know that no personal identifiable data will be recorded and a participant number will be allocated to them.]

The main aim of this study is to assess whether a dedicated intervention (a care bundle) can be used in the routine care of patients who present with symptomatic Peripheral Arterial Disease (PAD). The care bundle includes a number of checklists and leaflets for doctors and patients. The intervention is aimed at reducing the risk of major cardiovascular events (i.e. heart attacks and strokes), and preventing amputation, by making sure patients with PAD receive the appropriate treatment and management as recommended by national guidelines. We have called this intervention LEGS (the LEGS bundle), given that the aim is to save legs from amputation and improve vascular care.

The LEGS intervention:

ELEMENT 1: In-patient checklist/Outpatient Checklist

The doctors who see you will be given a checklist of the common things that patients with PAD should be offered in terms of medications and / or tests. All of these suggestions are already part of what is the standard NHS care for PAD. Using your medical notes, we will record your blood pressure, your medications (current), you medical history, previous surgeries, imaging results, your routine blood test results (cholesterol, lipid profile, full blood count), smoking status, and demographic information (age, height, weight, gender). This is standard information that the NHS already collects in order to be able to offer you treatment for your blocked arteries.

ELEMENT 2: 2nd (follow-up) letter to the GP

ELEMENT 3: Patient & carers' leaflet

You will be given a leaflet which will provide you with information about PAD and other things you can do to help reduce your risks.

You will have an appointment to see us again in six months to check your legs and your overall health. This is standard for someone with PAD. We will again record your blood pressure, your medications (current), your medical history, previous surgeries, imaging results, your routine blood test results (cholesterol, lipid profile, full blood count), smoking status, and demographic information (age, height, weight, gender). During that appointment we will ask you to fill in a questionnaire which assesses how well people are taking their prescribed medication, called the MARS questionnaire.

For peer review only - http://bmjopen.bmj.com/site/about/guidelines.xhtml

The interview will explore your experience of receiving the intervention. We will ask about your opinion of NHS care relating to your leg artery problems in greater detail. We will ask you how you have found the management of your care at hospital and your GP practice, what your thoughts are of the information leaflet, and if you think it has been of any benefit to you or not.

Questions:

1. How have you found the management of your care at hospital and your GP practice?

- the initial consultation between patient and healthcare professional after the diagnosis of peripheral arterial disease has been made
  - what should be covered in the initial consultation?
  - Anything missing from this consultation?
  - Are you able to ask questions?

- the frequency and duration of follow-up

2. What are your thoughts on the information leaflet?

- Is there enough information about peripheral arterial disease? Too much information? Is the information clear? Signposts for where to go for more information?
- Is there anything on the leaflet that you don't understand or would like more information about?
- What would you like to see included in a leaflet designed to inform patients about peripheral arterial disease care and therapies?
- What about the length of the leaflet?

3. Do you think it has been of any benefit to you or not?

4. In your opinion, are there any factors that might influence someone's ability to follow the guidance in the patient information leaflet? (i.e. barriers to taking medication, managing diet)

- Is there anything that could help? Solutions to any barriers for following the advice?

5. Any ideas for improving any of the elements of the intervention?

   ELEMENT 1: Inpatient/outpatient checklist (& patient consultation)
   ELEMENT 2: 2nd (follow-up) letter to the GP (& patient consultation)
   ELEMENT 3: Patient & carers' leaflet

6. What do you need for your health to improve? (wider experiences and perceived needs)

Anything not covered? Is there anything that we haven't covered in the interview that you think we should know or think about?

Closing and thanks - check that the participant is still happy for you to use all the information provided and offer the possibility to erase sections of the recording.

For peer review only - http://bmjopen.bmj.com/site/about/guidelines.xhtml

Thank them for their time and contribution.

Interview Topic Guide for Secondary Care Clinicians & GPs

Title of the study: A **C**ommunity and **H**ospital c**A**re **B**undle to improve the medical treatment of c**L**aud**I**cation and critical limb i**S**chaemia. **(**The CHABLIS study)

Welcome and introduction

[Seek consent to continue and to audio-record the interview (if applicable). Participant has to provide written informed consent (see separate form). Let them know that no personal identifiable data will be recorded and a participant number will be allocated to them.]

The main aim of this study is to assess whether a dedicated intervention (a care bundle) can be used in the routine care of patients who present with symptomatic Peripheral Arterial Disease (PAD). The care bundle includes a number of checklists and leaflets for doctors and patients. The intervention is aimed at reducing the risk of major cardiovascular events (i.e. heart attacks and strokes), and preventing amputation, by making sure patients with PAD receive the appropriate treatment and management as recommended by national guidelines. We have called this intervention LEGS (the LEGS bundle), given that the aim is to save legs from amputation and improve vascular care.

ELEMENT 1: In-patient checklist/Outpatient Checklist

ELEMENT 2: 2nd (follow-up) letter to the GP

ELEMENT 3: Patient & carers' leaflet

The interview will explore your experiences/perspectives of delivering the intervention. This will help us to evaluate the ability/feasibility of staff in delivering the intervention, overall impression, validity, usability, acceptability and engagement with the intervention. It will include barriers to engagement or delivery and ideas for improving the intervention.

This is a GUIDE for topics. Further topics will and can be explored if necessary.

The purpose of the study is to find out about staff views and experiences of implementing the LEGS intervention as a part of routine clinical care. This provides us with information on factors that facilitate or hinder implementation and on possible solutions to overcome any identified barriers.

Key objectives of the interview session: i) to identify barriers to and facilitators of each of the key intervention targets; ii) identify issues around delivery, training needs and competencies.

We would like to ask you about what helps and what hinders success in implementing the LEGS intervention as a part of routine clinical care.

- To get started, we would like to ask about your views on existing NICE guidance and factors that might influence implementation of NICE guidance on cardiovascular risk reduction amongst patients with peripheral arterial disease. Please comment on: i)

For peer review only - http://bmjopen.bmj.com/site/about/guidelines.xhtml

prescribing, ii) awareness of NICE guidance by primary and secondary care, iii) adherence by patients (when they have been prescribed their medication), iv) follow-up practices in primary and secondary care.

- What do you think about using the LEGS intervention with this group of patients? Are there any benefits? Or challenges?

Understanding implementation

Aims:
1) To explore what participants think helps or hinders implementation.

2) To explore what participants' identify as potential solutions to implementation barriers.

What helps implementation?

- What factors do you perceive might support implementing the LEGS intervention in routine clinical practice?
- What helps/would help you (and your colleague) to provide the intervention a part of routine clinical care?
- What are your views on the LEGS intervention and the acceptability and feasibility of delivering the intervention?
- Do you feel that you have the knowledge and skills to deliver this intervention?
- What implementation strategies might help? Training?

What prevents or hinders implementation?

- What prevents or hinders success in implementing the LEGS intervention as a part of routine clinical care?
- Are there/or might there be challenges around implementation?
- What would further support you to implement the intervention?

Prompts / Continuation Questions:

- What resources would better equip you to implement the intervention?
- What would you say is the ideal environment to implement the LEGS intervention in?

Implementation solutions

- What solutions would you recommend to address these identified barriers?
- Any other influences on implementation? COVID? Other clinical priorities?
- Any changes needed to the intervention? Ideas for improving the intervention?

(Can you advise us on the 3 most important things you would like to see covered in a patient leaflet for patients with peripheral arterial disease? Can you please advise us

For peer review only - http://bmjopen.bmj.com/site/about/guidelines.xhtml

on the 3 most important things you would like to see covered in a checklist which supports the delivery of peripheral arterial disease care for patients?)

This interview was designed to help us understand the extent to which the CHABLIS intervention has been implemented, and what has helped or hindered you in doing this. Are there any other important points that you would like to discuss before we close the discussion?

Closing and thanks - check that the participant is still happy for you to use all the information provided and offer the possibility to erase sections of the recording.

Thank them for their time and contribution.

Ensure that they have received a PIS and the CF has been handed back for photocopying.

For peer review only - http://bmjopen.bmj.com/site/about/guidelines.xhtml

CFIR Domains and the LEGS bundle	Patients	Secondary care clinicians	GPs
**THE LEGS BUNDLE**			
Element 1: Clinical checklist		X	X
Element 2: Patient and carer education leaflet	X	X	X
Element 3: GP action letter	X	X	X
**CFIR DOMAINS**			
**Domain 1: INTERVENTION CHARACTERISTICS**	**X**	**X**	**X**
**1.1 Intervention source:** Perception about whether the intervention is externally or internally developed.			
The whole bundle		X	X
I sat down with the developers, and they gave me the background		X	
Perception about how the intervention was developed			X
Involving stakeholders in the development process will promote uptake			X
**1.2 Evidence strength and quality:** Perceptions of the quality and validity of evidence supporting the belief that the intervention will have desired outcomes.			
Clinical checklist		X	
Built on existing NICE guidance		X	
The guidance is vague		X	
The guidance is clear, but we don't follow it		X	
**1.3 Relative advantage (or not):** Perception of the advantage of implementing the intervention versus an alternative solution.			
The whole bundle		X	X
Improves care and outcomes		X	
Standardises a minimum quality of care		X	
Allows us to make a difference to patients		X	
Joins up primary and secondary care working			X
Improves awareness of NICE guidance in primary & secondary care		X	X
Improves awareness and understanding of PAD		X	X
Clinical checklist		X	
Builds your relationship with the patient		X	
Improves medication compliance		X	
Patient and carer education leaflet	X	X	X
Patients are empowered by being informed		X	
I don't want any more leaflets	X		
District nurses can answer my questions	X		
A conversation guide for primary care			X
Existing relationships to reinforce the message			X
GP action letter		X	X
A standardised letter with clear information		X	
Useful for non-medical led community vascular clinics			X
Repeat GP action letter is a duplicate, adds to the workload and sets the wrong tone			X
It gives the impression that secondary care do not trust GPs			X

For peer review only

CFIR Domains and the LEGS bundle	Patients	Secondary care clinicians	GPs
**1.4 Adaptability:** The degree to which an intervention can be adapted, tailored, refined, or reinvented to meet local needs.			
Clinical checklist		X	
Adapting to the environment		X	
Fits the in-patient environment neatly		X	
Incorporate into a universal admission checklist		X	
Limited access to blood pressure monitoring or cholesterol tests in out-patient clinic		X	
Promote checklist in primary care to be used alongside secondary care referral letter		X	
Patient and carer education leaflet	X	X	X
Offer personalised care and tailor patient education to individual needs		X	X
A different approach for each type of patient			X
Different information for each patient			X
Existing info most likely to suit those with intermittent claudication			X
Offer realistic targets or the advice rings hollow		X	
Consider co-exiting morbidities			X
Too much information	X		
Change information layout to provide summary at outset		X	
Missing information	X		X
Understanding what causes PAD and preventing further blockages	X		X
Managing the psychological impact of PAD	X		
Maintaining movement and physiotherapy input	X		
Impact on work, social and family life	X		
Acknowledgement of reliance and impact on family	X		
Carer and family support	X		
Managing future risks and when to seek support		X	X
**1.5 Trialability:** The ability to test the intervention on a small scale in the organisation, and to be able to reverse course (undo implementation) if warranted.			
Clinical checklist	X	X	
A quick quality check		X	
Patient and carer education leaflet	X	X	
Leaflet is a resource for referring back	X	X	
**1.6 Complexity:** Perceived difficulty of implementation, reflected by duration, scope, radicalness, disruptiveness, centrality, and intricacy and number of steps required to implement.			
Clinical checklist		X	X
Simplicity		X	
Improvements that make it even simpler		X	
It needs to be a bit more, no and why		X	X
GP action letter	X	X	X
A template for new staff		X	
A time-consuming manual coding process with potential for error			X
Waiting times for primary care/test appointments	X		X
Difficult to assess patients remotely			X

CFIR Domains and the LEGS bundle	Patients	Secondary care clinicians	GPs
Lack of continuity			X
60 calls and 40 letters a day			X
Up to three months to receive hospital letters			X
Patients to hand deliver letter to GP surgery			X
**1.7 Design quality and packaging:** Perceived excellence in how the intervention is bundled, presented, and assembled.			
Clinical checklist		X	
Working across two different systems - Paper and electronic		X	
Paper checklists tend to get missed		X	
Electronic option required		X	
Patient and carer education leaflet	X	X	X
Accessibility	X		
Stored in a safe place	X		
Lost the leaflet	X		
Would be improved if available by email	X		
Presentation & format	X		
Paper leaflet		X	
Larger print has been requested		X	
Language can be hard to understand	X		X
Write for a lower reading age and provide additional information or additional resources for people who are able to access more detailed information			X
Keep it simple, give less information, in different formats and in a way that is understandable			X
Avoid didactic language and reframe the approach			X
There's a lot of 'do nots'			X
Ensure accuracy of information			X
Confusing dietary advice			X
Provide more infographics and pictures	X	X	X
Include diversity in visuals (e.g., pictures of leg ulcers/skin changes in different skin colours)		X	
Add web links			X
Could be supported by existing NHS software like QRX, direct information to patient phones			X
Offer an audio and video version	X	X	X
Patient information to be offered as a group intervention	X	X	
Opportunity for group to be peer-led		X	
GP action letter			X
Same information and same system across primary and secondary care			X
Include information on the discussion around contraindications and side effects of any newly prescribed medication with patient			X
Any contraindications primary care should be aware of, useful for clarification			X
Offered and accepted/declined smoking cessation referral			X
Provide a clear plan for if the situation deteriorates			X
Copy of letter to patient – shared message – reinforcement			X

CFIR Domains and the LEGS bundle	Patients	Secondary care clinicians	GPs
**1.8 Cost:** Costs of the intervention and costs associated with implementing that intervention including investment, supply, and opportunity costs.			X
It is not commissioned or financially viable			X
Primary care time			X
Maintaining equipment			X
**Domain 2: OUTER SETTING**	X	X	X
**2.1 Patient needs and resources:** The extent to which patient needs, as well as barriers and facilitators to meet those needs are known and prioritised.			
The whole bundle	X	X	X
Working as part of a whole team			X
Promoting joined up working across primary and secondary care		X	X
Enabling specialist care from different people			X
Expectations and understanding of each other's roles			X
Timely access back into secondary care if needed			X
Secondary care oversight			X
Accessible referral pathway	X		X
We can't make a referral anyway			X
Secondary care referral thresholds too high			X
You don't fit the criteria so wait until it gets worse			X
Delayed referral to secondary care	X	X	X
Presenting too late		X	
Can I do some of the diagnostics in the community?			X
Time wastage		X	X
A geographical consideration	X		X
Locality of secondary care services	X		X
Transport needs	X		X
Clinical checklist		X	X
Use in primary care to start best medical therapy		X	
Would promote earlier conversations with patients in primary care		X	
Use at point of admission and discharge for in-patients		X	
Adapt for primary care to support referral			X
Patient and carer education leaflet	X	X	X
Patient education provided too late	X	X	
Too far gone to talk about lifestyle modification		X	
Level of functioning & mobility - I can only do what I can do	X		
It is crucial whenever it happens		X	
Introduced sooner in primary care as part of a PAD prevention programme			X
Informing patients to reduce the chance of presenting late/in crisis			X
Managing patient expectations			X
**2.2 Cosmopolitanism:** The degree to which an organisation is networked with other external organisations.			
The whole bundle		X	
Shared primary & secondary care working		X	
Patient and carer education leaflet			X

CFIR Domains and the LEGS bundle	Patients	Secondary care clinicians	GPs
Copy of leaflet to primary care to reiterate message			X
GP action letter		X	
Clear open communication across community and hospital services		X	
Joined up clinical record systems		X	
We don't sing off the same hymn sheet		X	
**2.3 External policies and incentives:** A broad construct that includes external strategies to spread interventions including policy and regulations (governmental or other central entity), external mandates, recommendations and guidelines, pay-for-performance, collaboratives, and public or benchmark reporting.			
The whole bundle		X	
Additional funding		X	
**Domain 3: INNER SETTING**	X	X	X
**3.1 Structural characteristics:** The social architecture, age, maturity, and size of an organisation.			
The whole bundle		X	X
Impact of staff shortages		X	
Reduced staffing due to covid		X	
Involving the multi-disciplinary team			X
GP action letter		X	X
Time delay between writing and getting to primary care		X	X
Patient and carer education leaflet	X	X	X
Being able to follow the advice	X	X	
Difficulty ordering repeat medication	X		
Relying on others for food shopping	X		
Availability of supervised exercise		X	
It's easy to bombard somebody with information			X
**3.2 Networks and communications:** The nature and quality of webs of social networks and the nature and quality of formal and informal communications within an organisation.			
The whole bundle	X	X	X
A team approach		X	
Building relationships			X
Clear communication			X
The patient is in the middle			X
Involve all multi-disciplinary professionals (including pharmacists, nurses, physios)		X	
Access to the vascular team or not	X		X
Advice and information	X		X
**3.3 Culture:** Norms, values, and basic assumptions of a given organisation.			
The whole bundle		X	
Target patients earlier		X	
The gap in no man's land		X	
**3.4 Implementation climate - tension for change:** The degree to which stakeholders perceive the current situation as intolerable or needing change.			
The whole bundle		X	X
Primary care needs to do more sooner		X	
Secondary care referral threshold is too high			X
**3.5 Implementation climate - Compatibility:** The degree of tangible fit between meaning and values attached to the intervention by involved individuals, how those align with individuals' own norms, values, and			

CFIR Domains and the LEGS bundle	Patients	Secondary care clinicians	GPs
perceived risks and needs, and how the intervention fits with existing workflows and systems.			
The whole bundle			X
Existing systems in place for primary care medication management and monitoring			X
Provide 28 days medication to allow for delay in seeing GP			X
Preventative medicine and health promotion is core to primary care practice			X
Ability to offer smoking cessation support			X
Local variation in set-up of smoking cessation, dietary advice, exercise therapy			X
No formal set-up in primary care to pick it up			X
GP relationship with patients			
Not commissioned			
Secondary care to initiate smoking cessation referral			X
National protocol for weight reduction			X
Existing relationships with Diabetes nurses/clinics			X
**3.6 Implementation climate - Relative priority:** Individuals' shared perception of the importance of the implementation within the organisation.			
Clinical checklist		X	
Take the opportunity		X	
It shouldn't be seen as an extra thing		X	
A threat to autonomy		X	
Checklists are tick-box exercises rather than safety mechanisms		X	
**3.7 Implementation climate - Learning climate**			
The whole bundle			X
Two-way learning			X
Secondary care can learn from this too			X
Holistic picture of medication profile, side effects, competing medication			X
Teams are not static so require ongoing access to training		X	X
**3.8 Readiness for implementation – leadership engagement:** Commitment, involvement, and accountability of leaders and managers with the implementation.			
The whole package		X	
Provide a named leader		X	
External oversight is preferable		X	
**3.9 Readiness for implementation – available resources:** The level of resources dedicated for implementation and on-going operations including money, training, education, physical space, and time.			
Clinical checklist		X	X
Checklists are quick		X	
Managing it alongside the burden of work		X	
The assumption that the GP will sort it – putting it back into GP land		X	X
Lack of access to testing equipment in community			X
Limited community workforce (i.e., reduced number of district nurses) capacity for conducting ABPIs			X
Secondary care are gatekeepers for ABPIs and duplexes			X

CFIR Domains and the LEGS bundle	Patients	Secondary care clinicians	GPs
Waiting for secondary care to make diagnosis			X
Patient and carer education leaflet		X	
Time to talk requires extra staff		X	
GP action letter		X	X
Time burden and impact of dictating long clinic letters		X	
Joining it up - We've done our bit and now pass it on to the GP			X
Routine practice or adding to the workload			X
Feels top down and with didactic language			X
Keep the GP onside and change phraseology			X
**3.10 Readiness for implementation - access to information and knowledge:** Ease of access to digestible information and knowledge about the intervention and how to incorporate it into work tasks.			
The whole bundle		X	
Training on the purpose of the intervention		X	
Written information for those unable to attend training		X	
Prioritise training and supervision for junior staff – the people who do the day-to-day work		X	
Consider involving medical students		X	
Highlight the link between PAD and cardiovascular death		X	
Education on managing CLTI and Non-CLTI patients in primary care		X	
Knowing the risk factors for CLTI and how to make a referral to secondary care		X	
Taking an ABPI in primary care		X	X
Support the triage for secondary care			X
We're not commissioned to do it			X
Visibility of vascular teams at primary care conferences		X	
Learning on the job		X	X
Informal training to colleagues i.e., when seeing a medical outlier		X	
Use available supervision/debriefing opportunities			X
Webinar-ed out		X	
Training delivered by vascular team to primary care			X
Collaborative working and building relationships			X
We don't ever see secondary care specialists			X
Involve all primary care staff and not just GPs			X
Learning gap in exercise and lifestyle modification			X
**DOMAIN 4: CHARACTERISTICS OF INDIVIDUALS**			
The whole bundle	X	X	X
Taking on the advice	X	X	X
Familiarity and understanding of PAD		X	X
**4.1 Knowledge and beliefs about the intervention**			
Clinical checklist		X	X
Prioritising the checklist (or not)		X	
Previous experience of using checklists and identifying benefits		X	
Checklists are a useful memory prompt		X	
Patient and carer education leaflet	X		

CFIR Domains and the LEGS bundle	Patients	Secondary care clinicians	GPs
Targets that are impossible to reach	X		
**4.2 Self-efficacy**			
The whole bundle		X	
Confidence to implement guidelines		X	
**4.3 Individual stage of change**			
Clinical checklist		X	
Put myself forward to find out more		X	
Patient and carer education leaflet	X	X	X
If I was younger and I had more get-up-and-go	X		
It's down to willpower at the end of the day (I like smoking, I like drinking, I like the telly) – patient engagement & stage of change – resistance from patients	X	X	X
The only thing left is something nice to eat	X		
Resistance from patients		X	
Secondary care doctors tell people to do things			X
Everybody knows they need to stop smoking			X
**4.4 Individual identification with organisation:** how individuals perceive the organization and their relationship and degree of commitment with that organisation.			
The whole bundle		X	
Joined up working with primary care		X	
Patient and carer education leaflet	X	X	X
The patient – health professional relationship	X	X	
It takes time to build the relationship and have the conversation		X	
Clear open communication	X		
Frank conversations for some	X	X	
Be honest and explain the risks		X	
Information on the benefits versus taking pleasure away		X	
Striking a balance to optimise people's quality of life. You can't take everything away.		X	
Patient preferences and shared decision making		X	
Online tools to support the clinical conversation		X	
Language and approach used to engage people			X
Consider use of motivational interviewing techniques in secondary care			X
GP action letter			X
Partnership working			X
Avoid the extra step and order the test in secondary care			X
**4.5 Other personal attributes:** other personal traits such as tolerance of ambiguity, intellectual ability, motivation, values, competence, capacity, and learning style.			
Clinical checklist		X	
Team members who prioritise PAD		X	
Patient and carer education leaflet	X		
Living with other health conditions alongside (arthritis, diabetes)	X		
Managing the pain	X		
Medication side-effects	X		

For peer review only

CFIR Domains and the LEGS bundle	Patients	Secondary care clinicians	GPs
**DOMAIN 5: PROCESS**			
**5.1 Engaging:** Attracting and involving appropriate individuals in the implementation and use of the intervention through a combined strategy of social marketing, education, role modelling, training, and other similar activities.			
The whole bundle		X	
Championing the cause		X	
Doing the bare minimum and going home on time		X	
Using research to demonstrate a difference		X	
Involving stakeholders at an early stage		X	
Clinical checklist		X	
Posters in clinic to remind staff		X	
**5.2 Executing:** Carrying out or accomplishing the implementation according to plan.			
Patient and carer education leaflet	X		
A direct contact number for the vascular team	X		
Primary care capacity	X		
My GP and practice nurses keep an eye on me	X		
Sometimes it can take weeks to get them to answer a query over the phone	X		
GP action letter	X		
It can take weeks to get information on their system	X		
It's lots of different clinics and different people involved in your healthcare (whole person care)	X		

CFIR matrix and distribution of descriptive themes

For peer review only

Full coding framework

Theme 1: The potential to make a difference

1.1 An opportunity to meet patient need

1.1.1 Improves care and outcomes
1.1.1.1 Standardises a minimum quality of care
1.1.2 Improves awareness and understanding of PAD
1.1.3 Improves awareness of existing NICE guidance
1.1.3.1 Provides a tool to support implementation of NICE guidance
1.1.3.1.1 The guidance is clear but we don't always follow it
1.1.4 Builds your relationship with the patient
1.1.4.1 Empowers patients by being informed

1.2 Shared primary and secondary care working

1.2.1 Working as a part of a whole team
1.2.1.1 Allows for secondary care oversight
1.2.1.2 An opportunity for collaborative working
1.2.1.2.1 Managing expectations and understanding each other's roles
1.2.1.2.2 We don't sing off the same hymn sheet
1.2.1.2.2.1 The patient is in the middle
1.2.1.3 Multidisciplinary working
1.2.1.3.1 Enabling specialist care from different people
1.2.1.4 Promoting continuity of care
1.2.1.4.1 There's lots of different clinics and different people involved in your care
1.2.1.4.2 Timely access back into secondary care if needed
1.2.2 Joining up and building relationships across the team
1.2.2.1 We don't ever see secondary care specialists
1.2.2.2 Access to the vascular team (or not)
1.2.2.2.1 A direct contact number for the vascular team
1.2.2.3 We've done our bit and now it's over to the GP
1.2.3 Clear open communication across community and hospital services
1.2.4 Working together and learning together
1.2.4.1 An opportunity for two-way learning
1.2.4.1.1 Secondary care can learn from this too
1.2.4.1.1.1 Support for screening questions on prescribing and contraindications
1.2.4.1.1.1.1 Holistic picture of medication profile, side effects, competing medication
1.2.4.1.1.2 Familiarity (or not) with reviewing cholesterol and HbA1c's – it's a GP thing

Theme 2: A solution for addressing the gap in no man's land

2.1 We need to target patients earlier

2.1.1 Patients presenting too late
2.1.1.1 Delayed referral to secondary care
2.1.1.1.1 We can't make a referral anyway
2.1.1.1.1.1 Secondary care referral thresholds are too high
2.1.1.1.1.1.1 Patients don't meet the criteria so have to wait until it gets worse
2.1.1.2 Waiting for secondary care to make the diagnosis
2.1.1.2.1 Support general practice to do some of the diagnostics
2.1.1.2.1.1 Lack of access to testing equipment in the community
2.1.1.2.1.2 Secondary care are gatekeepers for ABPIs and duplexes

2.2 Primary care needs to do more sooner

2.2.1 Adapt for use in general practice as part of a PAD prevention programme

For peer review only

2.2.1.1 Would promote earlier conversations with patients
2.2.1.2 Useful for managing patient expectations
2.2.1.3 Would support primary care to start best medical therapy
2.2.1.4 Could be used to provide additional information for referral to vascular team
2.2.1.4.1 Would support triage in secondary care
2.2.2 Use as an ongoing conversation guide
2.2.2.1 Existing relationships with patients will help to reinforce the message
2.2.2.2 Informing patients reduces the chance of presenting late or in crisis

Theme 3: Prioritising and making it happen

3.1 Prioritising alongside the burden of work
3.1.1 It shouldn't be seen as an extra thing
3.1.1.1 Previous experience of using intervention/checklists
3.1.1.2 Checklists are a quick quality check
3.1.1.3 Checklists are a useful memory prompt
3.1.1.4 Checklists are a threat to autonomy
3.1.1.5 Checklists are tick-box exercises rather than safety mechanisms
3.1.2 Confidence to implement guidelines
3.1.2.1 It's essentially what I always do
3.1.3 It takes time to build the relationship and have the conversation
3.1.3.1 Time to talk requires extra staff
3.1.3.2 Time burden of dictating long clinic letters
3.1.3.3 Limited access to blood patient monitoring and cholesterol tests in out-patient clinics
3.1.4 Impact of staff shortages
3.1.4.1 Reduced staffing due to covid
3.1.4.2 Reduced district nurse staffing for conducting ABPIs
3.1.5 Primary care is not an infinite resource
3.1.5.1 Long waiting times to be seen or to get tests
3.1.5.1.1 Sometimes it can take weeks to get them to answer a query over the phone
3.1.5.2 Sixty calls and forty letters a day
3.1.5.3 Patients don't always see the same GP
3.1.5.4 Championing the cause or doing the bare minimum and going home on time
3.1.5.4.1 Using research to demonstrate a difference

3.2 Working across primary and secondary care systems
3.2.1 Preventative medicine and health promotion is core to primary care practice
3.2.1.1 Existing systems in place for primary care medication management and monitoring
3.2.1.1.1 It can take weeks to get information on the system
3.2.1.1.2 Time delay between writing and receiving clinic letters
3.2.1.1.3 It can take up to three months to receive hospital letters
3.2.1.1.3.1 Provide 28 days medication to allow for delay in seeing GP
3.2.1.1.3.2 Patient difficulty ordering repeat medication
3.2.1.1.4 Avoid the extra step and order the test in secondary care
3.2.1.1.5 Secondary care to initiate smoking cessation referral
3.2.1.1.5.1 Local variation in community set-up of smoking cessation, dietary advice, exercise therapy support
3.2.1.1.5.1.1 No formal set-up in primary care to pick it up
3.2.1.1.5.1.2 Link with national protocol for weight reduction
3.2.1.1.6 It's not commissioned or financially viable
3.2.2 Provide a copy of the clinic/GP letter to the patient to reinforce shared messaging
3.2.2.1 Patients to hand deliver letters to general practice
3.2.3 Repeat GP action letter is a duplicate, adds to the workload, and sets the wrong tone
3.2.3.1 It gives the impression that secondary care do not trust GPs

3.3 Adapting the intervention to the environment
3.3.1 Fits the in-patient environment neatly
3.3.1.1 Incorporate into a universal admission/discharge bundle
3.3.1.1.1 Use at point of admission and discharge for in-patients
3.3.2 Use the intervention to support the outpatient clinic consultation
3.3.2.1 A record of the consultation conversation
3.3.2.2 A resource for referring back
3.3.3 Complexity in assessing patients remotely in the community

3.4 Training to support implementation
3.4.1 Teams are not static so require ongoing access to training
3.4.1.1 Prioritise junior staff – the people who do the day-to-day work
3.4.1.1.1 Consider including medical students
3.4.1.2 Written information for those unable to attend training
3.4.1.3 Webinar-ed out
3.4.2 Training delivered by vascular team to primary care
3.4.2.1 Involve all primary care staff and not just GPs
3.4.2.1.1 Highlight the link between PAD and cardiovascular death
3.4.2.1.2 Provide education on managing CLTI and non-CLTI patients in primary care
3.4.2.1.3 Taking an ABPI in primary care
3.4.2.1.4 Learning gaps in exercise and lifestyle modification
3.4.2.2 Visibility of vascular teams at primary care conferences
3.4.2.3 Use available supervision and de-briefing opportunities

Theme 4: Personalised information and supportive conversations for taking on the advice

4.1 Offer personalised care and tailor patient information to individual needs
4.1.1 Existing information does not suit all
4.1.1.1 Currently most likely to suit those with IC
4.1.1.2 Targets are impossible to reach
4.1.1.2.1 If I was younger and had more get-up-and-go
4.1.1.2.2 Availability of supervised exercise
4.1.1.2.3 Relying on others for food shopping
4.1.2 A different approach for each patient
4.1.3 Offer realistic targets or the advice rings hollow
4.1.4 Patient education leaflet is provided too late
4.1.4.1 Too far gone to talk about lifestyle modification
4.1.4.1.1 I can only do what I can do
4.1.4.2 Consider co-existing morbidities
4.1.4.3 Offer advice on moderating activity levels and pain management
4.1.4.4 Discuss concerns about medication side-effects

4.2 Everyone knows they need to stop smoking
4.2.1 Acknowledge and support patient preference and promote shared decision making
4.2.1.1 It's easy to bombard someone with information
4.2.1.2 Secondary care doctors tell people to do things
4.2.1.2.1 Consider the language and approach to engage patients
4.2.1.2.2 Be honest and explain the risks
4.2.1.2.2.1 Use available online tools to support the conversation
4.2.1.2.3 Use motivational interviewing techniques
4.2.1.2.4 Frank conversations for some
4.2.2 It's down to willpower at the end of the day
4.2.2.1 I like smoking, I like drinking, I like the telly

For peer review only

4.2.2.2 The only thing left is something nice to eat
4.2.2.2.1 Provide information on the benefits versus taking pleasure away
4.2.2.2.2 Strike a balance to optimise people's quality of life
4.2.2.2.2.1 You can't take everything away
4.2.3 Patient education leaflets are not the best way for everyone
4.2.3.1 I don't want any more leaflets
4.2.3.1.1 District nurses can answer my questions

For peer review only

COREQ checklist

Item number	Descriptor	Reported on page number
1	Interviewer/facilitator: Which author/s conducted the interview or focus group?	8
2	Credentials: What were the researcher's credentials? E.g. PhD, MD	8
3	Occupation: What was their occupation at the time of the study?	8
4	Gender: Was the researcher male or female?	
5	Experience and training: What experience or training did the researcher have?	8
6	Relationship established: Was a relationship established prior to study commencement?	N/A
7	Participant knowledge of the interviewer: What did the participants know about the researcher? E.g. personal goals, reasons for doing the research	N/A
8	Interviewer characteristics: What characteristics were reported about the interviewer/facilitator? E.g. bias, assumptions, reasons and interests in the research topic	8
9	Methodological orientation and theory: What methodological orientation was stated to underpin the study? E.g. grounded theory	6-7
10	Sampling: How were participants selected? E.g. purposive, theoretical	7
11	Method of approach: How were participants approached? E.g. face-to-face, telephone, email	7
12	Sample size: How many participants were in the study?	9
13	Non-participation: How many people refused to participate or dropped out? Reasons?	9-10
14	Setting of data collection: Where was the data collected? E.g. clinic, work place	8
15	Presence of non-participants: Was anyone else present besides the participants and researchers?	N/A
16	Description of sample: What are the important characteristics of the sample? E.g. demographic data, date	9
17	Interview guide: Were questions, prompts, guides provided by the authors? Was it pilot tested?	7-8, Data supplement
18	Repeat interviews: Were repeat interviews carried out? If yes, how many?	N/A
19	Audio/visual recording: Did the research use audio or visual recording to collect the data?	8
20	Field notes: Were field notes made during and/or after the interview or focus group?	8
21	Duration: What was the duration of the interviews or focus group?	8
22	Data saturation: Was data saturation discussed?	N/A
23	Transcripts returned: Were transcripts returned to participants for comment and/or correction?	N/A
24	Number of data coders: How many data coders coded the data?	8-9
25	Description of the coding tree: Did authors provide a description of the coding tree?	10-11, data supplement
26	Derivation of themes: Were themes identified in advance or derived from the data?	8
27	Software: What software was used to manage the data?	8
28	Participant checking: Did participants provide feedback on the findings?	N/A

For peer review only

29	Quotations presented: Were participant quotations presented to illustrate the themes/findings? Was each quotation identified? E.g. participant number	11-16
30	Data and findings consistent: Was there consistency between the data presented and the findings?	11-16<br>All findings illustrated with quotes
31	Clarity of major themes: Were major themes clearly presented in the findings?	11-16
32	Clarity of minor themes: Is there a description of diverse cases or discussion of minor themes?	11-16

Reference: Tong A, Sainsbury P, Craig J. Consolidated criteria for reporting qualitative research (COREQ): a 32-item checklist for interviews and focus groups. International Journal for Quality in Health Care. 2007; 19(6): 349-357.

For peer review only

BMJ Open

Factors that influence the feasibility and implementation of a complex intervention to improve the treatment of Peripheral Arterial Disease in primary and secondary care: a qualitative exploration of patient and provider perspectives

Journal:	*BMJ Open*
Manuscript ID	bmjopen-2022-066883.R1
Article Type:	Original research
Date Submitted by the Author:	13-Oct-2022
Complete List of Authors:	Le Boutillier, Clair; King's College London<br>Saratzis, Athanasios; University of Leicester<br>Saha, Prakash; Guy's and St Thomas' NHS Foundation Trust<br>Benson, Ruth; University of Birmingham<br>Bridgwood, Bernadeta; University of Leicester<br>Watson, Emma; University of Leicester<br>Lawrence, Vanessa; King's College London, Health Services & Population Research Department
**Primary Subject Heading**:	Cardiovascular medicine
Secondary Subject Heading:	Qualitative research, Patient-centred medicine, Cardiovascular medicine
Keywords:	QUALITATIVE RESEARCH, VASCULAR MEDICINE, Change management < HEALTH SERVICES ADMINISTRATION & MANAGEMENT

SCHOLARONE™
Manuscripts

I, the Submitting Author has the right to grant and does grant on behalf of all authors of the Work (as defined in the below author licence), an exclusive licence and/or a non-exclusive licence for contributions from authors who are: i) UK Crown employees; ii) where BMJ has agreed a CC-BY licence shall apply, and/or iii) in accordance with the terms applicable for US Federal Government officers or employees acting as part of their official duties; on a worldwide, perpetual, irrevocable, royalty-free basis to BMJ Publishing Group Ltd ("BMJ") its licensees and where the relevant Journal is co-owned by BMJ to the co-owners of the Journal, to publish the Work in this journal and any other BMJ products and to exploit all rights, as set out in our licence.

The Submitting Author accepts and understands that any supply made under these terms is made by BMJ to the Submitting Author unless you are acting as an employee on behalf of your employer or a postgraduate student of an affiliated institution which is paying any applicable article publishing charge ("APC") for Open Access articles. Where the Submitting Author wishes to make the Work available on an Open Access basis (and intends to pay the relevant APC), the terms of reuse of such Open Access shall be governed by a Creative Commons licence – details of these licences and which Creative Commons licence will apply to this Work are set out in our licence referred to above.

Other than as permitted in any relevant BMJ Author's Self Archiving Policies, I confirm this Work has not been accepted for publication elsewhere, is not being considered for publication elsewhere and does not duplicate material already published. I confirm all authors consent to publication of this Work and authorise the granting of this licence.

For peer review only

Title: **Factors that influence the feasibility and implementation of a complex intervention to improve the treatment of Peripheral Arterial Disease in primary and secondary care: a qualitative exploration of patient and provider perspectives**

Authors: Clair Le Boutillier[1,2], Athanasios Saratzis[3], Prakash Saha[4], Ruth Benson[5], Bernadeta Bridgwood[6], Emma Watson[3,6], Vanessa Lawrence*[1]

[1] Institute of Psychiatry, Psychology & Neuroscience, King's College London, UK

[2] Florence Nightingale Faculty of Nursing, Midwifery & Palliative Care, King's College London, UK

[3] University of Leicester, UK

[4]Guy's and St Thomas' NHS Foundation Trust, UK

[5]University of Birmingham, UK

[6]NIHR Cardiovascular Sciences, University of Leicester, Leicester, UK.

* Corresponding authors. Email: vanessa.c.lawrence@kcl.ac.uk; clair.le_boutillier@kcl.ac.uk

Division of Methodologies, Florence Nightingale Faculty of Nursing, Midwifery & Palliative Care, King's College London, James Clerk Maxwell Building, 57 Waterloo Road, London SE1 8WA. Orcid iD: 0000-0003-3665-0315

Funding

The study was funded by the National Institute for Health Research (NIHR) – Research for Patient Benefit (RfPB) Programme (Reference: NIHR202008).

Category

Original article

For peer review only - http://bmjopen.bmj.com/site/about/guidelines.xhtml

Abstract

Objectives: Our aim was to examine the feasibility and implementation of a complex intervention to improve the care of patients with Peripheral Arterial Disease (the LEGS intervention) from the perspective of patients, general practitioners and secondary care clinicians.

Design: A qualitative study involving semi-structured individual interviews with patients and providers to gain an understanding of the feasibility of the LEGS intervention as well the barriers and facilitators to implementation in secondary and primary care.

Setting: Primary and secondary care settings across two NHS Trusts.

Participants: Twenty-five semi-structured telephone interviews were conducted with i) patients who had received the intervention (n=11), ii) secondary care clinicians responsible for delivering the intervention (n=8), and iii) general practitioners (n=6).

Analysis Data were initially analysed using inductive descriptive thematic analysis. The consolidated framework for implementation research was then used as a matrix to explore patterns in the data and to map connections between the three participant groups. Lastly, interpretive analysis allowed for refining, and a final coding frame was developed.

Results: Four overarching themes were identified: i) The potential to make a difference, ii) A solution to address the gap in no man's land, iii), Prioritising and making it happen and iv) Personalised information and supportive conversations for taking on the advice. The impetus for prioritising and delivering the intervention was further driven by its flexibility and adaptability to be tailored to the individual and to the environment.

Conclusions: The LEGS intervention can be tailored for use at early and late stages of Peripheral Arterial Disease, provides an opportunity to meet patient needs and can be used to promote shared working across the primary-secondary care interface.

Strengths and limitations

For peer review only - http://bmjopen.bmj.com/site/about/guidelines.xhtml

- A strength of this study is the focus on rigor and triangulation of participant perspectives.
- We used implementation theory to guide analysis and to provide a layered coding approach, to ensure that intervention and implementation recommendations are novel, grounded in the data, and useful.
- While the anonymity of telephone use can allow participants to disclose sensitive information, telephone interviews have received criticism for compromising interviewer/participant rapport and interaction, and for limiting contextual data due to the absence of face-to-face contact and visual cues. However, this method of data collection is convenient, in that it is flexible (in terms of time and location), accessible (i.e., remote research conducted during the COVID-19 pandemic), and allows for a wide reach (e.g., accessing a diverse population from both urban and rural areas).
- Although the sample size for this study may seem small, the volume of interview data we collected is unique for this patient population. Living with the symptoms of Peripheral Arterial Disease is challenging, and patients also experience barriers to accessing healthcare. Engaging in research alongside managing these specific health care and health needs can be burdensome.

List of abbreviations

PAD	Peripheral Arterial Disease
IC	Intermittent Claudication
CLTI	Chronic Limb Threatening Ischaemia
GP	General Practitioner
CHABLIS	Community and Hospital cAre Bundle to improve the medical treatment of cLaudIcation and critical limb iSchaemia
LEGS	LEaflet Gp letter Structured checklist
UHL	University Hospitals of Leicester NHS Trust
GSTT	Guy's and St Thomas' NHS Foundation Trust
CFIR	The consolidated framework for implementation research
NHS	National Health Service**Background**

For peer review only - http://bmjopen.bmj.com/site/about/guidelines.xhtml

Peripheral Arterial Disease (PAD) affects a fifth of people over the age of sixty in the United Kingdom. This prevalence is also expected to rise due to sedentary lifestyles, poor dietary choices, a longer life-expectancy, and rising numbers of those with diabetes (1, 2). Patients with PAD who develop symptoms present either with Intermittent Claudication (IC) or in some cases, Chronic Limb Threatening Ischaemia (CLTI) where the reduction in blood flow is so severe that it causes pain at rest, ulceration, or gangrene (3). Patients with IC are more likely to suffer a cardiovascular event compared to age-matched individuals, and the one-year risk of limb amputation for those with CLTI is 30%. More than half of those diagnosed with symptomatic PAD are expected to die, have an amputation or a major cardiovascular event within five years (4-7).

The treatment of these high-risk individuals is shared between primary and secondary care. While established national and international guidance (8) outlines how PAD risk-factors should be addressed to prevent cardiac events and amputations, a study conducted by our group found that only a tenth of those diagnosed with PAD were prescribed appropriate preventive medication, and almost none received structured lifestyle or dietary support (9). As a result, and following patient and public engagement (that is, interviews and workshops with patients and a national consultation with primary and secondary care clinicians) and a review of the existing evidence, a complex intervention in the form of a care bundle was developed to support patients, General Practitioners (GPs), and secondary care clinicians to actively manage cardiovascular risk-factors, and to improve implementation of existing guidance when treating patients with PAD. The care bundle, called the LEGS intervention (Leaflet, Gp letter, Structured checklist) has three elements: i) a one-page clinical checklist for the ward and a separate one-page clinical checklist for the secondary care clinic, ii) a patient and carer education leaflet (developed with patients), and iii) a GP action letter, that can be sent to GPs at regular intervals to inform them of the PAD diagnosis and corresponding practice guidance.

For peer review only - http://bmjopen.bmj.com/site/about/guidelines.xhtml

The LEGS intervention and full study protocol are available online (isrctn.com/ISRCTN13202085).

This qualitative study was embedded within the Community and Hospital cAre Bundle to improve the medical treatment of cLaudIcation and critical limb iSchaemia (CHABLIS) study. CHABLIS is a multicentre prospective mixed-methods cohort study that aims to determine the feasibility and effectiveness of the LEGS intervention. The aim of the qualitative study was to gain an understanding of the feasibility and implementation of the LEGS intervention, by exploring patient and provider perspectives on receiving and delivering the intervention (10). Guided by the MRC framework for developing and evaluating complex interventions, the objectives of the qualitative study were: 1) to explore patient perspectives on receiving the LEGS intervention, 2) to gather secondary care clinician views on feasibility, and to understand factors that influence the success of implementation, and 3) to identify barriers and facilitators that influence the success of future implementation from the GP perspective.

METHODS

Study design

The study used qualitative methods to provide insights into the feasibility and acceptability of the intervention as well as the complexities of implementation (10). Semi-structured individual interviews were conducted with patients and providers to gain an understanding of the barriers and facilitators to implementation in secondary and primary care. The research was reviewed and approved by the NHS Wales Research Ethics Committee (REC), reference number20/WA/0319 and the NHS Health Research Authority (HRA). The consolidated criteria for reporting qualitative research (COREQ) guideline was used to ensure quality when reporting the research (11).

For peer review only - http://bmjopen.bmj.com/site/about/guidelines.xhtml

To meet objective 1, semi-structured individual interviews were conducted with patients with symptomatic PAD (i.e., IC, ischaemic rest pain, or CLTI), who had received the intervention (either in an outpatient clinic or as a part of inpatient treatment). To meet objective 2, semi-structured individual interviews were conducted with secondary care clinicians who were responsible for delivering (that is, delivering or intending to deliver) the intervention, including junior doctors, vascular nurses, and vascular surgeons. Staff and patients were recruited from two implementation sites: University Hospitals of Leicester NHS Trust (UHL) and Guy's and St Thomas' NHS Foundation Trust (GSTT). To meet objective 3, semi-structured individual interviews were conducted with GPs to gain an understanding of the barriers and facilitators to future implementation in primary care and across the NHS. GPs were not involved in delivering the study intervention and were asked to report on perceived factors that might influence implementation beyond the context of the study.

Participants were selected purposively (both those who were receiving or responsible for delivering the LEGS intervention) on the basis that they are the target adopters of behaviours that influence the success of future implementation and/or could offer a particular perspective on implementation. All patients who had received the intervention and who gave consent for the researcher to make contact about the qualitative study were approached for participation (n=26). All secondary care clinicians responsible for delivering the intervention (n=13) were invited to take part. Secondary care clinicians were invited to participate following an in-person meeting with the local principal investigator at each site. A participant information sheet that outlined the study was provided to all potential participants with time to consider whether or not they would like to take part. GP participants were recruited from Clinical Commissioning Groups linked to the two secondary care implementation sites, chosen to enhance joined up working and to provide a local perspective on implementation across community and hospital settings. Potential GP participants (n=7) were first approached by the local Clinical Research Network research delivery manager or local contacts, and subsequently recruited by the lead

For peer review only

author via telephone or email. Written or verbal informed consent was obtained from each participant after a full explanation and information leaflet was given and time allowed for consideration. Participants were not reimbursed for participating in the research. The right of each participant to refuse to participate without giving reasons was respected. All participants were free to withdraw from the study at any time without giving reasons and without prejudicing further treatment or employment.

Data collection

Interviews used flexible open-ended questions for early data collection to gather a rich and detailed understanding of participants' perspectives (included in Online Data Supplement 1). The patient and provider interview schedules were revised iteratively in response to the priorities and concerns of participantsand gathered information on factors that facilitate or hinder implementation and on possible solutions to overcome any identified barriers. Interviews were conducted by telephone by the lead author (a qualitative research fellow), lasted around 45 minutes, were audio-recorded and transcribed verbatim. Researcher reflexive notes were kept after each interview to consider the interaction with the participant, and to detail initial thoughts. Interviews were conducted between September and November 2021.

Data Analysis

Data from the three participant groups (patients, secondary care clinicians and GPs) were first analysed separately using inductive thematic analysis, where analytical concepts and perspectives are generated from the data in a deliberate and systematic way (12). Data analysis began with repeated re-reading of individual transcripts and re-listening of sound files for data immersion. This was followed by line-by-line open coding, where extracts were coded under one or several descriptive themes, that is organising the data according to semantic

For peer review only - http://bmjopen.bmj.com/site/about/guidelines.xhtml

content, to capture their meaning and reflect the content of the data. Each theme was refined, and where data allowed, further sub-themes were developed. The Consolidated Framework for Implementation Research (CFIR) was then used as a matrix to organise the early themes, to explore patterns and relationships in the data, and to map connections between the themes and the three participant groups (13). Lastly, interpretive analysis allowed for the refining of the specifics of themes and thematic patterns, and a final coding frame for patients and providers was developed. Refinements to the specifics of themes, and thematic patterns continued until a useful and meaningful analysis was achieved (14).

Data collection occurred concurrently with data analysis; NVivo QSR International qualitative analysis software (version 12) was used to manage the data. (15). The lead author (CL) directed and conducted the analysis. Coding by a second analyst (VL) was undertaken to provide an opportunity to reflect on the coding approach, and to enhance the interpretive depth of the data (14). This involved an independent review of the data, the themes, and the CFIR mapping/tabulation. The overarching coding framework was developed following discussion with both analysts.

Patient and public involvement

The LEGS intervention and CHABLIS mixed-methods study design were developed in collaboration with a stakeholder PPI group that included patients, family members and carers, and clinicians working in primary and secondary care. (16).

RESULTS

Participants

A total of 25 individual interviews were conducted with i) patients who had received the intervention (n=11), ii) secondary care clinicians responsible for delivering the intervention (n=8), and iii) GPs (n=6). Two of the eight secondary care clinicians were actively delivering the intervention (in an in-patient setting), and all GPs provided insights on future

For peer review only

For peer review only - http://bmjopen.bmj.com/site/about/guidelines.xhtml

implementation. Six patients had received the intervention as a part of in-patient care and five patients provided perspectives on receiving the intervention from out-patient vascular, ulcer, or specialised CLTI clinics. Patient participants spoke about living with multiple conditions including diabetes, stroke, and amputation and receiving care from primary care (GPs, district nurses, diabetic nurses) secondary care (vascular teams), social care (carers), and family. Three patients were >70 years old but all patients were retired or unemployed and spoke about the impact of PAD on quality of life including managing pain, slow wound healing, poor sleep, reduced daily functioning, and limited mobility. Patient characteristics are shown in Table 1 and provider characteristics are detailed Table 2.

Table 1: Patient participant characteristics

**Patient characteristics** n (%)	Patients n=11
**Age**	
41-50 years	1 (9.1)
51-60 years	0 (0.00)
61-70 years	2 (18.2)
>70 years	8 (72.7)
**Gender**	
Male	8 (72.7)
Female	3 (27.3)
**Ethnicity**	
White British	9 (81.8)
White Irish	1 (9.1)
Black/Black British-Caribbean	1 (9.1)
**Marital status**	
Single	2 (18.2)
Married	7 (63.6)
Widowed	2 (18.2)
**Time since diagnosis**	
>1 year	3 (27.3)
1-4 years	4 (36.35)
5-9 years	0 (0.00)
10 years +	4 (36.35)
**No. of surgical interventions**	
None	1
Angioplasty	8
Bypass graft	4
Amputation	2

For peer review only - http://bmjopen.bmj.com/site/about/guidelines.xhtml

Table 2: **Provider participant characteristics**

**Provider characteristics** n (%)	Hospital staff n=8	GPs n=6
**Age**		
21-30 years	3 (37.5)	1 (16.7)
31-40 years	5 (62.5)	3 (50.0)
41-50 years	0 (00.0)	2 (33.3)
**Gender**		
Male	4 (50.0)	4 (66.7)
Female	4 (50.0)	2 (33.3)
**Ethnicity**		
White British	2(25.0)	3 (50.0)
White Other	1 (12.5)	1 (16.7)
Asian/Asian British-Indian	2 (25.0)	2 (33.3)
Mixed. White & Asian	1 (12.5)	0 (00.0)
Chinese	1 (12.5)	0 (00.0)
Other	1 (12.5)	0 (00.0)
**Core Profession**		
Nurse	1 (12.5)	0 (00.0)
Vascular surgeon	7 (87.5)	0 (00.0)
GP trainee	0 (00.0)	1 (16.7)
General practitioner	0 (00.0)	5 (83.3)
**Grade**		
Band 8a	1 (12.5)	0 (00.0)
Senior House Officer	2 (25.0)	0 (00.0)
Specialty trainee	1 (12.5)	1 (16.7)
Speciality registrar	2 (25.0)	0 (00.0)
Consultant	2 (25.0)	0 (00.0)
GP	0 (00.0)	5 (83.3)
**Time since qualification**		
0-2 years	0 (00.0)	1 (16.7)
2 years + -5 years	0 (00.0)	1 (16.7)
5 years + -10 years	3 (37.5)	2 (33.3)
10 years + -15 years	5 (62.5)	2 (33.3)
**Time in current post**		
>6 months	5 (62.5)	1 (16.7)
7-12 months	0 (00.0)	0 (00.0)
13-24 months	2 (25.0)	2 (33.3)
25-36 months	0 (00.0)	1 (16.7)
37-48 months	1 (12.5)	0 (00.0)
49-60 months	0 (00.0)	0 (00.0)
61-75 months	0 (00.0)	1 (16.7)
13 years	0 (00.0)	1 (16.7)

Of the patient participants contacted by the researcher , (11/26) 42% agreed to take part in an interview (fifteen people declined) and (8/13) 82% of staff agreed to take part (1 person declined participation and 4 people did not respond to the invitation). Reasons for non-participation for patients included being unable to remember the intervention (n=5),

For peer review only

deteriorating health (n=7), just home from an in-patient admission (n=1), being admitted for angioplasty (n=1) and getting back on with life (n=1). The reason provided by providers for declining participation was moving to a new role and lack of time.

Secondary care clinicians and GPs spoke about all three elements of the intervention and patients provided insights on the education leaflet and GP action letter. The descriptive themes were mapped to implementation theory using the CFIR, to explore connections between the themes, and to compare findings across participant groups.

Using the CFIR to facilitate analysis

The CFIR comprises five domains that provide an overarching typology of implementation theory (in terms of what works where and why): i) intervention characteristics (features of the intervention such as complexity), ii) outer setting (the external context that might influence implementation such as health policy and the extent to which patient needs, as well as barriers and facilitators to meet those needs are known and prioritised), iii) inner setting (features of the implementing organisation such as leadership, implementation readiness), iv) characteristics of individuals involved in implementation (such as beliefs and knowledge about the intervention), iv) implementation process (Damschroder et al., 2009). The themes map to all five overarching CFIR domains across participant groups: intervention characteristics, outer setting, inner setting, characteristics of individuals and process. In summary, for intervention characteristics, all participant groups spoke about the advantage of implementing the care bundle, provided insights on improvements to the design quality and packaging, and acknowledged the need to adapt and tailor the intervention to meet the needs of individuals. Complexity was identified in terms of implementing the intervention across the primary-secondary care interface.

For outer setting, the specific needs of the patient group and barriers and facilitators to meet those needs were highlighted, with all participant groups explaining that a delayed referral to

For peer review only - http://bmjopen.bmj.com/site/about/guidelines.xhtml

secondary care is a barrier to implementing the intervention. Staff participants went on to speak about external policies and incentives that might influence the spread and uptake of the intervention. For inner setting, the structural characteristics and the quality of networks and communications within and across each organisation were identified by all groups as influences on implementation. For structural characteristics, participants explained that the ability to follow the advice in the education leaflet is often determined by the availability of supervised exercise programmes, difficulty ordering medication, and relying on carers for food shopping. For networks and communications, participants spoke about the need to work as a three-way team, and to have ongoing access to the specialist vascular team. For characteristics of individuals, staff values, attitude, knowledge, and skills to provide the intervention and patient readiness to take on the advice of the intervention were highlighted across participant groups. The patient-healthcare provider relationship was identified as a central influence on implementation and there was a particular emphasis on individual stage of change , with staff participants highlighting a need to engage patients in their care and to promote shared decision making. The mapping of themes across CFIR domains and participant groups is included in Online Data Supplement 2.

A final coding frame for patients, secondary care clinicians and GPs was developed following interpretive analysis. Because of space limitations, a summary of each theme is provided with subthemes being used to define each overarching theme; the full coding framework is included in Online Data Supplement 3. Four overarching themes were identified: *the potential to make a difference, a solution to address the gap in no man's land, prioritising and making it happen* and *personalised information and supportive conversations for taking on the advice.* Similarities and differences between participant groups are presented where they arise.

Theme 1: The potential to make a difference

For peer review only - http://bmjopen.bmj.com/site/about/guidelines.xhtml

GPs and secondary care clinicians felt that the LEGS intervention provides an opportunity to meet patient need and to support implementation of NICE guidance and spoke optimistically about using the intervention to improve patient outcomes and to standardise a minimum quality of care.

> *…it standardises the quality of care and standardisation is a big problem that we have in vascular surgery because guidance is so vague. So, I think it raises awareness and standardises that at least we ensure the patient gets a minimum of care* (QR15, secondary care clinician, UHL).

GPs and secondary care clinicians went on to explain how the intervention can be used to inform, empower, and support relationship building with patients.

> *I've gone through the leaflet with them… and knowing that the patient has understood I will spend a couple of minutes going through the leaflet to make sure. If they've got any questions I will try and take the opportunity to build my relationship with the patient so that they do understand* (QR08, secondary care clinician, UHL).

To further improve quality of care, providers identified the intervention as a way to support shared working across primary and secondary care, promoting a team approach and providing an opportunity for collaborative working. One GP explained how the '*patient is in the middle'* (QR23) and a secondary care clinician felt that '*we don't sing off the same hymn sheet'* (QR08). One GP explained that w*e don't ever see secondary care specialists* and a secondary care clinician explained *there's always this assumption that the GP will sort it out* (QR13). It was acknowledged that shared working provides an opportunity to understand each other's roles, to manage expectations and to learn from one another. Providers went on to report patient benefits in terms of access to specialist clinicians and working as a multi-disciplinary team, *I've always found that patients who have the opportunity to talk to multiple members of the medical team rather than a doctor or a nurse come out with better outcomes* (QR08, secondary care clinician, UHL).

Patients also spoke about the benefit of joined-up working, with one person stating;

> *…you attend lots of different clinics, you've got different people involved in your healthcare. It's not just one thing and everything's linked… it's what they call whole person care. It's not just your blood pressure, it's also the vascular team, it's the podiatrist, it's the diabetes specialist* (QR07, out-patient clinic, GSTT).

Theme 2: A solution to address the gap in no man's land

For peer review only - http://bmjopen.bmj.com/site/about/guidelines.xhtml

All participant groups spoke about a need to target patients earlier and identified a service provision gap in preventative medicine and early diagnosis. One secondary care clinician stated;

> *I think, almost targeting people earlier on, so claudicants would be really good… claudicants at the very first stage of their peripheral arterial disease, they're not really managed in secondary care, but they're also not really managed in primary care… this gap where they're in no man's land and they almost wait until it's severe enough to managed into secondary care* (QR18, secondary care clinician, UHL).

Patients explained how it took time to be referred to secondary care, and in one person's situation, it became an emergency;

> *…my doctor prescribed pills and some cream for my foot but they didn't do anything. And then it was only when I went back to podiatry after three months of constant pain, it was a case of just wait and see how things went… I went one day in the November, and they said you need to have your toe off almost immediately… they sent me to [hospital] and [hospital] say 'your toe needs to come off and it needs to come off in 20 minutes time'* (QR04, In-patient care, UHL).

Difficulties with patients presenting late, high secondary care referral thresholds resulting in delayed referrals to secondary care and a wait for secondary care to make the diagnosis, alongside lack of support for primary care to commence diagnostics in the community were also listed as barriers to accessing timely care. Some secondary care clinicians felt that the care of those with IC should be managed in primary care.

> *I think even the way that if the GP could start the patient, the first medical therapies, like, clopidogrel and statins, if he also could start the patient on exercise therapies. So, it should be implemented at the same kind of wave of the first medical therapy.* (QR19, secondary care clinician, UHL).

GPs offered their support and made a request to do more sooner. Some suggested using the intervention in general practice as part of a PAD prevention programme. They acknowledged the value of using the intervention to promote earlier conversations with patients, manage patient expectations, and support primary care to start best medical therapy. GPs also spoke about how being involved sooner, and completing the clinical checklist, could be used to support triage of referrals to secondary care. GPs noted that preventative medicine and health promotion is core to their practice, and that sharing information across services and with patients reinforces the message and supports implementation.

For peer review only

Theme 3: Prioritising and making it happen

Secondary care clinicians spoke about the need to prioritise the intervention alongside limited resources and the burden of work. One secondary care clinician explained, *it shouldn't be seen as an extra thing* (QR08). Individual levels of confidence, attitude, knowledge and experience of the LEGS bundle, or a similar checklist/patient education intervention influenced prioritisation with some staff identifying checklists as a quick and easy-to-use memory prompt. However, another reported that;

> *…people feel that it's just a tick-box exercise rather than a safety mechanism, and therefore no engagement with it… [it's] another piece of paper… I think part of the challenge is engagement with clinicians. Clinicians don't like threats to their autonomy… doctors, surgeons in particular are reticent, or certainly some are reticent to engage with checklists because they feel that it is doubling down or they already know this or that, you know, this simplifies things* (QR13, secondary care clinician, UHL).

Secondary care clinicians spoke about the extra resources required to deliver the intervention; it takes time to build relationships and to have conversations with patients. One secondary care clinician stated that finding the time to talk would require extra staff, this in an already stretched environment with staff shortages and long waiting times.

> *That will take more time, to make it personalised, to make it meaningful… you could almost imagine that they have a consultation and then some of those patients might benefit from sitting down with one of the specialist nurses… you could almost benefit from a 20-minute appointment after the standard clinic appointment to go through all of that. But again, that's a resource burden, that means that you've got to have someone extra in clinic* (QR13, secondary care clinician, UHL).

Secondary care clinicians felt that the intervention fits the in-patient environment neatly and could be used as a record of the out-patient clinic consultation. GPs felt that it could support the complexity of assessing patients remotely in the community. The need for training was recognised;

> *I think there is a huge lack in primary care education, and I think that goes to show by the starting people coming in are only on like a 40% of them are on Best Medical Therapy so I think a big thing to target would be primary care to inform them* (QR18, secondary care clinician, UHL).

For peer review only - http://bmjopen.bmj.com/site/about/guidelines.xhtml

GPs went on to request that all primary care staff be involved and not just GPs, and that the primary care training be delivered by the vascular team.

Theme 4: Personalised information and supportive conversations for taking on the advice

Providers and patients spoke about the delivery of the intervention, recognising the importance of offering personalised care and tailoring patient information to individual needs. Secondary care clinicians felt that the intervention should be adapted to identify realistic targets and individual goals, *or the advice rings hollow* (QR13), and to offer a different approach for each patient. Staff reported;

> *I think the hard thing as well is that with the LEGS bundle, sometimes you're giving it to someone who's just had an amputation or about to have an amputation so it's just to be mindful you're talking about things like exercise they look at you very disparagingly and that can be a bit tricky… I think it's a really good checklist and bundle for claudicants and I think that's where it really shines but I think for when you get to the stage of CLTI, I think those individuals often feel that they're too far gone for you to talk to them about lifestyle modification and things like that* (QR18, secondary care clinician, UHL).

Patients also spoke about the importance of timing in terms of when they received the lifestyle information.

> *If I was a bit younger and I'd got more get-up-and-go, I would gladly have a leaflet and go through it and take up advice and what have you. But as I am at the moment, my health is far from good* (QR14, in-patient care, UHL).

> *…it is a bit too late in that respect so I can only do what I can do. I mean yesterday I was out in the garden doing some weeding but I have to use a seat to do it* (QR05, in-patient care, UHL).

Patients explained that they like to receive information face-to-face and suggested that the education leaflet could be used as a supportive conversation guide. They emphasised the importance of the conversation that goes alongside the advice and information, and the relationship that they have with their health professional(s) in terms of asking questions and being able to talk about making changes like smoking cessation. One patient explained,

> *… the only thing I've got to do is drink and smoke and watch the tv – cause if I didn't have those three things, I would be pulling my hair out… to be quite honest, I'm quite happy at the moment to be carrying on the way I am. It might sound silly to you, but I*

For peer review only - http://bmjopen.bmj.com/site/about/guidelines.xhtml

enjoy smoking. I love a drink, I like my telly… if you're brow-beaten into packing up when you don't really want to, all this, it can have a great effect on people (QR14, in-patient care, UHL).

GPs and secondary care clinicians acknowledged the need to support patient preference, to involve patients in the conversation and to share decision making.

…you have to strike a balance, and therefore taking the patient's preferences into account is important. We assume that patients want to live the longest life they can, I'm not sure that's always correct. Some patients would rather accept that they live a slightly shorter life but with the benefit of enjoying certain things that we all know are less healthy (QR13, secondary care clinician, UHL).

It was acknowledged that everyone knows they need to stop smoking and it is easy to bombard people with information. Solutions such as motivational interviewing techniques were identified by one GP as a way to support engagement conversations. Secondary care clinicians felt that providing information on the benefits of following the advice versus taking pleasure away might help. For others, frank conversations that explain the risks help;

I think honesty is the most important aspect of this and bringing people in front of the data, highlighting the data, and actually not sugar coating anything. We know, for example, that if you have critical ischemia, so severe peripheral arterial disease, if your prognosis is worse, your five-year survival is worse than some kinds of cancer. So, if you tell someone that unless you actually take this intervention seriously, your likelihood of not being around in five years is very high. I know it sounds bleak, but it may be the wake-up call that someone needs. Or explaining that from the moment you have severe arterial peripheral disease, the likelihood of you losing your leg and being wheelchair bound may be an important thing to highlight as opposed to speaking in a circumspect way. I think just being honest and explaining the risks may be a way of engaging patients. (QR15, secondary care clinician, UHL).

A patient explained how the honest frank information helped;

The doctor actually threatened me. He said to me, if I'm going to do this operation, you got to stop smoking. So, I did. That helped, or I could lose my leg. I thought it was brilliant the way [the doctor] come out with it and I've never stopped thanking him for it. It works (QR06, out-patient care, GSTT).

Discussion

The aim of the study was to provide an understanding of the feasibility and implementation of the LEGS (LEaflet Gp letter Structured checklist) intervention, a complex intervention in the form of a healthcare bundle, designed to improve the care of patients with PAD in the NHS.

For peer review only - http://bmjopen.bmj.com/site/about/guidelines.xhtml

Our inductive approach to analysis found four overarching themes: i) *the potential to make a difference, ii) a solution to address the gap in no man's land, iii) prioritising and making it happen* and *iv) personalised information and supportive conversations for taking on the advice.* We used the Consolidated Framework of Implementation Research (CFIR) to guide our analysis, and all five CFIR domains are represented (13). The CFIR is well-placed for developing a model for implementing secondary care-initiated treatment in primary care (17).

All participants spoke positively about using the intervention to make a difference to patient care, as an opportunity to meet patient needs, and as a way to enhanc the quality of PAD care through improved working practices. This promotes a streamlined patient pathway from primary care to secondary care and to shared care. This reflects the value of an agreed pathway of care and shared partnership working that provides an opportunity for the intervention to be delivered as part of a wider team and to prioritise patient-centred care (17).

There was a consensus of optimism in participant accounts about the potential to make a difference. While barriers to seeking care for those with PAD are well-documented (18)(Tan et al., 2022. he findings confirm that the period between diagnosis of early-stage PAD (e.g., claudication) and subsequent potential diagnosis of advanced PAD with severe symptoms, is a time of no man's land, when patients do not receive any help, education, support, or appropriate medication to address their condition (9). As such, the benefits of the LEGS intervention were identified in terms of meeting the needs of this subgroup of patients, and to address the gap in care. GPs were optimistic that they can deliver the LEGS intervention with training and ongoing sustained communication with secondary care. The findings highlight the need to consider training requirements and systems for maintaining open primary-secondary care communication to support implementation. For example, education on tailoring the intervention for patients at both early and late stages of PAD, that can be used across primary and secondary care settings, similar to the model used with other chronic conditions (17, 19) Improving the awareness of PAD among healthcare providers and supporting patients to take

For peer review only - http://bmjopen.bmj.com/site/about/guidelines.xhtml

responsibility for their own health are therefore important determinants in the success of implementation (20).

While the three elements of the LEGS intervention have been designed to facilitate interaction between secondary and primary care following diagnosis of PAD, the intervention focus until now, has been on secondary care services, that is, hospital admission or out-patient clinic. However, our findings indicate that the intervention can be used to address the gap in service provision for patients who do not yet meet the referral criteria for secondary care services. We therefore propose to extend the use of the LEGS intervention across the primary-secondary care interface. Our findings also confirm the need for individualised patient care, supportive communication and shared decision making in order to take on the advice in a more effective way (21). The finalised LEGS intervention should therefore promote supportive conversation between patient and healthcare staff, both in primary and secondary care settings. The open approach to changing lifestyle and behaviour could be built on techniques like motivational interviewing, which is already commonly used in primary care practice (22). Lastly, our findings confirm that healthcare providers are optimistic and positive about the introduction of an intervention which will help them to deliver more consistent and guideline-based PAD treatment. The challenge now is to take this enthusiasm together with the lessons learned from this qualitative research to adapt the LEGS intervention before wider testing and adoption in the NHS. The main changes will relate to usability in primary care after diagnosis of early-stage PAD, facilitation of effective communication not only between primary and secondary care staff, but also patients and staff, as well as focussing on specific needs of patients by prioritising areas to focus on in an incremental or stepwise manner.

Strengths and limitations

The paper adds new knowledge on the development and implementation of a community and hospital intervention for PAD. It is important to note that the findings are specific to the

For peer review only - http://bmjopen.bmj.com/site/about/guidelines.xhtml

CHABLIS study. It is possible, however, to enhance transferability by describing the research context and assumptions, and by making connections between the analysis of participants accounts and claims in the extant literature.

While the sample size for this study may seem small, the volume of interview data we collected is unique for this patient population. Many factors influence the success of recruiting participants in research and engaging vulnerable patient populations brings additional complexity (23). PAD is a chronic and progressive disease with burdensome symptoms including physical disability, cardiovascular risks and mortality (24). Those who live with PAD are vulnerable because they are living with extreme frailty, often with the threat or result of limb loss, and with risks associated with poorer socio-economic conditions (25). Alongside, patients with PAD might be less receptive to engaging with research because they experience specific barriers to accessing healthcare meaning that taking part in research alongside managing health care and health needs might be considered onerous (26).

A strength is the focus on rigor and triangulation of participant perspectives. We also acknowledged the complexity of intervention implementation across the secondary-primary care interface and therefore used a systems research perspective for addressing implementation at the outset of the research. We used implementation theory to guide analysis and to provide a layered coding approach, to ensure that intervention and implementation recommendations are novel, grounded in the data, and useful (13).

While the anonymity of telephone use can allow participants to disclose sensitive information, telephone interviews have received criticism for compromising interviewer/participant rapport and interaction, and for limiting contextual data due to the absence of face-to-face contact and visual cues. However, this method of data collection is convenient, in that it is flexible (in terms of time and location), accessible (i.e., remote research conducted during the COVID-19 pandemic), and allows for a wide reach (e.g., accessing a diverse population from both urban and rural areas) (27).

For peer review only - http://bmjopen.bmj.com/site/about/guidelines.xhtml

Conclusions

The LEGS intervention can be tailored for use at early and late stages of PAD, can be provided across primary and secondary care settings, and provides an opportunity to promote shared working across the primary-secondary care interface. These findings are important to the specifics of the CHABLIS study but can also go some way in informing the wider learning for other interventions that are implemented across the primary-secondary care interface.

Funding

This article presents independent research funded by National Institute for Health Research for Patient Benefit (RfPB) Programme (funder’s reference: NIHR202008). The views expressed are those of the author(s) and not necessarily those of the NIHR or the Department of Health and Social Care.

Competing interests

The authors declare that they have no competing interests.

Authors' contributions

CL contributed to the qualitative study design, coordinated the study, led data collection and analysis, and drafted the manuscript. AS is principal investigator to the CHABLIS study, acquired funding, participated in the study design, contributed to data collection, and drafted the manuscript. PS contributed to data collection and reviewed the manuscript. RB and BB drafted the manuscript. EW contributed to data collection and reviewed the manuscript. VL designed the qualitative study, provided oversight on study coordination, data collection and analysis, and drafted the manuscript. All authors read and approved the final manuscript.

Consent for publication

Consent for publication has been obtained from all research participants.

For peer review only - http://bmjopen.bmj.com/site/about/guidelines.xhtml

Word count

4,775

Availability of data and materials

This study uses data (containing potentially identifying and/or sensitive information) collected from a small group of staff participants and a vulnerable patient population, and involves indirect identifiers (such as sex, ethnicity, location, etc.) that may risk the identification of study participants. Sharing data outside of the anonymised excerpts and quotations included in the paper will violate the agreement to which the participants consented.

Acknowledgements

We would like to thank all patients, secondary care staff and GPs who participated in this study and who generously gave their time and honest thoughts. We would like to thank the local research teams for their support in recruiting to this qualitative study and we are also grateful to the NIHR Clinical Research Network Research Delivery Managers that helped to recruit GP participants.

Ethics Statement

The research was reviewed and approved by the NHS Wales Research Ethics Committee (REC), reference number20/WA/0319 and the NHS Health Research Authority (HRA).

References

1. Herrington W, Lacey B, Sherliker P, Armitage J, Lewington S. Epidemiology of Atherosclerosis and the Potential to Reduce the Global Burden of Atherothrombotic Disease. Circ Res. 2016;118(4):535-46.

2. Cea-Soriano L, Fowkes FGR, Johansson S, Allum AM, García Rodriguez LA. Time trends in peripheral artery disease incidence, prevalence and secondary preventive therapy: a cohort study in The Health Improvement Network in the UK. BMJ Open. 2018;8(1):e018184.

3. Dhaliwal G, Mukherjee D. Peripheral arterial disease: Epidemiology, natural history, diagnosis and treatment. Int J Angiol. 2007;16(2):36-44.

For peer review only

4. Parvar SL, Fitridge R, Dawson J, Nicholls SJ. Medical and lifestyle management of peripheral arterial disease. J Vasc Surg. 2018;68(5):1595-606.

5. Nehler MR, Duval S, Diao L, Annex BH, Hiatt WR, Rogers K, et al. Epidemiology of peripheral arterial disease and critical limb ischemia in an insured national population. J Vasc Surg. 2014;60(3):686-95.e2.

6. Dua A, Lee CJ. Epidemiology of Peripheral Arterial Disease and Critical Limb Ischemia. Tech Vasc Interv Radiol. 2016;19(2):91-5.

7. Morley RL, Sharma A, Horsch AD, Hinchliffe RJ. Peripheral artery disease. Bmj. 2018;360:j5842.

8. National Institute for Health and Care Excellence. Peripheral arterial disease: diagnosis and management. 2020.

9. Saratzis A, Jaspers NEM, Gwilym B, Thomas O, Tsui A, Lefroy R, et al. Observational study of the medical management of patients with peripheral artery disease. Br J Surg. 2019;106(9):1168-77.

10. Skivington K, Matthews L, Simpson SA, Craig P, Baird J, Blazeby JM, et al. A new framework for developing and evaluating complex interventions: update of Medical Research Council guidance. Bmj. 2021;374:n2061.

11. Tong A, Sainsbury P, Craig J. Consolidated criteria for reporting qualitative research (COREQ): a 32-item checklist for interviews and focus groups. Int J Qual Health Care. 2007;19(6):349-57.

12. Braun V, Clarke V. Using thematic analysis in psychology. Qualitative Research in Psychology. 2006;3(2):77-101.

13. Damschroder LJ, Aron DC, Keith RE, Kirsh SR, Alexander JA, Lowery JC. Fostering implementation of health services research findings into practice: a consolidated framework for advancing implementation science. Implement Sci. 2009;4:50.

14. Braun V, Clarke V. One size fits all? What counts as quality practice in (reflexive) thematic analysis? Qualitative Research in Psychology. 2021;18(3):328-52.

15. QSR International Pty Ltd. NVivo. 2018.

16. Bammer G. Key issues in co-creation with stakeholders when research problems are complex. Evidence & Policy. 2019;15(3):423-35.

17. Le Boutillier C, Ahmed H, Patel V, McPhail M, Carter B, Ward C, et al. General Practitioner perspectives on factors that influence implementation of secondary care-initiated treatment in primary care: exploring implementation beyond the context of a clinical trial. PLoS One. 2022.

18. Meffen A, Houghton JSM, Nickinson ATO, Pepper CJ, Sayers RD, Gray LJ. Understanding variations in reported epidemiology of major lower extremity amputation in the UK: a systematic review. BMJ Open. 2021;11(10):e053599.

19. Smith SM, Cousins G, Clyne B, Allwright S, O'Dowd T. Shared care across the interface between primary and specialty care in management of long term conditions. Cochrane Database Syst Rev. 2017;2(2):Cd004910.

20. Bridgwood BM, Nickinson AT, Houghton JS, Pepper CJ, Sayers RD. Knowledge of peripheral artery disease: What do the public, healthcare practitioners, and trainees know? Vasc Med. 2020;25(3):263-73.

21. Barry MJ, Edgman-Levitan S. Shared decision making--pinnacle of patient-centered care. N Engl J Med. 2012;366(9):780-1.

22. Rubak S, Sandbaek A, Lauritzen T, Christensen B. Motivational interviewing: a systematic review and meta-analysis. Br J Gen Pract. 2005;55(513):305-12.

For peer review only - http://bmjopen.bmj.com/site/about/guidelines.xhtml

23. Le Boutillier C, Snowdon C, Patel V, McPhail M, Ward C, Carter B, et al. Using a theory-informed approach to explore patient and staff perspectives on factors that influence clinical trial recruitment for patients with cirrhosis and small oesophageal varices. PLoS One. 2022;17(2 February).

24. Adam DJ, Beard JD, Cleveland T, Bell J, Bradbury AW, Forbes JF, et al. Bypass versus angioplasty in severe ischaemia of the leg (BASIL): multicentre, randomised controlled trial. Lancet. 2005;366(9501):1925-34.

25. Brostow DP, Hirsch AT, Kurzer MS. Recruiting older patients with peripheral arterial disease: evaluating challenges and strategies. Patient Prefer Adherence. 2015;9:1121-8.

26. Tan TW, Crocker RM, Palmer KNB, Gomez C, Armstrong DG, Marrero DG. A qualitative study of barriers to care-seeking for diabetic foot ulceration across multiple levels of the healthcare system. J Foot Ankle Res. 2022;15(1):56.

27. Irvine A. Duration, Dominance and Depth in Telephone and Face-to-Face Interviews: A Comparative Exploration. International Journal of Qualitative Methods. 2011;September:202-20.

For peer review only

Interview Topic Guide for Patients

Title of the study: A **C**ommunity and **H**ospital c**A**re **B**undle to improve the medical treatment of c**L**aud**I**cation and critical limb i**S**chaemia. **(**The CHABLIS study)

Welcome and introduction

[Seek consent to continue and to audio-record the interview (if applicable). Participant has to provide written informed consent (see separate form). Let them know that no personal identifiable data will be recorded and a participant number will be allocated to them.]

The main aim of this study is to assess whether a dedicated intervention (a care bundle) can be used in the routine care of patients who present with symptomatic Peripheral Arterial Disease (PAD). The care bundle includes a number of checklists and leaflets for doctors and patients. The intervention is aimed at reducing the risk of major cardiovascular events (i.e. heart attacks and strokes), and preventing amputation, by making sure patients with PAD receive the appropriate treatment and management as recommended by national guidelines. We have called this intervention LEGS (the LEGS bundle), given that the aim is to save legs from amputation and improve vascular care.

The LEGS intervention:

ELEMENT 1: In-patient checklist/Outpatient Checklist

The doctors who see you will be given a checklist of the common things that patients with PAD should be offered in terms of medications and / or tests. All of these suggestions are already part of what is the standard NHS care for PAD. Using your medical notes, we will record your blood pressure, your medications (current), you medical history, previous surgeries, imaging results, your routine blood test results (cholesterol, lipid profile, full blood count), smoking status, and demographic information (age, height, weight, gender). This is standard information that the NHS already collects in order to be able to offer you treatment for your blocked arteries.

ELEMENT 2: 2nd (follow-up) letter to the GP

ELEMENT 3: Patient & carers' leaflet

You will be given a leaflet which will provide you with information about PAD and other things you can do to help reduce your risks.

You will have an appointment to see us again in six months to check your legs and your overall health. This is standard for someone with PAD. We will again record your blood pressure, your medications (current), your medical history, previous surgeries, imaging results, your routine blood test results (cholesterol, lipid profile, full blood count), smoking status, and demographic information (age, height, weight, gender). During that appointment we will ask you to fill in a questionnaire which assesses how well people are taking their prescribed medication, called the MARS questionnaire.

For peer review only - http://bmjopen.bmj.com/site/about/guidelines.xhtml

The interview will explore your experience of receiving the intervention. We will ask about your opinion of NHS care relating to your leg artery problems in greater detail. We will ask you how you have found the management of your care at hospital and your GP practice, what your thoughts are of the information leaflet, and if you think it has been of any benefit to you or not.

Questions:

1. How have you found the management of your care at hospital and your GP practice?

- the initial consultation between patient and healthcare professional after the diagnosis of peripheral arterial disease has been made
    - what should be covered in the initial consultation?
    - Anything missing from this consultation?
    - Are you able to ask questions?

- the frequency and duration of follow-up

2. What are your thoughts on the information leaflet?

- Is there enough information about peripheral arterial disease? Too much information? Is the information clear? Signposts for where to go for more information?
- Is there anything on the leaflet that you don't understand or would like more information about?
- What would you like to see included in a leaflet designed to inform patients about peripheral arterial disease care and therapies?
- What about the length of the leaflet?

3. Do you think it has been of any benefit to you or not?

4. In your opinion, are there any factors that might influence someone's ability to follow the guidance in the patient information leaflet? (i.e. barriers to taking medication, managing diet)

- Is there anything that could help? Solutions to any barriers for following the advice?

5. Any ideas for improving any of the elements of the intervention?

   ELEMENT 1: Inpatient/outpatient checklist (& patient consultation)
   ELEMENT 2: 2nd (follow-up) letter to the GP (& patient consultation)
   ELEMENT 3: Patient & carers' leaflet

6. What do you need for your health to improve? (wider experiences and perceived needs)

Anything not covered? Is there anything that we haven't covered in the interview that you think we should know or think about?

Closing and thanks - check that the participant is still happy for you to use all the information provided and offer the possibility to erase sections of the recording.

For peer review only - http://bmjopen.bmj.com/site/about/guidelines.xhtml

UNIVERSITY OF LEICESTER

KING'S College LONDON

Thank them for their time and contribution.

Interview Topic Guide for Secondary Care Clinicians & GPs

Title of the study: A **C**ommunity and **H**ospital c**A**re **B**undle to improve the medical treatment of c**L**aud**I**cation and critical limb i**S**chaemia. **(**The CHABLIS study)

Welcome and introduction

[Seek consent to continue and to audio-record the interview (if applicable). Participant has to provide written informed consent (see separate form). Let them know that no personal identifiable data will be recorded and a participant number will be allocated to them.]

The main aim of this study is to assess whether a dedicated intervention (a care bundle) can be used in the routine care of patients who present with symptomatic Peripheral Arterial Disease (PAD). The care bundle includes a number of checklists and leaflets for doctors and patients. The intervention is aimed at reducing the risk of major cardiovascular events (i.e. heart attacks and strokes), and preventing amputation, by making sure patients with PAD receive the appropriate treatment and management as recommended by national guidelines. We have called this intervention LEGS (the LEGS bundle), given that the aim is to save legs from amputation and improve vascular care.

ELEMENT 1: In-patient checklist/Outpatient Checklist

ELEMENT 2: 2nd (follow-up) letter to the GP

ELEMENT 3: Patient & carers' leaflet

The interview will explore your experiences/perspectives of delivering the intervention. This will help us to evaluate the ability/feasibility of staff in delivering the intervention, overall impression, validity, usability, acceptability and engagement with the intervention. It will include barriers to engagement or delivery and ideas for improving the intervention.

This is a GUIDE for topics. Further topics will and can be explored if necessary.

The purpose of the study is to find out about staff views and experiences of implementing the LEGS intervention as a part of routine clinical care. This provides us with information on factors that facilitate or hinder implementation and on possible solutions to overcome any identified barriers.

Key objectives of the interview session: i) to identify barriers to and facilitators of each of the key intervention targets; ii) identify issues around delivery, training needs and competencies.

We would like to ask you about what helps and what hinders success in implementing the LEGS intervention as a part of routine clinical care.

- To get started, we would like to ask about your views on existing NICE guidance and factors that might influence implementation of NICE guidance on cardiovascular risk reduction amongst patients with peripheral arterial disease. Please comment on: i)

For peer review only - http://bmjopen.bmj.com/site/about/guidelines.xhtml

prescribing, ii) awareness of NICE guidance by primary and secondary care, iii) adherence by patients (when they have been prescribed their medication), iv) follow-up practices in primary and secondary care.

- What do you think about using the LEGS intervention with this group of patients? Are there any benefits? Or challenges?

Understanding implementation

Aims:
1) To explore what participants think helps or hinders implementation.

2) To explore what participants' identify as potential solutions to implementation barriers.

What helps implementation?

- What factors do you perceive might support implementing the LEGS intervention in routine clinical practice?
- What helps/would help you (and your colleague) to provide the intervention a part of routine clinical care?
- What are your views on the LEGS intervention and the acceptability and feasibility of delivering the intervention?
- Do you feel that you have the knowledge and skills to deliver this intervention?
- What implementation strategies might help? Training?

What prevents or hinders implementation?

- What prevents or hinders success in implementing the LEGS intervention as a part of routine clinical care?
- Are there/or might there be challenges around implementation?
- What would further support you to implement the intervention?

Prompts / Continuation Questions:

- What resources would better equip you to implement the intervention?
- What would you say is the ideal environment to implement the LEGS intervention in?

Implementation solutions

- What solutions would you recommend to address these identified barriers?
- Any other influences on implementation? COVID? Other clinical priorities?
- Any changes needed to the intervention? Ideas for improving the intervention?

  (Can you advise us on the 3 most important things you would like to see covered in a patient leaflet for patients with peripheral arterial disease? Can you please advise us

For peer review only - http://bmjopen.bmj.com/site/about/guidelines.xhtml

on the 3 most important things you would like to see covered in a checklist which supports the delivery of peripheral arterial disease care for patients?)

This interview was designed to help us understand the extent to which the CHABLIS intervention has been implemented, and what has helped or hindered you in doing this. Are there any other important points that you would like to discuss before we close the discussion?

Closing and thanks - check that the participant is still happy for you to use all the information provided and offer the possibility to erase sections of the recording.

Thank them for their time and contribution.

Ensure that they have received a PIS and the CF has been handed back for photocopying.

For peer review only

For peer review only - http://bmjopen.bmj.com/site/about/guidelines.xhtml

CFIR Domains and the LEGS bundle	Patients	Secondary care clinicians	GPs
**THE LEGS BUNDLE**			
Element 1: Clinical checklist		X	X
Element 2: Patient and carer education leaflet	X	X	X
Element 3: GP action letter	X	X	X
**CFIR DOMAINS**			
**Domain 1: INTERVENTION CHARACTERISTICS**	**X**	**X**	**X**
**1.1 Intervention source:** Perception about whether the intervention is externally or internally developed.			
The whole bundle		X	X
I sat down with the developers, and they gave me the background		X	
Perception about how the intervention was developed			X
Involving stakeholders in the development process will promote uptake			X
**1.2 Evidence strength and quality:** Perceptions of the quality and validity of evidence supporting the belief that the intervention will have desired outcomes.			
Clinical checklist		X	
Built on existing NICE guidance		X	
The guidance is vague		X	
The guidance is clear, but we don't follow it		X	
**1.3 Relative advantage (or not):** Perception of the advantage of implementing the intervention versus an alternative solution.			
The whole bundle		X	X
Improves care and outcomes		X	
Standardises a minimum quality of care		X	
Allows us to make a difference to patients		X	
Joins up primary and secondary care working			X
Improves awareness of NICE guidance in primary & secondary care		X	X
Improves awareness and understanding of PAD		X	X
Clinical checklist		X	
Builds your relationship with the patient		X	
Improves medication compliance		X	
Patient and carer education leaflet	X	X	X
Patients are empowered by being informed		X	
I don't want any more leaflets	X		
District nurses can answer my questions	X		
A conversation guide for primary care			X
Existing relationships to reinforce the message			X
GP action letter		X	X
A standardised letter with clear information		X	
Useful for non-medical led community vascular clinics			X
Repeat GP action letter is a duplicate, adds to the workload and sets the wrong tone			X
It gives the impression that secondary care do not trust GPs			X

CFIR Domains and the LEGS bundle	Patients	Secondary care clinicians	GPs
**1.4 Adaptability:** The degree to which an intervention can be adapted, tailored, refined, or reinvented to meet local needs.			
Clinical checklist		X	
Adapting to the environment		X	
Fits the in-patient environment neatly		X	
Incorporate into a universal admission checklist		X	
Limited access to blood pressure monitoring or cholesterol tests in out-patient clinic		X	
Promote checklist in primary care to be used alongside secondary care referral letter		X	
Patient and carer education leaflet	X	X	X
Offer personalised care and tailor patient education to individual needs		X	X
A different approach for each type of patient			X
Different information for each patient			X
Existing info most likely to suit those with intermittent claudication			X
Offer realistic targets or the advice rings hollow		X	
Consider co-exiting morbidities			X
Too much information	X		
Change information layout to provide summary at outset		X	
Missing information	X		X
Understanding what causes PAD and preventing further blockages	X		X
Managing the psychological impact of PAD	X		
Maintaining movement and physiotherapy input	X		
Impact on work, social and family life	X		
Acknowledgement of reliance and impact on family	X		
Carer and family support	X		
Managing future risks and when to seek support		X	X
**1.5 Trialability:** The ability to test the intervention on a small scale in the organisation, and to be able to reverse course (undo implementation) if warranted.			
Clinical checklist	X	X	
A quick quality check		X	
Patient and carer education leaflet	X	X	
Leaflet is a resource for referring back	X	X	
**1.6 Complexity:** Perceived difficulty of implementation, reflected by duration, scope, radicalness, disruptiveness, centrality, and intricacy and number of steps required to implement.			
Clinical checklist		X	X
Simplicity		X	
Improvements that make it even simpler		X	
It needs to be a bit more, no and why		X	X
GP action letter	X	X	X
A template for new staff		X	
A time-consuming manual coding process with potential for error			X
Waiting times for primary care/test appointments	X		X
Difficult to assess patients remotely			X

CFIR Domains and the LEGS bundle	Patients	Secondary care clinicians	GPs
Lack of continuity			X
60 calls and 40 letters a day			X
Up to three months to receive hospital letters			X
Patients to hand deliver letter to GP surgery			X
**1.7 Design quality and packaging:** Perceived excellence in how the intervention is bundled, presented, and assembled.			
Clinical checklist		X	
Working across two different systems - Paper and electronic		X	
Paper checklists tend to get missed		X	
Electronic option required		X	
Patient and carer education leaflet	X	X	X
Accessibility	X		
Stored in a safe place	X		
Lost the leaflet	X		
Would be improved if available by email	X		
Presentation & format	X		
Paper leaflet		X	
Larger print has been requested		X	
Language can be hard to understand	X		X
Write for a lower reading age and provide additional information or additional resources for people who are able to access more detailed information			X
Keep it simple, give less information, in different formats and in a way that is understandable			X
Avoid didactic language and reframe the approach			X
There's a lot of 'do nots'			X
Ensure accuracy of information			X
Confusing dietary advice			X
Provide more infographics and pictures	X	X	X
Include diversity in visuals (e.g., pictures of leg ulcers/skin changes in different skin colours)		X	
Add web links			X
Could be supported by existing NHS software like QRX, direct information to patient phones			X
Offer an audio and video version	X	X	X
Patient information to be offered as a group intervention	X	X	
Opportunity for group to be peer-led		X	
GP action letter			X
Same information and same system across primary and secondary care			X
Include information on the discussion around contraindications and side effects of any newly prescribed medication with patient			X
Any contraindications primary care should be aware of, useful for clarification			X
Offered and accepted/declined smoking cessation referral			X
Provide a clear plan for if the situation deteriorates			X
Copy of letter to patient – shared message – reinforcement			X

For peer review only

CFIR Domains and the LEGS bundle	Patients	Secondary care clinicians	GPs
**1.8 Cost:** Costs of the intervention and costs associated with implementing that intervention including investment, supply, and opportunity costs.			X
It is not commissioned or financially viable			X
Primary care time			X
Maintaining equipment			X
**Domain 2: OUTER SETTING**	X	X	X
**2.1 Patient needs and resources:** The extent to which patient needs, as well as barriers and facilitators to meet those needs are known and prioritised.			
The whole bundle	X	X	X
Working as part of a whole team			X
Promoting joined up working across primary and secondary care		X	X
Enabling specialist care from different people			X
Expectations and understanding of each other's roles			X
Timely access back into secondary care if needed			X
Secondary care oversight			X
Accessible referral pathway	X		X
We can't make a referral anyway			X
Secondary care referral thresholds too high			X
You don't fit the criteria so wait until it gets worse			X
Delayed referral to secondary care	X	X	X
Presenting too late		X	
Can I do some of the diagnostics in the community?			X
Time wastage		X	X
A geographical consideration	X		X
Locality of secondary care services	X		X
Transport needs	X		X
Clinical checklist		X	X
Use in primary care to start best medical therapy		X	
Would promote earlier conversations with patients in primary care		X	
Use at point of admission and discharge for in-patients		X	
Adapt for primary care to support referral			X
Patient and carer education leaflet	X	X	X
Patient education provided too late	X	X	
Too far gone to talk about lifestyle modification		X	
Level of functioning & mobility - I can only do what I can do	X		
It is crucial whenever it happens		X	
Introduced sooner in primary care as part of a PAD prevention programme			X
Informing patients to reduce the chance of presenting late/in crisis			X
Managing patient expectations			X
**2.2 Cosmopolitanism:** The degree to which an organisation is networked with other external organisations.			
The whole bundle		X	
Shared primary & secondary care working		X	
Patient and carer education leaflet			X

For peer review only

CFIR Domains and the LEGS bundle	Patients	Secondary care clinicians	GPs
Copy of leaflet to primary care to reiterate message			X
GP action letter		X	
Clear open communication across community and hospital services		X	
Joined up clinical record systems		X	
We don't sing off the same hymn sheet		X	
**2.3 External policies and incentives:** A broad construct that includes external strategies to spread interventions including policy and regulations (governmental or other central entity), external mandates, recommendations and guidelines, pay-for-performance, collaboratives, and public or benchmark reporting.			
The whole bundle		X	
Additional funding		X	
**Domain 3: INNER SETTING**	X	X	X
**3.1 Structural characteristics:** The social architecture, age, maturity, and size of an organisation.			
The whole bundle		X	X
Impact of staff shortages		X	
Reduced staffing due to covid		X	
Involving the multi-disciplinary team			X
GP action letter		X	X
Time delay between writing and getting to primary care		X	X
Patient and carer education leaflet	X	X	X
Being able to follow the advice	X	X	
Difficulty ordering repeat medication	X		
Relying on others for food shopping	X		
Availability of supervised exercise		X	
It's easy to bombard somebody with information			X
**3.2 Networks and communications:** The nature and quality of webs of social networks and the nature and quality of formal and informal communications within an organisation.			
The whole bundle	X	X	X
A team approach		X	
Building relationships			X
Clear communication			X
The patient is in the middle			X
Involve all multi-disciplinary professionals (including pharmacists, nurses, physios)		X	
Access to the vascular team or not	X		X
Advice and information	X		X
**3.3 Culture:** Norms, values, and basic assumptions of a given organisation.			
The whole bundle		X	
Target patients earlier		X	
The gap in no man's land		X	
**3.4 Implementation climate - tension for change:** The degree to which stakeholders perceive the current situation as intolerable or needing change.			
The whole bundle		X	X
Primary care needs to do more sooner		X	
Secondary care referral threshold is too high			X
**3.5 Implementation climate - Compatibility:** The degree of tangible fit between meaning and values attached to the intervention by involved individuals, how those align with individuals' own norms, values, and			

For peer review only

CFIR Domains and the LEGS bundle	Patients	Secondary care clinicians	GPs
perceived risks and needs, and how the intervention fits with existing workflows and systems.			
The whole bundle			X
Existing systems in place for primary care medication management and monitoring			X
Provide 28 days medication to allow for delay in seeing GP			X
Preventative medicine and health promotion is core to primary care practice			X
Ability to offer smoking cessation support			X
Local variation in set-up of smoking cessation, dietary advice, exercise therapy			X
No formal set-up in primary care to pick it up			X
GP relationship with patients			
Not commissioned			
Secondary care to initiate smoking cessation referral			X
National protocol for weight reduction			X
Existing relationships with Diabetes nurses/clinics			X
**3.6 Implementation climate - Relative priority:** Individuals' shared perception of the importance of the implementation within the organisation.			
Clinical checklist		X	
Take the opportunity		X	
It shouldn't be seen as an extra thing		X	
A threat to autonomy		X	
Checklists are tick-box exercises rather than safety mechanisms		X	
**3.7 Implementation climate - Learning climate**			
The whole bundle			X
Two-way learning			X
Secondary care can learn from this too			X
Holistic picture of medication profile, side effects, competing medication			X
Teams are not static so require ongoing access to training		X	X
**3.8 Readiness for implementation – leadership engagement:** Commitment, involvement, and accountability of leaders and managers with the implementation.			
The whole package		X	
Provide a named leader		X	
External oversight is preferable		X	
**3.9 Readiness for implementation – available resources:** The level of resources dedicated for implementation and on-going operations including money, training, education, physical space, and time.			
Clinical checklist		X	X
Checklists are quick		X	
Managing it alongside the burden of work		X	
The assumption that the GP will sort it – putting it back into GP land		X	X
Lack of access to testing equipment in community			X
Limited community workforce (i.e., reduced number of district nurses) capacity for conducting ABPIs			X
Secondary care are gatekeepers for ABPIs and duplexes			X

For peer review only

CFIR Domains and the LEGS bundle	Patients	Secondary care clinicians	GPs
Waiting for secondary care to make diagnosis			X
Patient and carer education leaflet		X	
Time to talk requires extra staff		X	
GP action letter		X	X
Time burden and impact of dictating long clinic letters		X	
Joining it up - We've done our bit and now pass it on to the GP			X
Routine practice or adding to the workload			X
Feels top down and with didactic language			X
Keep the GP onside and change phraseology			X
**3.10 Readiness for implementation - access to information and knowledge:** Ease of access to digestible information and knowledge about the intervention and how to incorporate it into work tasks.			
The whole bundle		X	
Training on the purpose of the intervention		X	
Written information for those unable to attend training		X	
Prioritise training and supervision for junior staff – the people who do the day-to-day work		X	
Consider involving medical students		X	
Highlight the link between PAD and cardiovascular death		X	
Education on managing CLTI and Non-CLTI patients in primary care		X	
Knowing the risk factors for CLTI and how to make a referral to secondary care		X	
Taking an ABPI in primary care		X	X
Support the triage for secondary care			X
We're not commissioned to do it			X
Visibility of vascular teams at primary care conferences		X	
Learning on the job		X	X
Informal training to colleagues i.e., when seeing a medical outlier		X	
Use available supervision/debriefing opportunities			X
Webinar-ed out		X	
Training delivered by vascular team to primary care			X
Collaborative working and building relationships			X
We don't ever see secondary care specialists			X
Involve all primary care staff and not just GPs			X
Learning gap in exercise and lifestyle modification			X
**DOMAIN 4: CHARACTERISTICS OF INDIVIDUALS**			
The whole bundle	X	X	X
Taking on the advice	X	X	X
Familiarity and understanding of PAD		X	X
**4.1 Knowledge and beliefs about the intervention**			
Clinical checklist		X	X
Prioritising the checklist (or not)		X	
Previous experience of using checklists and identifying benefits		X	
Checklists are a useful memory prompt		X	
Patient and carer education leaflet	X		

For peer review only

CFIR Domains and the LEGS bundle	Patients	Secondary care clinicians	GPs
Targets that are impossible to reach	X		
**4.2 Self-efficacy**			
The whole bundle		X	
Confidence to implement guidelines		X	
**4.3 Individual stage of change**			
Clinical checklist		X	
Put myself forward to find out more		X	
Patient and carer education leaflet	X	X	X
If I was younger and I had more get-up-and-go	X		
It's down to willpower at the end of the day (I like smoking, I like drinking, I like the telly) – patient engagement & stage of change – resistance from patients	X	X	X
The only thing left is something nice to eat	X		
Resistance from patients		X	
Secondary care doctors tell people to do things			X
Everybody knows they need to stop smoking			X
**4.4 Individual identification with organisation:** how individuals perceive the organization and their relationship and degree of commitment with that organisation.			
The whole bundle		X	
Joined up working with primary care		X	
Patient and carer education leaflet	X	X	X
The patient – health professional relationship	X	X	
It takes time to build the relationship and have the conversation		X	
Clear open communication	X		
Frank conversations for some	X	X	
Be honest and explain the risks		X	
Information on the benefits versus taking pleasure away		X	
Striking a balance to optimise people's quality of life. You can't take everything away.		X	
Patient preferences and shared decision making		X	
Online tools to support the clinical conversation		X	
Language and approach used to engage people			X
Consider use of motivational interviewing techniques in secondary care			X
GP action letter			X
Partnership working			X
Avoid the extra step and order the test in secondary care			X
**4.5 Other personal attributes:** other personal traits such as tolerance of ambiguity, intellectual ability, motivation, values, competence, capacity, and learning style.			
Clinical checklist		X	
Team members who prioritise PAD		X	
Patient and carer education leaflet	X		
Living with other health conditions alongside (arthritis, diabetes)	X		
Managing the pain	X		
Medication side-effects	X		

For peer review only

CFIR Domains and the LEGS bundle	Patients	Secondary care clinicians	GPs
**DOMAIN 5: PROCESS**			
**5.1 Engaging:** Attracting and involving appropriate individuals in the implementation and use of the intervention through a combined strategy of social marketing, education, role modelling, training, and other similar activities.			
The whole bundle		X	
Championing the cause		X	
Doing the bare minimum and going home on time		X	
Using research to demonstrate a difference		X	
Involving stakeholders at an early stage		X	
Clinical checklist		X	
Posters in clinic to remind staff		X	
**5.2 Executing:** Carrying out or accomplishing the implementation according to plan.			
Patient and carer education leaflet	X		
A direct contact number for the vascular team	X		
Primary care capacity	X		
My GP and practice nurses keep an eye on me	X		
Sometimes it can take weeks to get them to answer a query over the phone	X		
GP action letter	X		
It can take weeks to get information on their system	X		
It's lots of different clinics and different people involved in your healthcare (whole person care)	X		

CFIR matrix and distribution of descriptive themes

For peer review only

Full coding framework

Theme 1: The potential to make a difference

1.1 An opportunity to meet patient need

1.1.1 Improves care and outcomes
1.1.1.1 Standardises a minimum quality of care
1.1.2 Improves awareness and understanding of PAD
1.1.3 Improves awareness of existing NICE guidance
1.1.3.1 Provides a tool to support implementation of NICE guidance
1.1.3.1.1 The guidance is clear but we don't always follow it
1.1.4 Builds your relationship with the patient
1.1.4.1 Empowers patients by being informed

1.2 Shared primary and secondary care working

1.2.1 Working as a part of a whole team
1.2.1.1 Allows for secondary care oversight
1.2.1.2 An opportunity for collaborative working
1.2.1.2.1 Managing expectations and understanding each other's roles
1.2.1.2.2 We don't sing off the same hymn sheet
1.2.1.2.2.1 The patient is in the middle
1.2.1.3 Multidisciplinary working
1.2.1.3.1 Enabling specialist care from different people
1.2.1.4 Promoting continuity of care
1.2.1.4.1 There's lots of different clinics and different people involved in your care
1.2.1.4.2 Timely access back into secondary care if needed
1.2.2 Joining up and building relationships across the team
1.2.2.1 We don't ever see secondary care specialists
1.2.2.2 Access to the vascular team (or not)
1.2.2.2.1 A direct contact number for the vascular team
1.2.2.3 We've done our bit and now it's over to the GP
1.2.3 Clear open communication across community and hospital services
1.2.4 Working together and learning together
1.2.4.1 An opportunity for two-way learning
1.2.4.1.1 Secondary care can learn from this too
1.2.4.1.1.1 Support for screening questions on prescribing and contraindications
1.2.4.1.1.1.1 Holistic picture of medication profile, side effects, competing medication
1.2.4.1.1.2 Familiarity (or not) with reviewing cholesterol and HbA1c's – it's a GP thing

Theme 2: A solution for addressing the gap in no man's land

2.1 We need to target patients earlier

2.1.1 Patients presenting too late
2.1.1.1 Delayed referral to secondary care
2.1.1.1.1 We can't make a referral anyway
2.1.1.1.1.1 Secondary care referral thresholds are too high
2.1.1.1.1.1.1 Patients don't meet the criteria so have to wait until it gets worse
2.1.1.2 Waiting for secondary care to make the diagnosis
2.1.1.2.1 Support general practice to do some of the diagnostics
2.1.1.2.1.1 Lack of access to testing equipment in the community
2.1.1.2.1.2 Secondary care are gatekeepers for ABPIs and duplexes

2.2 Primary care needs to do more sooner

2.2.1 Adapt for use in general practice as part of a PAD prevention programme

For peer review only

2.2.1.1 Would promote earlier conversations with patients
2.2.1.2 Useful for managing patient expectations
2.2.1.3 Would support primary care to start best medical therapy
2.2.1.4 Could be used to provide additional information for referral to vascular team
2.2.1.4.1 Would support triage in secondary care
2.2.2 Use as an ongoing conversation guide
2.2.2.1 Existing relationships with patients will help to reinforce the message
2.2.2.2 Informing patients reduces the chance of presenting late or in crisis

Theme 3: Prioritising and making it happen

3.1 Prioritising alongside the burden of work
3.1.1 It shouldn't be seen as an extra thing
3.1.1.1 Previous experience of using intervention/checklists
3.1.1.2 Checklists are a quick quality check
3.1.1.3 Checklists are a useful memory prompt
3.1.1.4 Checklists are a threat to autonomy
3.1.1.5 Checklists are tick-box exercises rather than safety mechanisms
3.1.2 Confidence to implement guidelines
3.1.2.1 It's essentially what I always do
3.1.3 It takes time to build the relationship and have the conversation
3.1.3.1 Time to talk requires extra staff
3.1.3.2 Time burden of dictating long clinic letters
3.1.3.3 Limited access to blood patient monitoring and cholesterol tests in out-patient clinics
3.1.4 Impact of staff shortages
3.1.4.1 Reduced staffing due to covid
3.1.4.2 Reduced district nurse staffing for conducting ABPIs
3.1.5 Primary care is not an infinite resource
3.1.5.1 Long waiting times to be seen or to get tests
3.1.5.1.1 Sometimes it can take weeks to get them to answer a query over the phone
3.1.5.2 Sixty calls and forty letters a day
3.1.5.3 Patients don't always see the same GP
3.1.5.4 Championing the cause or doing the bare minimum and going home on time
3.1.5.4.1 Using research to demonstrate a difference

3.2 Working across primary and secondary care systems
3.2.1 Preventative medicine and health promotion is core to primary care practice
3.2.1.1 Existing systems in place for primary care medication management and monitoring
3.2.1.1.1 It can take weeks to get information on the system
3.2.1.1.2 Time delay between writing and receiving clinic letters
3.2.1.1.3 It can take up to three months to receive hospital letters
3.2.1.1.3.1 Provide 28 days medication to allow for delay in seeing GP
3.2.1.1.3.2 Patient difficulty ordering repeat medication
3.2.1.1.4 Avoid the extra step and order the test in secondary care
3.2.1.1.5 Secondary care to initiate smoking cessation referral
3.2.1.1.5.1 Local variation in community set-up of smoking cessation, dietary advice, exercise therapy support
3.2.1.1.5.1.1 No formal set-up in primary care to pick it up
3.2.1.1.5.1.2 Link with national protocol for weight reduction
3.2.1.1.6 It's not commissioned or financially viable
3.2.2 Provide a copy of the clinic/GP letter to the patient to reinforce shared messaging
3.2.2.1 Patients to hand deliver letters to general practice
3.2.3 Repeat GP action letter is a duplicate, adds to the workload, and sets the wrong tone
3.2.3.1 It gives the impression that secondary care do not trust GPs

3.3 Adapting the intervention to the environment
3.3.1 Fits the in-patient environment neatly
3.3.1.1 Incorporate into a universal admission/discharge bundle
3.3.1.1.1 Use at point of admission and discharge for in-patients
3.3.2 Use the intervention to support the outpatient clinic consultation
3.3.2.1 A record of the consultation conversation
3.3.2.2 A resource for referring back
3.3.3 Complexity in assessing patients remotely in the community

3.4 Training to support implementation
3.4.1 Teams are not static so require ongoing access to training
3.4.1.1 Prioritise junior staff – the people who do the day-to-day work
3.4.1.1.1 Consider including medical students
3.4.1.2 Written information for those unable to attend training
3.4.1.3 Webinar-ed out
3.4.2 Training delivered by vascular team to primary care
3.4.2.1 Involve all primary care staff and not just GPs
3.4.2.1.1 Highlight the link between PAD and cardiovascular death
3.4.2.1.2 Provide education on managing CLTI and non-CLTI patients in primary care
3.4.2.1.3 Taking an ABPI in primary care
3.4.2.1.4 Learning gaps in exercise and lifestyle modification
3.4.2.2 Visibility of vascular teams at primary care conferences
3.4.2.3 Use available supervision and de-briefing opportunities

Theme 4: Personalised information and supportive conversations for taking on the advice

4.1 Offer personalised care and tailor patient information to individual needs
4.1.1 Existing information does not suit all
4.1.1.1 Currently most likely to suit those with IC
4.1.1.2 Targets are impossible to reach
4.1.1.2.1 If I was younger and had more get-up-and-go
4.1.1.2.2 Availability of supervised exercise
4.1.1.2.3 Relying on others for food shopping
4.1.2 A different approach for each patient
4.1.3 Offer realistic targets or the advice rings hollow
4.1.4 Patient education leaflet is provided too late
4.1.4.1 Too far gone to talk about lifestyle modification
4.1.4.1.1 I can only do what I can do
4.1.4.2 Consider co-existing morbidities
4.1.4.3 Offer advice on moderating activity levels and pain management
4.1.4.4 Discuss concerns about medication side-effects

4.2 Everyone knows they need to stop smoking
4.2.1 Acknowledge and support patient preference and promote shared decision making
4.2.1.1 It's easy to bombard someone with information
4.2.1.2 Secondary care doctors tell people to do things
4.2.1.2.1 Consider the language and approach to engage patients
4.2.1.2.2 Be honest and explain the risks
4.2.1.2.2.1 Use available online tools to support the conversation
4.2.1.2.3 Use motivational interviewing techniques
4.2.1.2.4 Frank conversations for some
4.2.2 It's down to willpower at the end of the day
4.2.2.1 I like smoking, I like drinking, I like the telly

For peer review only

4.2.2.2 The only thing left is something nice to eat
4.2.2.2.1 Provide information on the benefits versus taking pleasure away
4.2.2.2.2 Strike a balance to optimise people's quality of life
4.2.2.2.2.1 You can't take everything away
4.2.3 Patient education leaflets are not the best way for everyone
4.2.3.1 I don't want any more leaflets
4.2.3.1.1 District nurses can answer my questions

For peer review only

COREQ checklist

Item number	Descriptor	Reported on page number
1	Interviewer/facilitator: Which author/s conducted the interview or focus group?	8
2	Credentials: What were the researcher's credentials? E.g. PhD, MD	8
3	Occupation: What was their occupation at the time of the study?	8
4	Gender: Was the researcher male or female?	
5	Experience and training: What experience or training did the researcher have?	8
6	Relationship established: Was a relationship established prior to study commencement?	N/A
7	Participant knowledge of the interviewer: What did the participants know about the researcher? E.g. personal goals, reasons for doing the research	N/A
8	Interviewer characteristics: What characteristics were reported about the interviewer/facilitator? E.g. bias, assumptions, reasons and interests in the research topic	8
9	Methodological orientation and theory: What methodological orientation was stated to underpin the study? E.g. grounded theory	6-7
10	Sampling: How were participants selected? E.g. purposive, theoretical	7
11	Method of approach: How were participants approached? E.g. face-to-face, telephone, email	7
12	Sample size: How many participants were in the study?	9
13	Non-participation: How many people refused to participate or dropped out? Reasons?	9-10
14	Setting of data collection: Where was the data collected? E.g. clinic, work place	8
15	Presence of non-participants: Was anyone else present besides the participants and researchers?	N/A
16	Description of sample: What are the important characteristics of the sample? E.g. demographic data, date	9
17	Interview guide: Were questions, prompts, guides provided by the authors? Was it pilot tested?	7-8, Data supplement
18	Repeat interviews: Were repeat interviews carried out? If yes, how many?	N/A
19	Audio/visual recording: Did the research use audio or visual recording to collect the data?	8
20	Field notes: Were field notes made during and/or after the interview or focus group?	8
21	Duration: What was the duration of the interviews or focus group?	8
22	Data saturation: Was data saturation discussed?	N/A
23	Transcripts returned: Were transcripts returned to participants for comment and/or correction?	N/A
24	Number of data coders: How many data coders coded the data?	8-9
25	Description of the coding tree: Did authors provide a description of the coding tree?	10-11, data supplement
26	Derivation of themes: Were themes identified in advance or derived from the data?	8
27	Software: What software was used to manage the data?	8
28	Participant checking: Did participants provide feedback on the findings?	N/A

For peer review only

29	Quotations presented: Were participant quotations presented to illustrate the themes/findings? Was each quotation identified? E.g. participant number	11-16
30	Data and findings consistent: Was there consistency between the data presented and the findings?	11-16 All findings illustrated with quotes
31	Clarity of major themes: Were major themes clearly presented in the findings?	11-16
32	Clarity of minor themes: Is there a description of diverse cases or discussion of minor themes?	11-16

Reference: Tong A, Sainsbury P, Craig J. Consolidated criteria for reporting qualitative research (COREQ): a 32-item checklist for interviews and focus groups. International Journal for Quality in Health Care. 2007; 19(6): 349-357.

For peer review only

Dear Editor 07/10/2022

Re: Factors that influence the feasibility and implementation of a complex intervention to improve the treatment of Peripheral Arterial Disease in primary and secondary care: a qualitative exploration of patient and provider perspectives.

Thank you very much for reviewing this manuscript. Please find responses to each point raised by the associate editor and reviewer(s) below. All changes to the manuscript are highlighted with track changes (all line numbers refer to the marked copy).

1. Please revise the title of your manuscript to include the research question, study design and setting. This is the preferred format of the journal.

   We have revised the title of the manuscript to the preferred format:

   > *Factors that influence the feasibility and implementation of a complex intervention to improve the treatment of Peripheral Arterial Disease in primary and secondary care: a qualitative exploration of patient and provider perspectives.*

2. Are there any frameworks or theory to guide this study? The MRC guidance is helpful to understand the implementation of a complex intervention. There are several indicators should be explored to understand the implementation not only acceptability but fidelity, sustainability and adoption.

   Thank you for this important point. We used the MRC framework to guide the research question and objectives of the study and reference Skivington et al., 2021. We have now made this explicit in our writing to add clarity (Lines 124-132):

   > *The aim of the qualitative study was to gain an understanding of the feasibility and implementation of the LEGS intervention, by exploring patient and provider perspectives on receiving and delivering the intervention (Skivington et al., 2021). Guided by the MRC framework for developing and evaluating complex interventions, the objectives of the qualitative study were to 1) to explore patient perspectives on receiving the LEGS intervention, 2) to gather secondary care clinician views on feasibility, and to understand factors that influence the success of implementation, and 3) to identify barriers and facilitators that influence the success of future implementation from the GP perspective.*

3. Some contextual factors such as health system and policy might also influence the implementation. It will be good to have a theme on these factors.

   We report (Lines 208-211) that we used existing implementation theory (the Consolidated Framework for Implementation Research (CFIR)) to facilitate analysis and to provide a layered coding approach. The CFIR provides an overarching typology (39 constructs) of implementation theory in terms of works where and why across multiple contexts, and this includes health system and policy influences (outer setting). We have added additional detail on the CFIR and explain that factors such as health system and policy might have an influence on implementation (called the outer setting) (Lines 264-271):

   > *The CFIR comprises five domains that provide an overarching typology of implementation theory (in terms of what works where and why): i) intervention*

For peer review only - http://bmjopen.bmj.com/site/about/guidelines.xhtml

> *characteristics (features of the intervention such as complexity), ii) outer setting (the external context that might influence implementation such as health policy and the extent to which patient needs, as well as barriers and facilitators to meet those needs are known and prioritised), iii) inner setting (features of the implementing organisation such as leadership, implementation readiness), iv) characteristics of individuals involved in implementation (such as beliefs and knowledge about the intervention), iv) implementation process…*
>
> *For outer setting, the specific needs of the patient group needs and barriers and facilitators to meet those needs were highlighted, with all participant groups explaining that a delayed referral to secondary care is a barrier to implementing the intervention. Staff participants went on to speak about external policies and incentives that might influence the spread and uptake of the intervention.*

The mapping of themes across CFIR domains and participant groups is also included in Online Data Supplement 2.

4. I recommend to revise the Results based on the objectives by using subheadings such as acceptability, barriers and facilitators.

   We approached the data inductively and used the CFIR to place our findings in the context of implementation theory. The CFIR is intended to be flexible in application so that researchers can tailor the framework to the specific intervention and context being studied. We used the CFIR headings to map our descriptive themes and have remained close to the data with theme derivation/names rather than imposing subheadings. This comprehensive approach to qualitative analysis has been commended as a strength of the study by other reviewers. We hope this clarifies our rationale for the choice of subheadings.

5. Since the theme titles are ambiguous, the subthemes will be more helpful to understand the results more in details, which are important to address the aims of this study. I recommend to move the characteristics of patients and clinicians to supplementary to save space.

   We have revised the text to explain that the content of the sub-themes is included in the paper by way of being used to summarise the overarching theme. They are not however labelled with separate headings in the text (Lines 307-309):

   > *Because of space limitations, a summary of each theme is provided with subthemes being used to define each overarching theme.*

6. Lines 120-122: the information you provide in the second half of the sentence regarding LEGS is redundant to the information that you provided in the entire previous paragraph. Please revise perhaps to "…feasibility and effectiveness of the LEGS intervention."

   Thank you for bringing this to our attention. We have made this change (Lines 122-124).

   > *CHABLIS is a multicentre prospective mixed-methods cohort study that aims to determine the feasibility and effectiveness of the LEGS intervention.*

For peer review only

7. Lines 124-128: your stated objectives don't clearly match up with the ones you provide in the abstract (acceptability and feasibility).

   Thank you for bringing this to our attention. We have amended the text (Lines 37-40):

   > *Our aim was to examine the feasibility and implementation of a complex intervention to improve the care of patients with Peripheral Arterial Disease (the LEGS intervention) from the perspective of patients, general practitioners and secondary care clinicians.*

8. Lines 132-133: the stated desired insights don't clearly (evidently, intuitively) match up with the objectives listed in lines 124-128 and the abstract. Consider starting more broad and then narrowing in on specific objectives to improve flow and congruence. I think that what would help once you decide upon the languaging of your aims is to define for the reader what you mean by, for example, feasibility or acceptability (or whatever your aims are).

   We have amended the text which now reads (Lines 136-138):

   > *The study used qualitative methods to provide insights into the feasibility and acceptability of the intervention as well as the complexities of implementation (Skivington et al., 2021).*

   In terms of languaging, we used the MRC framework to guide the research question and objectives of the study. We have now made this explicit in our writing to add clarity (Lines 124-132):

   > *The aim of the qualitative study was to gain an understanding of the feasibility and implementation of the LEGS intervention, by exploring patient and provider perspectives on receiving and delivering the intervention (Skivington et al., 2021). Guided by the MRC framework for developing and evaluating complex interventions, the objectives of the qualitative study were to 1) to explore patient perspectives on receiving the LEGS intervention, 2) to gather secondary care clinician views on feasibility, and to understand factors that influence the success of implementation, and 3) to identify barriers and facilitators that influence the success of future implementation from the GP perspective.*

9. Methods: Please state that you use the COREQ checklist to guide your reporting.

   We have included this information in Lines 142-144:

   > *The consolidated criteria for reporting qualitative research (COREQ) guideline was used to ensure quality when reporting the research (Tong, Sainsbury, &Craig,2007).*

10. Methods: Please state whether participants were compensated for taking part in the study

    We have now provided this detail in Line 182-183:

    > *Participants were not reimbursed for participating in the research.*

11. Lines 144-148: can you please clarify these stakeholders' role in the intervention? It is clear that the first two groups were directly involved with the intervention (patient/carer)

For peer review only

but I'm unclear on the relationship between these stakeholders and the intervention that was delivered…

We have provided additional information to aid clarity (Lines 148-151 and 153-157):

> *… semi-structured individual interviews were conducted with secondary care clinicians who were responsible for delivering (that is, delivering or intending to deliver) the intervention, including junior doctors, vascular nurses, and vascular surgeons.*

> *… semi-structured individual interviews were conducted with GPs to gain an understanding of the barriers and facilitators to future implementation in primary care and across the NHS. GPs were not involved in delivering the study intervention and were asked to report on perceived factors that might influence implementation beyond the context of the study.*

We also report in the results section (Lines 234-236) that:

> *Two of the eight secondary care clinicians were actively delivering the intervention (in an in-patient setting), and all GPs provided insights on future implementation.*

12. Lines 154-161: provide the number of patients who received the intervention (and thus made up your sampling frame). Same goes re: providing numbers for the number of 2ndary care providers and GPs eligible to participate. I think it makes sense to put these numbers here because you state "all eligible to participate were approached…"

    We have added details and provide the numbers as requested (Lines 169-180):

    > *All patients who had received the intervention and who gave consent for the researcher to make contact about the qualitative study were approached for participation (n=26). All secondary care clinicians responsible for delivering the intervention (n=13) were invited to take part. Secondary care clinicians were invited to participate following an in-person meeting with the local principal investigator at each site. A participant information sheet that outlined the study was provided to all potential participants with time to consider whether or not they would like to take part. GP participants were recruited from Clinical Commissioning Groups linked to the two secondary care implementation sites, chosen to enhance joined up working and to provide a local perspective on implementation across community and hospital settings. Potential GP participants (n=7) were first approached by the local Clinical Research Network research delivery manager or local contacts, and subsequently recruited by the lead author via telephone or email.*

13. Lines 157-161: how exactly were the secondary care clinicians invited (e.g., email or…)?

    We have added more detail (Lines 172-175):

    > *Secondary care clinicians were invited to participate following an in-person meeting with the local principal investigator at each site. A participant information sheet that outlined the study was provided to all potential participants with time to consider whether or not they would like to take part.*

For peer review only

14. Lines 150-168: it is unclear how you chose who you approached and when/how you decided to stop recruitment for each participant group… did you approach every eligible participant? Randomly select participants until you reached the desired number of participants, etc.

    We have added text to clarify (Lines 169-172):

    > *All patients who had received the intervention and who gave consent for the researcher to make contact about the qualitative study were approached for participation (n=26). All secondary care clinicians responsible for delivering the intervention (n=13) were invited to take part.*

15. Lines 172-174: I think it makes more sense to refer to supplement 1 (interview guide) in the preceding sentence as I initially thought that supplement 1 contained the interview schedule (based on where you refer to it).

    We have made this change (Lines 189-194):

    > *Interviews used flexible open-ended questions for early data collection to gather a rich and detailed understanding of participants' perspectives (included in Online Data Supplement 1). The patient and provider interview schedules were revised iteratively in response to the priorities and concerns of participants and gathered information on factors that facilitate or hinder implementation and on possible solutions to overcome any identified barriers.*

16. Data collection: Provide some high-level detail about the types of questions that were asked to save readers from having to go through the interview guides unless they want more in depth information.

    We have added this detail (Line 191-194):

    > *The patient and provider interview schedules were revised iteratively in response to the priorities and concerns of participants and gathered information on factors that facilitate or hinder implementation and on possible solutions to overcome any identified barriers.*

17. Lines 187: it is unclear what the (CL) refers to

    Thank you for bringing this to our attention. We have deleted the (CL).

18. Line 188-190: It would be nice if you expanded upon the CFIR framework in 1-2 sentences to provide further context for readers unfamiliar with it.

    Many thanks for this. We have added information to describe the CFIR (Lines 264-271):

    > *The CFIR comprises five domains that provide an overarching typology of implementation theory (in terms of what works where and why): i) intervention characteristics (features of the intervention such as complexity), ii) outer setting (the external context that might influence implementation such as health policy and the extent to which patient needs, as well as barriers and facilitators to meet those needs are known and prioritised), iii) inner setting (features of the implementing organisation such as leadership, implementation readiness), iv) characteristics of individuals involved in implementation (such as beliefs and knowledge about the intervention), iv) implementation process*

For peer review only

19. Data analysis – provide further detail (even just brackets with the initials) re: who carried out the steps.

We have clarified (Lines 218-220):

> *The lead author (CL) directed and conducted the analysis. Coding by a second rater (VL) was undertaken to provide an opportunity to reflect on the coding approach, and to enhance the interpretive depth of the data*

20. Line 197-198: expand upon what you mean by "in a way that supported data analysis" or consider stopping the sentence after "… manage the data."

We have amended the text and stopped the sentence after "…manage the data" (Line 216-217):

> *Data collection occurred concurrently with data analysis; NVivo QSR International qualitative analysis software (version 12) was used to manage the data.*

21. Lines 198-200: provide more detail about the coding that was done by the second rater as their role is unclear.

We have added more information to provide clarity (Lines 218-222):

> *Coding by a second analyst (VL) was undertaken to provide an opportunity to reflect on the coding approach, and to enhance the interpretive depth of the data (13). This involved an independent review of the data, the themes, and the CFIR mapping/tabulation. The overarching coding framework was developed following discussion with both analysts.*

22. Lines 202-205: consider using the IAP2 engagement spectrum or its adapted version to better describe PPI in developing the intervention and contributions to interpretation of study findings. Consider also providing a bit more information about the development sessions and also how the PPI co-applicant actually helped interpret the findings (via face-to-face meetings, providing written feedback on the themes, etc.).

Thank you for bringing this to our attention to this. We have used the research-relevant modified IAP2 spectrum to further describe the PPI contributions (Lines 225-229):

> *The LEGS intervention and CHABLIS mixed-methods study design were developed in collaboration with a stakeholder PPI group that included patients, family members and carers, and clinicians working in primary and secondary care (Bammer, 2019).*

We have removed the information about the development sessions and analysis as these are specific to developing the intervention – and not to the qualitative study – and will be reported elsewhere (paper in preparation).

23. Lines 229-242: This information should be contained in the first paragraph of the results and also summarized in a diagram that details the flow of participants into the study e.g., the number of eligible participants by group, number approached by group, number refused to participate (and reasons for refusal (if you have them) by group, and number agreeing to participate by group.

For peer review only

We have provided the number of potential participants in the methods for clarity (Lines 169-179). We have reported the number of people who declined to participate (and reasons for refusal, and the number agreeing to participate by group in the results section (Lines 250-256). We have not included a flow diagram of recruitment because our intention is to focus the paper on the experiences (rather than the numbers) of the included participants. We have discussed the limitations involved in recruiting patients with PAD in research in the discussion (Lines 562-572) which reads:

> *While the sample size for this study may seem small, the volume of interview data we collected is unique for this patient population. Many factors influence the success of recruiting participants in research and engaging vulnerable patient populations brings additional complexity. PAD is a chronic and progressive disease with burdensome symptoms including physical disability, cardiovascular risks and mortality. Those who live with PAD are vulnerable because they are living with extreme frailty, often with the threat or result of limb loss, and with risks associated with poorer socio-economic conditions. Alongside, patients with PAD might be less receptive to engaging with research because they experience specific barriers to accessing healthcare meaning that taking part in research alongside managing health care and health needs might be considered onerous.*

24. Line 229: remove (CL) as the information about who did the contacting belongs in the methods

    We have deleted the (CL).

25. Lines 229-231: report n/n (%) related to who agreed to participate… n/n (42%), n/n (82%).

    We have added this detail (Lines 250-252):

    > *Of the patient participants contacted by the researcher, (11/26) 42% agreed to take part in an interview (fifteen people declined) and (8/13) 82% of staff agreed to take part (1 person declined participation and 4 people did not respond to the invitation).*

26. Lines 235: The sentence "secondary care clinicians and GPS spoke…" should be a new paragraph as you are no longer describing the flow of participants into the study and should appear after the paragraph describing the participants. Give this paragraph a subheading to cue the reader that you've moved from describing the flow of participants into the study and participant characteristics to presentation of the themes.

    We have moved the text to a new paragraph and provided a sub-heading: Using the CFIR to facilitate analysis (Line 263).

27. Lines 235-242: I find this information confusing. Likely partially because of it being tucked in with the "flow of participants" info and maybe due to the lack of explanation about the CFIR in the methods. I don't have a concrete suggestion around how to clarify what's here but it does need revision. Perhaps including (and not limited to) some added detail in the methods that better sets the reader's expectations and understanding around the information presented here. You definitely need to provide some explanation of what the CFIR domains mean as this understanding is foundational to understanding what you present in the rest of your results.

For peer review only

We have added more detail on the CFIR, the CFIR domains and our rationale for using the framework (Lines 263-304):

> ***Using the CFIR to facilitate analysis***
>
> *The CFIR comprises five domains that provide an overarching typology of implementation theory (in terms of what works where and why): i) intervention characteristics (features of the intervention such as complexity), ii) outer setting (the external context that might influence implementation such as health policy and the extent to which patient needs, as well as barriers and facilitators to meet those needs are known and prioritised), iii) inner setting (features of the implementing organisation such as leadership, implementation readiness), iv) characteristics of individuals involved in implementation (such as beliefs and knowledge about the intervention), iv) implementation process (Damschroder et al., 2009). The themes map to four of the five overarching CFIR domains across participant groups: intervention characteristics, outer setting, inner setting, and characteristics of individuals. In summary, for intervention characteristics, all participant groups spoke about the advantage of implementing the care bundle, provided insights on improvements to the design quality and packaging, and acknowledged the need to adapt and tailor the intervention to meet the needs of individuals. Complexity was identified in terms of implementing the intervention across the primary-secondary care interface. For outer setting, the specific needs of the patient group needs and barriers and facilitators to meet those needs were highlighted, with all participant groups explaining that a delayed referral to secondary care is a barrier to implementing the intervention. Staff participants went on to speak about external policies and incentives that might influence the spread and uptake of the intervention. For inner setting, the structural characteristics and the quality of networks and communications within and across each organisation were identified by all groups as influences on implementation. For structural characteristics, participants explained that the ability to follow the advice in the education leaflet is often determined by the availability of supervised exercise programmes, difficulty ordering medication, and relying on carers for food shopping. For networks and communications, participants spoke about the need to work as a three-way team, and to have ongoing access to the specialist vascular team. For characteristics of individuals, staff values, attitude, knowledge, and skills to provide the intervention and patient readiness to take on the advice of the intervention were highlighted across participant groups. The patient-healthcare provider relationship was identified as a central influence on implementation and there was a particular emphasis on individual stage of change, with staff participants highlighting a need to engage patients in their care and to promote shared decision making. The mapping of themes across CFIR domains and participant groups is included in Online Data Supplement 2.*

28. Lines 244-262: It would be nice if you could create a figure that summarizes this info and is embedded in the manuscript (instead of a supplement). It will help the reader better absorb findings you present here.

    We attempted to create a figure to illustrate the overarching themes but there is complexity in that the CFIR was used as a part of the process - to provide a layered approach to coding (by mapping descriptive themes to implementation theory). The overarching themes are the final result of that process and an additional level of interpretation. The interpretive analysis allowed for the refining of the specifics of

For peer review only - http://bmjopen.bmj.com/site/about/guidelines.xhtml

themes and thematic patterns generated by the data, and so the CFIR domains are integrated within them and do not map directly to any one theme.

We describe the coding process in Lines 203-214:

> *Data analysis began with repeated re-reading of individual transcripts and re-listening of sound files for data immersion. This was followed by line-by-line open coding, where extracts were coded under one or several descriptive themes, that is organising the data according to semantic content, to capture their meaning and reflect the content of the data. Each theme was refined, and where data allowed, further sub-themes were developed. The Consolidated Framework for Implementation Research (CFIR) was then used as a matrix to organise the early themes, to explore patterns and relationships in the data, and to map connections between the themes and the three participant groups. Lastly, interpretive analysis allowed for the refining of the specifics of themes and thematic patterns, and a final coding frame for patients and providers was developed. Refinements to the specifics of themes, and thematic patterns continued until a useful and meaningful analysis was achieved.*

29. Lines 450: Ensure the aim you report here is consistent with your revision based on my initial comments re: fluctuating aims.

Many thanks. We have provided clarity and ensured the aim is consistent with our earlier revision (Lines 494-497):

> *The aim of the study was to provide an understanding of the feasibility and implementation of the LEGS (LEaflet Gp letter Structured checklist) intervention, a complex intervention in the form of a healthcare bundle, designed to improve the care of patients with PAD in the NHS.*

30. Lines 460-461: Your use of moreover makes this read as an incomplete sentence. Consider merging this paragraph's first two sentences since I think your intention is to list what all participants spoke positively about.

Many thanks for highlighting this. We have amended the text to read (Lines 504-506):

> *All participants spoke positively about using the intervention to make a difference to patient care, as an opportunity to meet patient needs, and as a way to enhance the quality of PAD care through improved working practices.*

31. Discussion: I really like your focus on the lessons learned and next steps in your discussion, but I think you are missing a paragraph that explores how your findings fit in with the broader literature.

We have added further detail on how the findings fit with the broader literature to the discussion (Lines 500-526):

> *We used the Consolidated Framework of Implementation Research (CFIR) to guide our analysis, and all five CFIR domains are represented (Damschroder et al., 2009). The CFIR is well-placed for developing a model for implementing secondary care-initiated treatment in primary care (Le Boutillier et al., 2022). All participants spoke positively about using the intervention to make a difference to patient care, as an opportunity to meet patient needs, and as a way to enhance the quality of PAD care through improved working practices. This promotes a streamlined patient pathway from primary care to secondary*

For peer review only

care and to shared care. This reflects the value of an agreed pathway of care and shared partnership working that provides an opportunity for the intervention to be delivered as part of a wider team and to prioritise patient-centred care (Le Boutillier et al., 2022).

There was a consensus of optimism in participant accounts about the potential to make a difference. While barriers to seeking care for those with PAD are well-documented (Tan et al., 2022; Meffen et al., 2021), the findings confirm that the period between diagnosis of early-stage PAD (e.g., claudication) and subsequent potential diagnosis of advanced PAD with severe symptoms, is a time of no man's land, when patients do not receive any help, education, support, or appropriate medication to address their condition (Saratzis et al., 2019). As such, the benefits of the LEGS intervention were identified in terms of meeting the needs of this subgroup of patients, and to address the gap in care. GPs were optimistic that they can deliver the LEGS intervention with training and ongoing sustained communication with secondary care. The findings highlight the need to consider training requirements and systems for maintaining open primary-secondary care communication to support implementation. For example, education on tailoring the intervention for patients at both early and late stages of PAD, that can be used across primary and secondary care settings, similar to the model used with other chronic conditions (Smith et al., 2017; Le Boutillier et al., 2022). Improving the awareness of PAD among healthcare providers and supporting patients to take responsibility for their own health are therefore important determinants in the success of implementation (Bridgwood et al., 2020).

32. Line 506: PAD not Peripheral Arterial Disease

    We have made this change (now Line 558)

33. Strengths and Limitations: I usually place these before my conclusions. I'm unsure if BMJ Open has a preference for this ordering.

    Many thanks. We have made this change.

We have also updated our author affiliations. We hope that these responses and improvements make the paper suitable for publication in BMJ Open.

Yours faithfully,

Clair Le Boutillier

For peer review only

Title: **Factors that influence the feasibility and implementation of a complex intervention to improve the treatment of Peripheral Arterial Disease in primary and secondary care: a qualitative exploration of patient and provider perspectives**

Authors: Clair Le Boutillier[1,2], Athanasios Saratzis[3], Prakash Saha[4], Ruth Benson[5], Bernadeta Bridgwood[6], Emma Watson[3,6], Vanessa Lawrence*[1]

[1] Institute of Psychiatry, Psychology & Neuroscience, King's College London, UK
[2] Florence Nightingale Faculty of Nursing, Midwifery & Palliative Care, King's College London, UK
[3] University of Leicester, UK
[4]Guy's and St Thomas' NHS Foundation Trust, UK
[5]University of Birmingham, UK
[6]NIHR Cardiovascular Sciences, University of Leicester, Leicester, UK.

* Corresponding authors. Email: vanessa.c.lawrence@kcl.ac.uk; clair.le_boutillier@kcl.ac.uk

Division of Methodologies, Florence Nightingale Faculty of Nursing, Midwifery & Palliative Care, King's College London, James Clerk Maxwell Building, 57 Waterloo Road, London SE1 8WA. Orcid iD: 0000-0003-3665-0315

Funding

The study was funded by the National Institute for Health Research (NIHR) – Research for Patient Benefit (RfPB) Programme (Reference: NIHR202008).

Category

Original article

For peer review only - http://bmjopen.bmj.com/site/about/guidelines.xhtml

For peer review only

Abstract

Objectives: Our aim was to examine the ~~acceptability and~~ feasibility and implementation of a complex intervention to improve the care of patients with Peripheral Arterial Disease (the LEGS intervention) from the perspective of patients, general practitioners and secondary care clinicians.

Design: A qualitative study involving semi-structured individual interviews with patients and providers to gain an understanding of the feasibility ~~acceptability, usability, and relevance~~ of the LEGS intervention as well the barriers and facilitators to implementation in secondary and primary care.

Setting: Primary and secondary care settings across two NHS Trusts.

Participants: Twenty-five semi-structured telephone interviews were conducted with i) patients who had received the intervention (n=11), ii) secondary care clinicians responsible for delivering the intervention (n=8), and iii) general practitioners (n=6).

Analysis Data were initially analysed using inductive descriptive thematic analysis. The consolidated framework for implementation research was then used as a matrix to explore patterns in the data and to map connections between the three participant groups. Lastly, interpretive analysis allowed for refining, and a final coding frame was developed.

Results: Four overarching themes were identified: i) The potential to make a difference, ii) A solution to address the gap in no man's land, iii), Prioritising and making it happen and iv) Personalised information and supportive conversations for taking on the advice. The impetus for prioritising and delivering the intervention was further driven by its flexibility and adaptability to be tailored to the individual and to the environment.

Conclusions: The LEGS intervention can be tailored for use at early and late stages of Peripheral Arterial Disease, provides an opportunity to meet patient needs and can be used to promote shared working across the primary-secondary care interface.

For peer review only

Strengths and limitations

- A strength of this study is the focus on rigor and triangulation of participant perspectives.
- We used implementation theory to guide analysis and to provide a layered coding approach, to ensure that intervention and implementation recommendations are novel, grounded in the data, and useful.
- While the anonymity of telephone use can allow participants to disclose sensitive information, telephone interviews have received criticism for compromising interviewer/participant rapport and interaction, and for limiting contextual data due to the absence of face-to-face contact and visual cues. However, this method of data collection is convenient, in that it is flexible (in terms of time and location), accessible (i.e., remote research conducted during the COVID-19 pandemic), and allows for a wide reach (e.g., accessing a diverse population from both urban and rural areas).
- Although the sample size for this study may seem small, the volume of interview data we collected is unique for this patient population. Living with the symptoms of Peripheral Arterial Disease is challenging, and patients also experience barriers to accessing healthcare. Engaging in research alongside managing these specific health care and health needs can be burdensome.

List of abbreviations

PAD	Peripheral Arterial Disease
IC	Intermittent Claudication
CLTI	Chronic Limb Threatening Ischaemia
GP	General Practitioner
CHABLIS	Community and Hospital cAre Bundle to improve the medical treatment of cLaudIcation and critical limb iSchaemia
LEGS	LEaflet Gp letter Structured checklist
UHL	University Hospitals of Leicester NHS Trust
GSTT	Guy’s and St Thomas’ NHS Foundation Trust
CFIR	The consolidated framework for implementation research
NHS	National Health Service

For peer review only - http://bmjopen.bmj.com/site/about/guidelines.xhtml

Background

Peripheral Arterial Disease (PAD) affects a fifth of people over the age of sixty in the United Kingdom. This prevalence is also expected to rise due to sedentary lifestyles, poor dietary choices, a longer life-expectancy, and rising numbers of those with diabetes (1, 2). Patients with PAD who develop symptoms present either with Intermittent Claudication (IC) or in some cases, Chronic Limb Threatening Ischaemia (CLTI) where the reduction in blood flow is so severe that it causes pain at rest, ulceration, or gangrene (3). Patients with IC are more likely to suffer a cardiovascular event compared to age-matched individuals, and the one-year risk of limb amputation for those with CLTI is 30%. More than half of those diagnosed with symptomatic PAD are expected to die, have an amputation or a major cardiovascular event within five years (4-7).

The treatment of these high-risk individuals is shared between primary and secondary care. While established national and international guidance (8) outlines how PAD risk-factors should be addressed to prevent cardiac events and amputations, a study conducted by our group found that only a tenth of those diagnosed with PAD were prescribed appropriate preventive medication, and almost none received structured lifestyle or dietary support (9). As a result, and following patient and public engagement (that is, interviews and workshops with patients and a national consultation with primary and secondary care clinicians) and a review of the existing evidence, a complex intervention in the form of a care bundle was developed to support patients, General Practitioners (GPs), and secondary care clinicians to actively manage cardiovascular risk-factors, and to improve implementation of existing guidance when treating patients with PAD. The care bundle, called the LEGS intervention (Leaflet, Gp letter, Structured checklist) has three elements: i) a one-page clinical checklist for the ward and a separate one-page clinical checklist for the secondary care clinic, ii) a patient and carer education leaflet (developed with patients), and iii) a GP action letter, that can be sent to GPs at regular intervals to inform them of the PAD diagnosis and corresponding practice guidance.

For peer review only - http://bmjopen.bmj.com/site/about/guidelines.xhtml

The LEGS intervention and full study protocol are available online (isrctn.com/ISRCTN13202085).

This qualitative study was embedded within the Community and Hospital cAre Bundle to improve the medical treatment of cLaudIcation and critical limb iSchaemia (CHABLIS) study. CHABLIS is a multicentre prospective mixed-methods cohort study that aims to determine the feasibility and effectiveness of ~~using a PAD care bundle, called~~ the LEGS intervention. ~~(LEaflet Gp letter Structured checklist).~~ ~~T~~The aim of the qualitative study was to gain an understanding of the feasibility and implementation ~~acceptability~~ of the LEGS intervention, by exploring patient and provider perspectives on receiving and delivering the intervention (10). Guided by the MRC framework for developing and evaluating complex interventions, t~~T~~he objectives of the qualitative study were: 1) to explore patient perspectives on receiving the LEGS intervention, 2) to gather secondary care clinician views on feasibility, ~~acceptability and usability,~~ and to understand factors that influence the success of implementation, and 3) to identify barriers and facilitators that influence the success of future implementation from the GP perspective.

METHODS

Study design

The study used qualitative methods to provide insights into the feasibility and acceptability~~, usability, and relevance~~ of the intervention as well as the complexities of implementation (10). Semi-structured individual interviews were conducted with patients and providers to gain an understanding of the barriers and facilitators to implementation in secondary and primary care. The research was reviewed and approved by the NHS Wales Research Ethics Committee (REC), reference number20/WA/0319 and the NHS Health Research Authority (HRA). The consolidated criteria for reporting qualitative

For peer review only

For peer review only - http://bmjopen.bmj.com/site/about/guidelines.xhtml

research (COREQ) guideline was used to ensure quality when reporting the research (11).

To meet objective 1, semi-structured individual interviews were conducted with patients with symptomatic PAD (i.e., IC, ischaemic rest pain, or CLTI), who had received the intervention (either in an outpatient clinic or as a part of inpatient treatment). To meet objective 2, semi-structured individual interviews were conducted with secondary care clinicians who were responsible for delivering (that is, delivering or intending to deliver) the intervention, including junior doctors, vascular nurses, and vascular surgeons. Staff and patients were recruited from two implementation sites: University Hospitals of Leicester NHS Trust (UHL) and Guy's and St Thomas' NHS Foundation Trust (GSTT). To meet objective 3, semi-structured individual interviews were conducted with GPs to gain an understanding of the barriers and facilitators to future implementation in primary care and across the NHS. GPs were not involved in delivering the study intervention and were asked to report on perceived factors that might influence implementation beyond the context of the study.

~~The research was reviewed and approved by the NHS Wales Research Ethics Committee (REC), reference number20/WA/0319 and the NHS Health Research Authority (HRA).~~

Participants were selected purposively (both those who were receiving or responsible for delivering the LEGS intervention) on the basis that they are the target adopters of behaviours that influence the success of future implementation and/or could offer a particular perspective on implementation. ~~Participants were over 18, and proficient in English. All patients who received the intervention between June and October 2021, were eligible to take part. Eligible patients were approached and recruited by the local research team (for example, local principal investigator, research nurse), via face-to-face consultation as a part of outpatient clinic or hospital admission.~~ All patients who had received the intervention and who gave

For peer review only

For peer review only - http://bmjopen.bmj.com/site/about/guidelines.xhtml

consent for the researcher to make contact about the qualitative study were approached for participation (n=26). All secondary care clinicians responsible for delivering the intervention (n=13) were invited to take part. ~~by the local research team.~~ Secondary care clinicians were invited to participate following an in-person meeting with the local principal investigator at each site. A participant information sheet that outlined the study was provided to all potential participants with time to consider whether or not they would like to take part. GP participants were recruited from Clinical Commissioning Groups linked to the two secondary care implementation sites, chosen to enhance joined up working and to provide a local perspective on implementation across community and hospital settings. Potential GP participants (n=7) were first approached by the local Clinical Research Network research delivery manager or local contacts, and subsequently recruited by the lead author via telephone or email. Written or verbal informed consent was obtained from each participant after a full explanation and information leaflet was given and time allowed for consideration. Participants were not reimbursed for participating in the research. The right of each participant to refuse to participate without giving reasons was respected. All participants were free to withdraw from the study at any time without giving reasons and without prejudicing further treatment or employment.

For peer review only

Data collection

Interviews used flexible open-ended questions for early data collection to gather a rich and detailed understanding of participants' perspectives (included in Online Data Supplement 1). The patient and provider interview schedules were revised iteratively in response to the priorities and concerns of participants ~~and are included in Online Data Supplement 1.~~ and gathered information on factors that facilitate or hinder implementation and on possible solutions to overcome any identified barriers. Interviews were conducted by telephone by the lead author (a qualitative research fellow), lasted around 45 minutes, were audio-recorded and transcribed verbatim. Researcher reflexive notes were kept after each interview to

consider the interaction with the participant, and to detail initial thoughts. Interviews were conducted between September and November 2021.

Data Analysis

Data from the three participant groups (patients, secondary care clinicians and GPs) were first analysed separately using inductive thematic analysis, where analytical concepts and perspectives are generated from the data in a deliberate and systematic way (12). Data analysis began with repeated re-reading of individual transcripts and re-listening of sound files for data immersion. This was followed by line-by-line open coding, where extracts were coded under one or several descriptive themes, that is organising the data according to semantic content, to capture their meaning and reflect the content of the data. Each theme was refined, and where data allowed, further sub-themes were developed~~ (CL)~~. The Consolidated Framework for Implementation Research (CFIR) was then used as a matrix to organise the early themes, to explore patterns and relationships in the data, and to map connections between the themes and the three participant groups (13). Lastly, interpretive analysis allowed for the refining of the specifics of themes and thematic patterns, and a final coding frame for patients and providers was developed. Refinements to the specifics of themes, and thematic patterns continued until a useful and meaningful analysis was achieved (14).

Data collection occurred concurrently with data analysis; NVivo QSR International qualitative analysis software (version 12) was used to manage the data. ~~in a way that supported data analysis~~ (15). The lead author (CL) directed and conducted the analysis. Coding by a second analyst ~~rater~~ (VL) was undertaken to provide an opportunity to reflect on the coding approach, and to enhance the interpretive depth of the data (14). This involved an independent review of the data, the themes, and the CFIR mapping/tabulation. The overarching coding framework was developed following discussion with both analysts.

For peer review only

For peer review only - http://bmjopen.bmj.com/site/about/guidelines.xhtml

Patient and public involvement

The LEGS intervention and CHABLIS mixed-methods study design were developed in collaboration with a stakeholder PPI group that included ~~PPI development sessions with eight~~ patients, family members and carers ~~with PAD~~, and clinicians working in primary and secondary care. ~~. Ongoing PPI support was given by a PPI co-applicant who helped interpret the qualitative findings~~ (16).

RESULTS

Participants

A total of 25 individual interviews were conducted with i) patients who had received the intervention (n=11), ii) secondary care clinicians responsible for delivering the intervention (n=8), and iii) GPs (n=6). Two of the eight secondary care clinicians were actively delivering the intervention (in an in-patient setting), and all GPs provided insights on future implementation. Six patients had received the intervention as a part of in-patient care and five patients provided perspectives on receiving the intervention from out-patient vascular, ulcer, or specialised CLTI clinics. Patient participants spoke about living with multiple conditions including diabetes, stroke, and amputation and receiving care from primary care (GPs, district nurses, diabetic nurses) secondary care (vascular teams), social care (carers), and family. Three patients were >70 years old but all patients were retired or unemployed and spoke about the impact of PAD on quality of life including managing pain, slow wound healing, poor sleep, reduced daily functioning, and limited mobility. Patient characteristics are shown in Table 1 and provider characteristics are detailed Table 2.

Table 1: Patient participant characteristics

**Patient characteristics** n (%)	Patients n=11

For peer review only

**Age**	
41-50 years	1 (9.1)
51-60 years	0 (0.00)
61-70 years	2 (18.2)
>70 years	8 (72.7)
**Gender**	
Male	8 (72.7)
Female	3 (27.3)
**Ethnicity**	
White British	9 (81.8)
White Irish	1 (9.1)
Black/Black British-Caribbean	1 (9.1)
**Marital status**	
Single	2 (18.2)
Married	7 (63.6)
Widowed	2 (18.2)
**Time since diagnosis**	
>1 year	3 (27.3)
1-4 years	4 (36.35)
5-9 years	0 (0.00)
10 years +	4 (36.35)
**No. of surgical interventions**	
None	1
Angioplasty	8
Bypass graft	4
Amputation	2

247

248 **Table 2**: **Provider participant characteristics**

**Provider characteristics** n (%)	Hospital staff n=8	GPs n=6
**Age**		
21-30 years	3 (37.5)	1 (16.7)
31-40 years	5 (62.5)	3 (50.0)
41-50 years	0 (00.0)	2 (33.3)
**Gender**		
Male	4 (50.0)	4 (66.7)
Female	4 (50.0)	2 (33.3)
**Ethnicity**		
White British	2(25.0)	3 (50.0)
White Other	1 (12.5)	1 (16.7)
Asian/Asian British-Indian	2 (25.0)	2 (33.3)
Mixed. White & Asian	1 (12.5)	0 (00.0)
Chinese	1 (12.5)	0 (00.0)
Other	1 (12.5)	0 (00.0)
**Core Profession**		
Nurse	1 (12.5)	0 (00.0)
Vascular surgeon	7 (87.5)	0 (00.0)
GP trainee	0 (00.0)	1 (16.7)
General practitioner	0 (00.0)	5 (83.3)

For peer review only - http://bmjopen.bmj.com/site/about/guidelines.xhtml

**Grade**		
Band 8a	1 (12.5)	0 (00.0)
Senior House Officer	2 (25.0)	0 (00.0)
Specialty trainee	1 (12.5)	1 (16.7)
Speciality registrar	2 (25.0)	0 (00.0)
Consultant	2 (25.0)	0 (00.0)
GP	0 (00.0)	5 (83.3)
**Time since qualification**		
0-2 years	0 (00.0)	1 (16.7)
2 years + -5 years	0 (00.0)	1 (16.7)
5 years + -10 years	3 (37.5)	2 (33.3)
10 years + -15 years	5 (62.5)	2 (33.3)
**Time in current post**		
>6 months	5 (62.5)	1 (16.7)
7-12 months	0 (00.0)	0 (00.0)
13-24 months	2 (25.0)	2 (33.3)
25-36 months	0 (00.0)	1 (16.7)
37-48 months	1 (12.5)	0 (00.0)
49-60 months	0 (00.0)	0 (00.0)
61-75 months	0 (00.0)	1 (16.7)
13 years	0 (00.0)	1 (16.7)

Of the patient participants contacted by the researcher ~~(CL)~~, (11/26) 42% agreed to take part in an interview (fifteen people declined) and (8/13) 82% of staff agreed to take part (1 person declined participation and 4 people did not respond to the invitation). Reasons for non-participation for patients included being unable to remember the intervention (n=5), deteriorating health (n=7), just home from an in-patient admission (n=1), being admitted for angioplasty (n=1) and getting back on with life (n=1). The reason provided by providers for declining participation was moving to a new role and lack of time.

Secondary care clinicians and GPs spoke about all three elements of the intervention and patients provided insights on the education leaflet and GP action letter. The descriptive themes were mapped to implementation theory using the CFIR, to explore connections between the themes, and to compare findings across participant groups.

Using the CFIR to facilitate analysis

The CFIR comprises five domains that provide an overarching typology of implementation theory (in terms of what works where and why): i) intervention characteristics (features of the

For peer review only

intervention such as complexity), ii) outer setting (the external context that might influence implementation such as health policy and the extent to which patient needs, as well as barriers and facilitators to meet those needs are known and prioritised), iii) inner setting (features of the implementing organisation such as leadership, implementation readiness), iv) characteristics of individuals involved in implementation (such as beliefs and knowledge about the intervention), iv) implementation process (Damschroder et al., 2009). The themes map to all f~~Four of the~~ five overarching CFIR domains ~~are represented~~ across participant groups: intervention characteristics, outer setting, inner setting, ~~and~~ characteristics of individuals and process. In summary, for ~~The mapping of themes across CFIR domains and participant groups is included in Online Data Supplement 2.~~ intervention characteristics, all participant groups spoke about the ~~relative~~ advantage of implementing the care bundle, provided insights on improvements to the design quality and packaging, and acknowledged the need to adapt and tailor the intervention to meet the needs of individuals. Complexity was identified in terms of implementing the intervention across the primary-secondary care interface.

~~, adaptability, and design, quality, and packaging of the education leaflet, as well as the complexity in implementing the GP action letter.~~

For outer setting, the specific needs of the patient group and ~~needs and resources (including barriers and facilitators to meet~~ those needs ~~)~~ were highlighted, with all participant groups explaining that a delayed referral to secondary care is a barrier to implementing the intervention. Staff participants went on to speak about external policies and incentives that might influence the spread and uptake of the intervention. For inner setting, the structural characteristics and the quality of networks and communications within and across each organisation were identified by all groups as influences on implementation. For structural characteristics, participants explained that the ability to follow the advice in the education leaflet is often determined by the availability of supervised exercise programmes, difficulty ordering medication, and relying on carers for food shopping. For networks and communications, participants spoke about the need to work as a three-way team, and to

For peer review only - http://bmjopen.bmj.com/site/about/guidelines.xhtml

have ongoing access to the specialist vascular team. For characteristics of individuals, staff values, attitude, knowledge, and skills to provide the intervention and patient readiness to take on the advice of the intervention were highlighted across participant groups. The patient-healthcare provider relationship was identified as a central influence on implementation and t~~T~~here was a particular emphasis on ~~the~~ individual stage of change ~~sub-domain~~, with staff participants highlighting a need to engage patients in their care and to promote shared decision making. ~~The patient-healthcare provider relationship was identified as a central influence relating to individual identification within the organisation, that is, how patients and staff perceive the organisation and their relationship with that organisation).~~ The mapping of themes across CFIR domains and participant groups is included in Online Data Supplement 2.

A final coding frame for patients, secondary care clinicians and GPs was developed following interpretive analysis. Because of space limitations, a summary of each theme is provided ~~summary of each theme is provided~~ with subthemes being used to define each overarching theme;~~, and the subthemes are not elaborated in this article;~~ the full coding framework is included in Online Data Supplement 3. Four overarching themes were identified: *the potential to make a difference, a solution to address the gap in no man's land, prioritising and making it happen* and *personalised information and supportive conversations for taking on the advice.* Similarities and differences between participant groups are presented where they arise.

Theme 1: The potential to make a difference

GPs and secondary care clinicians felt that the LEGS intervention provides an opportunity to meet patient need and to support implementation of NICE guidance and spoke optimistically about using the intervention to improve patient outcomes and to standardise a minimum quality of care.

For peer review only

> *…it standardises the quality of care and standardisation is a big problem that we have in vascular surgery because guidance is so vague. So, I think it raises awareness and standardises that at least we ensure the patient gets a minimum of care* (QR15, secondary care clinician, UHL).

GPs and secondary care clinicians went on to explain how the intervention can be used to inform, empower, and support relationship building with patients.

> *I've gone through the leaflet with them… and knowing that the patient has understood I will spend a couple of minutes going through the leaflet to make sure. If they've got any questions I will try and take the opportunity to build my relationship with the patient so that they do understand* (QR08, secondary care clinician, UHL).

To further improve quality of care, providers identified the intervention as a way to support shared working across primary and secondary care, promoting a team approach and providing an opportunity for collaborative working. One GP explained how the '*patient is in the middle'* (QR23) and a secondary care clinician felt that '*we don't sing off the same hymn sheet'* (QR08). One GP explained that we *don't ever see secondary care specialists* and a secondary care clinician explained *there's always this assumption that the GP will sort it out* (QR13). It was acknowledged that shared working provides an opportunity to understand each other's roles, to manage expectations and to learn from one another. Providers went on to report patient benefits in terms of access to specialist clinicians and working as a multi-disciplinary team, *I've always found that patients who have the opportunity to talk to multiple members of the medical team rather than a doctor or a nurse come out with better outcomes* (QR08, secondary care clinician, UHL).

Patients also spoke about the benefit of joined-up working, with one person stating;

> *…you attend lots of different clinics, you've got different people involved in your healthcare. It's not just one thing and everything's linked… it's what they call whole person care. It's not just your blood pressure, it's also the vascular team, it's the podiatrist, it's the diabetes specialist* (QR07, out-patient clinic, GSTT).

Theme 2: A solution to address the gap in no man's land

All participant groups spoke about a need to target patients earlier and identified a service provision gap in preventative medicine and early diagnosis. One secondary care clinician stated;

For peer review only - http://bmjopen.bmj.com/site/about/guidelines.xhtml

> *I think, almost targeting people earlier on, so claudicants would be really good… claudicants at the very first stage of their peripheral arterial disease, they're not really managed in secondary care, but they're also not really managed in primary care… this gap where they're in no man's land and they almost wait until it's severe enough to managed into secondary care* (QR18, secondary care clinician, UHL).

Patients explained how it took time to be referred to secondary care, and in one person's situation, it became an emergency;

> *…my doctor prescribed pills and some cream for my foot but they didn't do anything. And then it was only when I went back to podiatry after three months of constant pain, it was a case of just wait and see how things went… I went one day in the November, and they said you need to have your toe off almost immediately… they sent me to [hospital] and [hospital] say 'your toe needs to come off and it needs to come off in 20 minutes time'* (QR04, In-patient care, UHL).

Difficulties with patients presenting late, high secondary care referral thresholds resulting in delayed referrals to secondary care and a wait for secondary care to make the diagnosis, alongside lack of support for primary care to commence diagnostics in the community were also listed as barriers to accessing timely care. Some secondary care clinicians felt that the care of those with IC should be managed in primary care.

> *I think even the way that if the GP could start the patient, the first medical therapies, like, clopidogrel and statins, if he also could start the patient on exercise therapies. So, it should be implemented at the same kind of wave of the first medical therapy.* (QR19, secondary care clinician, UHL).

GPs offered their support and made a request to do more sooner. Some suggested using the intervention in general practice as part of a PAD prevention programme. They acknowledged the value of using the intervention to promote earlier conversations with patients, manage patient expectations, and support primary care to start best medical therapy. GPs also spoke about how being involved sooner, and completing the clinical checklist, could be used to support triage of referrals to secondary care. GPs noted that preventative medicine and health promotion is core to their practice, and that sharing information across services and with patients reinforces the message and supports implementation.

Theme 3: Prioritising and making it happen

Secondary care clinicians spoke about the need to prioritise the intervention alongside limited resources and the burden of work. One secondary care clinician explained, *it shouldn't be*

For peer review only - http://bmjopen.bmj.com/site/about/guidelines.xhtml

seen as an extra thing (QR08). Individual levels of confidence, attitude, knowledge and experience of the LEGS bundle, or a similar checklist/patient education intervention influenced prioritisation with some staff identifying checklists as a quick and easy-to-use memory prompt. However, another reported that;

> *…people feel that it's just a tick-box exercise rather than a safety mechanism, and therefore no engagement with it… [it's] another piece of paper… I think part of the challenge is engagement with clinicians. Clinicians don't like threats to their autonomy… doctors, surgeons in particular are reticent, or certainly some are reticent to engage with checklists because they feel that it is doubling down or they already know this or that, you know, this simplifies things* (QR13, secondary care clinician, UHL).

Secondary care clinicians spoke about the extra resources required to deliver the intervention; it takes time to build relationships and to have conversations with patients. One secondary care clinician stated that finding the time to talk would require extra staff, this in an already stretched environment with staff shortages and long waiting times.

> *That will take more time, to make it personalised, to make it meaningful… you could almost imagine that they have a consultation and then some of those patients might benefit from sitting down with one of the specialist nurses… you could almost benefit from a 20-minute appointment after the standard clinic appointment to go through all of that. But again, that's a resource burden, that means that you've got to have someone extra in clinic* (QR13, secondary care clinician, UHL).

Secondary care clinicians felt that the intervention fits the in-patient environment neatly and could be used as a record of the out-patient clinic consultation. GPs felt that it could support the complexity of assessing patients remotely in the community. The need for training was recognised;

> *I think there is a huge lack in primary care education, and I think that goes to show by the starting people coming in are only on like a 40% of them are on Best Medical Therapy so I think a big thing to target would be primary care to inform them* (QR18, secondary care clinician, UHL).

GPs went on to request that all primary care staff be involved and not just GPs, and that the primary care training be delivered by the vascular team.

Theme 4: Personalised information and supportive conversations for taking on the advice

For peer review only - http://bmjopen.bmj.com/site/about/guidelines.xhtml

Providers and patients spoke about the delivery of the intervention, recognising the importance of offering personalised care and tailoring patient information to individual needs. Secondary care clinicians felt that the intervention should be adapted to identify realistic targets and individual goals, *or the advice rings hollow* (QR13), and to offer a different approach for each patient. Staff reported;

> *I think the hard thing as well is that with the LEGS bundle, sometimes you're giving it to someone who's just had an amputation or about to have an amputation so it's just to be mindful you're talking about things like exercise they look at you very disparagingly and that can be a bit tricky… I think it's a really good checklist and bundle for claudicants and I think that's where it really shines but I think for when you get to the stage of CLTI, I think those individuals often feel that they're too far gone for you to talk to them about lifestyle modification and things like that* (QR18, secondary care clinician, UHL).

Patients also spoke about the importance of timing in terms of when they received the lifestyle information.

> *If I was a bit younger and I'd got more get-up-and-go, I would gladly have a leaflet and go through it and take up advice and what have you. But as I am at the moment, my health is far from good* (QR14, in-patient care, UHL).

> *…it is a bit too late in that respect so I can only do what I can do. I mean yesterday I was out in the garden doing some weeding but I have to use a seat to do it* (QR05, in-patient care, UHL).

Patients explained that they like to receive information face-to-face and suggested that the education leaflet could be used as a supportive conversation guide. They emphasised the importance of the conversation that goes alongside the advice and information, and the relationship that they have with their health professional(s) in terms of asking questions and being able to talk about making changes like smoking cessation. One patient explained,

> *… the only thing I've got to do is drink and smoke and watch the tv – cause if I didn't have those three things, I would be pulling my hair out… to be quite honest, I'm quite happy at the moment to be carrying on the way I am. It might sound silly to you, but I enjoy smoking. I love a drink, I like my telly… if you're brow-beaten into packing up when you don't really want to, all this, it can have a great effect on people* (QR14, in-patient care, UHL).

GPs and secondary care clinicians acknowledged the need to support patient preference, to involve patients in the conversation and to share decision making.

For peer review only - http://bmjopen.bmj.com/site/about/guidelines.xhtml

…you have to strike a balance, and therefore taking the patient's preferences into account is important. We assume that patients want to live the longest life they can, I'm not sure that's always correct. Some patients would rather accept that they live a slightly shorter life but with the benefit of enjoying certain things that we all know are less healthy (QR13, secondary care clinician, UHL).

It was acknowledged that everyone knows they need to stop smoking and it is easy to bombard people with information. Solutions such as motivational interviewing techniques were identified by one GP as a way to support engagement conversations. Secondary care clinicians felt that providing information on the benefits of following the advice versus taking pleasure away might help. For others, frank conversations that explain the risks help;

I think honesty is the most important aspect of this and bringing people in front of the data, highlighting the data, and actually not sugar coating anything. We know, for example, that if you have critical ischemia, so severe peripheral arterial disease, if your prognosis is worse, your five-year survival is worse than some kinds of cancer. So, if you tell someone that unless you actually take this intervention seriously, your likelihood of not being around in five years is very high. I know it sounds bleak, but it may be the wake-up call that someone needs. Or explaining that from the moment you have severe arterial peripheral disease, the likelihood of you losing your leg and being wheelchair bound may be an important thing to highlight as opposed to speaking in a circumspect way. I think just being honest and explaining the risks may be a way of engaging patients. (QR15, secondary care clinician, UHL).

A patient explained how the honest frank information helped;

The doctor actually threatened me. He said to me, if I'm going to do this operation, you got to stop smoking. So, I did. That helped, or I could lose my leg. I thought it was brilliant the way [the doctor] come out with it and I've never stopped thanking him for it. It works (QR06, out-patient care, GSTT).

Discussion

The aim of the study was to provide an understanding of the feasibility and implementation ~~acceptability~~ of the LEGS (LEaflet Gp letter Structured checklist) intervention, a complex intervention in the form of a healthcare bundle, designed to improve the care of patients with PAD in the NHS. Our inductive approach to analysis found four overarching themes: i) *the potential to make a difference, ii) a solution to address the gap in no man's land, iii) prioritising and making it happen* and *iv) personalised information and supportive conversations for taking on the advice.* We used the Consolidated Framework of Implementation Research (CFIR) to

For peer review only - http://bmjopen.bmj.com/site/about/guidelines.xhtml

guide our analysis, and all five CFIR domains are represented (13). The CFIR is well-placed for developing a model for implementing secondary care-initiated treatment in primary care (17).

All participants spoke positively about using the intervention to make a difference to patient care~~, and~~ as an opportunity to meet patient needs, and as a way to ~~. Moreover, a way to~~ enhanc~~e~~ the quality of PAD care through improved working practices. This promotes a streamlined patient pathway from primary care to secondary care and to shared care. This reflects the value of an agreed pathway of care and shared partnership working that provides an opportunity for the intervention to be delivered as part of a wider team and to prioritise patient-centred care (17).

There was a consensus of optimism in participant accounts about the potential to make a difference. While barriers to seeking care for those with PAD are well-documented (18)(Tan et al., 2022. The findings confirm that the period between diagnosis of early-stage PAD (e.g., claudication) and subsequent potential diagnosis of advanced PAD with severe symptoms, is a time of no man's land, when patients do not receive any help, education, support, or appropriate medication to address their condition (9). As such, the benefits of the LEGS intervention were identified in terms of meeting the needs of this subgroup of patients, and to address the gap in care. GPs were optimistic that they can deliver the LEGS intervention with training and ongoing sustained communication with secondary care. The findings highlight the need to consider training requirements and systems for maintaining open primary-secondary care communication to support implementation. For example, education on tailoring the intervention for patients at both early and late stages of PAD, that can be used across primary and secondary care settings, similar to the model used with other chronic conditions (17, 19) Improving the awareness of PAD among healthcare providers and supporting patients to take responsibility for their own health are therefore important determinants in the success of implementation (20).

For peer review only - http://bmjopen.bmj.com/site/about/guidelines.xhtml

While the three elements of the LEGS intervention have been designed to facilitate interaction between secondary and primary care following diagnosis of PAD, the intervention focus until now, has been on secondary care services, that is, hospital admission or out-patient clinic. However, our findings indicate that the intervention can be used to address the gap in service provision for patients who do not yet meet the referral criteria for secondary care services. We therefore propose to extend the use of the LEGS intervention across the primary-secondary care interface. Our findings also confirm the need for individualised patient care, supportive communication and shared decision making in order to take on the advice in a more effective way (21). The finalised LEGS intervention should therefore promote supportive conversation between patient and healthcare staff, both in primary and secondary care settings. The open approach to changing lifestyle and behaviour could be built on techniques like motivational interviewing, which is already commonly used in primary care practice (22). Lastly, our findings confirm that healthcare providers are optimistic and positive about the introduction of an intervention which will help them to deliver more consistent and guideline-based PAD treatment. The challenge now is to take this enthusiasm together with the lessons learned from this qualitative research to adapt the LEGS intervention before wider testing and adoption in the NHS. The main changes will relate to usability in primary care after diagnosis of early-stage PAD, facilitation of effective communication not only between primary and secondary care staff, but also patients and staff, as well as focussing on specific needs of patients by prioritising areas to focus on in an incremental or stepwise manner.

~~Conclusions~~

~~The LEGS intervention can be tailored for use at early and late stages of PAD, can be provided across primary and secondary care settings, and provides an opportunity to promote shared working across the primary-secondary care interface. These findings are important to the specifics of the CHABLIS study but can also go some way in informing the wider learning for other interventions that are implemented across the primary-secondary care interface.~~

For peer review only - http://bmjopen.bmj.com/site/about/guidelines.xhtml

Strengths and limitations

The paper adds new knowledge on the development and implementation of a community and hospital intervention for PAD.~~eripheral Arterial Disease.~~ It is important to note that the findings are specific to the CHABLIS study. It is possible, however, to enhance transferability by describing the research context and assumptions, and by making connections between the analysis of participants accounts and claims in the extant literature.

While the sample size for this study may seem small, the volume of interview data we collected is unique for this patient population. Many factors influence the success of recruiting participants in research and engaging vulnerable patient populations brings additional complexity (23). PAD is a chronic and progressive disease with burdensome symptoms including physical disability, cardiovascular risks and mortality (24). Those who live with PAD are vulnerable because they are living with extreme frailty, often with the threat or result of limb loss, and with risks associated with poorer socio-economic conditions (25). Alongside, patients with PAD might be less receptive to engaging with research because they experience specific barriers to accessing healthcare meaning that taking part in research alongside managing health care and health needs might be considered onerous (26).

A strength is the focus on rigor and triangulation of participant perspectives. We also acknowledged the complexity of intervention implementation across the secondary-primary care interface and therefore used a systems research perspective for addressing implementation at the outset of the research. We used implementation theory to guide analysis and to provide a layered coding approach, to ensure that intervention and implementation recommendations are novel, grounded in the data, and useful (13).

While the anonymity of telephone use can allow participants to disclose sensitive information, telephone interviews have received criticism for compromising interviewer/participant rapport and interaction, and for limiting contextual data due to the absence of face-to-face contact and

For peer review only

visual cues. However, this method of data collection is convenient, in that it is flexible (in terms of time and location), accessible (i.e., remote research conducted during the COVID-19 pandemic), and allows for a wide reach (e.g., accessing a diverse population from both urban and rural areas) (27).

Conclusions

The LEGS intervention can be tailored for use at early and late stages of PAD, can be provided across primary and secondary care settings, and provides an opportunity to promote shared working across the primary-secondary care interface. These findings are important to the specifics of the CHABLIS study but can also go some way in informing the wider learning for other interventions that are implemented across the primary-secondary care interface.

Funding

This article presents independent research funded by National Institute for Health Research for Patient Benefit (RfPB) Programme (funder's reference: NIHR202008). The views expressed are those of the author(s) and not necessarily those of the NIHR or the Department of Health and Social Care.

Competing interests

The authors declare that they have no competing interests.

Authors' contributions

CL contributed to the qualitative study design, coordinated the study, led data collection and analysis, and drafted the manuscript. AS is principal investigator to the CHABLIS study, acquired funding, participated in the study design, contributed to data collection, and drafted the manuscript. PS contributed to data collection and reviewed the manuscript. RB and BB drafted the manuscript. EW contributed to data collection and reviewed the manuscript. VL

For peer review only

designed the qualitative study, provided oversight on study coordination, data collection and analysis, and drafted the manuscript. All authors read and approved the final manuscript.

Consent for publication

Consent for publication has been obtained from all research participants.

Word count

4,775

Availability of data and materials

This study uses data (containing potentially identifying and/or sensitive information) collected from a small group of staff participants and a vulnerable patient population, and involves indirect identifiers (such as sex, ethnicity, location, etc.) that may risk the identification of study participants. Sharing data outside of the anonymised excerpts and quotations included in the paper will violate the agreement to which the participants consented.

Acknowledgements

We would like to thank all patients, secondary care staff and GPs who participated in this study and who generously gave their time and honest thoughts. We would like to thank the local research teams for their support in recruiting to this qualitative study and we are also grateful to the NIHR Clinical Research Network Research Delivery Managers that helped to recruit GP participants.

Ethics Statement

The research was reviewed and approved by the NHS Wales Research Ethics Committee (REC), reference number20/WA/0319 and the NHS Health Research Authority (HRA).

References

For peer review only - http://bmjopen.bmj.com/site/about/guidelines.xhtml

1. Herrington W, Lacey B, Sherliker P, Armitage J, Lewington S. Epidemiology of Atherosclerosis and the Potential to Reduce the Global Burden of Atherothrombotic Disease. Circ Res. 2016;118(4):535-46.

2. Cea-Soriano L, Fowkes FGR, Johansson S, Allum AM, García Rodriguez LA. Time trends in peripheral artery disease incidence, prevalence and secondary preventive therapy: a cohort study in The Health Improvement Network in the UK. BMJ Open. 2018;8(1):e018184.

3. Dhaliwal G, Mukherjee D. Peripheral arterial disease: Epidemiology, natural history, diagnosis and treatment. Int J Angiol. 2007;16(2):36-44.

4. Parvar SL, Fitridge R, Dawson J, Nicholls SJ. Medical and lifestyle management of peripheral arterial disease. J Vasc Surg. 2018;68(5):1595-606.

5. Nehler MR, Duval S, Diao L, Annex BH, Hiatt WR, Rogers K, et al. Epidemiology of peripheral arterial disease and critical limb ischemia in an insured national population. J Vasc Surg. 2014;60(3):686-95.e2.

6. Dua A, Lee CJ. Epidemiology of Peripheral Arterial Disease and Critical Limb Ischemia. Tech Vasc Interv Radiol. 2016;19(2):91-5.

7. Morley RL, Sharma A, Horsch AD, Hinchliffe RJ. Peripheral artery disease. Bmj. 2018;360:j5842.

8. National Institute for Health and Care Excellence. Peripheral arterial disease: diagnosis and management. 2020.

9. Saratzis A, Jaspers NEM, Gwilym B, Thomas O, Tsui A, Lefroy R, et al. Observational study of the medical management of patients with peripheral artery disease. Br J Surg. 2019;106(9):1168-77.

10. Skivington K, Matthews L, Simpson SA, Craig P, Baird J, Blazeby JM, et al. A new framework for developing and evaluating complex interventions: update of Medical Research Council guidance. Bmj. 2021;374:n2061.

11. Tong A, Sainsbury P, Craig J. Consolidated criteria for reporting qualitative research (COREQ): a 32-item checklist for interviews and focus groups. Int J Qual Health Care. 2007;19(6):349-57.

12. Braun V, Clarke V. Using thematic analysis in psychology. Qualitative Research in Psychology. 2006;3(2):77-101.

13. Damschroder LJ, Aron DC, Keith RE, Kirsh SR, Alexander JA, Lowery JC. Fostering implementation of health services research findings into practice: a consolidated framework for advancing implementation science. Implement Sci. 2009;4:50.

14. Braun V, Clarke V. One size fits all? What counts as quality practice in (reflexive) thematic analysis? Qualitative Research in Psychology. 2021;18(3):328-52.

15. QSR International Pty Ltd. NVivo. 2018.

16. Bammer G. Key issues in co-creation with stakeholders when research problems are complex. Evidence & Policy. 2019;15(3):423-35.

17. Le Boutillier C, Ahmed H, Patel V, McPhail M, Carter B, Ward C, et al. General Practitioner perspectives on factors that influence implementation of secondary care-initiated treatment in primary care: exploring implementation beyond the context of a clinical trial. PLoS One. 2022.

18. Meffen A, Houghton JSM, Nickinson ATO, Pepper CJ, Sayers RD, Gray LJ. Understanding variations in reported epidemiology of major lower extremity amputation in the UK: a systematic review. BMJ Open. 2021;11(10):e053599.

For peer review only - http://bmjopen.bmj.com/site/about/guidelines.xhtml

19. Smith SM, Cousins G, Clyne B, Allwright S, O'Dowd T. Shared care across the interface between primary and specialty care in management of long term conditions. Cochrane Database Syst Rev. 2017;2(2):Cd004910.

20. Bridgwood BM, Nickinson AT, Houghton JS, Pepper CJ, Sayers RD. Knowledge of peripheral artery disease: What do the public, healthcare practitioners, and trainees know? Vasc Med. 2020;25(3):263-73.

21. Barry MJ, Edgman-Levitan S. Shared decision making--pinnacle of patient-centered care. N Engl J Med. 2012;366(9):780-1.

22. Rubak S, Sandbaek A, Lauritzen T, Christensen B. Motivational interviewing: a systematic review and meta-analysis. Br J Gen Pract. 2005;55(513):305-12.

23. Le Boutillier C, Snowdon C, Patel V, McPhail M, Ward C, Carter B, et al. Using a theory-informed approach to explore patient and staff perspectives on factors that influence clinical trial recruitment for patients with cirrhosis and small oesophageal varices. PLoS One. 2022;17(2 February).

24. Adam DJ, Beard JD, Cleveland T, Bell J, Bradbury AW, Forbes JF, et al. Bypass versus angioplasty in severe ischaemia of the leg (BASIL): multicentre, randomised controlled trial. Lancet. 2005;366(9501):1925-34.

25. Brostow DP, Hirsch AT, Kurzer MS. Recruiting older patients with peripheral arterial disease: evaluating challenges and strategies. Patient Prefer Adherence. 2015;9:1121-8.

26. Tan TW, Crocker RM, Palmer KNB, Gomez C, Armstrong DG, Marrero DG. A qualitative study of barriers to care-seeking for diabetic foot ulceration across multiple levels of the healthcare system. J Foot Ankle Res. 2022;15(1):56.

27. Irvine A. Duration, Dominance and Depth in Telephone and Face-to-Face Interviews: A Comparative Exploration. International Journal of Qualitative Methods. 2011;September:202-20.

For peer review only

For peer review only - http://bmjopen.bmj.com/site/about/guidelines.xhtml

BMJ Open

Factors that influence the feasibility and implementation of a complex intervention to improve the treatment of Peripheral Arterial Disease in primary and secondary care: a qualitative exploration of patient and provider perspectives

Journal:	*BMJ Open*
Manuscript ID	bmjopen-2022-066883.R2
Article Type:	Original research
Date Submitted by the Author:	24-Nov-2022
Complete List of Authors:	Le Boutillier, Clair; King's College London<br>Saratzis, Athanasios; University of Leicester<br>Saha, Prakash; Guy's and St Thomas' NHS Foundation Trust<br>Benson, Ruth; University of Birmingham<br>Bridgwood, Bernadeta; University of Leicester<br>Watson, Emma; University of Leicester<br>Lawrence, Vanessa; King's College London, Health Services & Population Research Department
**Primary Subject Heading**:	Cardiovascular medicine
Secondary Subject Heading:	Qualitative research, Patient-centred medicine, Cardiovascular medicine
Keywords:	QUALITATIVE RESEARCH, VASCULAR MEDICINE, Change management < HEALTH SERVICES ADMINISTRATION & MANAGEMENT

SCHOLARONE™
Manuscripts

I, the Submitting Author has the right to grant and does grant on behalf of all authors of the Work (as defined in the below author licence), an exclusive licence and/or a non-exclusive licence for contributions from authors who are: i) UK Crown employees; ii) where BMJ has agreed a CC-BY licence shall apply, and/or iii) in accordance with the terms applicable for US Federal Government officers or employees acting as part of their official duties; on a worldwide, perpetual, irrevocable, royalty-free basis to BMJ Publishing Group Ltd ("BMJ") its licensees and where the relevant Journal is co-owned by BMJ to the co-owners of the Journal, to publish the Work in this journal and any other BMJ products and to exploit all rights, as set out in our licence.

The Submitting Author accepts and understands that any supply made under these terms is made by BMJ to the Submitting Author unless you are acting as an employee on behalf of your employer or a postgraduate student of an affiliated institution which is paying any applicable article publishing charge ("APC") for Open Access articles. Where the Submitting Author wishes to make the Work available on an Open Access basis (and intends to pay the relevant APC), the terms of reuse of such Open Access shall be governed by a Creative Commons licence – details of these licences and which Creative Commons licence will apply to this Work are set out in our licence referred to above.

Other than as permitted in any relevant BMJ Author's Self Archiving Policies, I confirm this Work has not been accepted for publication elsewhere, is not being considered for publication elsewhere and does not duplicate material already published. I confirm all authors consent to publication of this Work and authorise the granting of this licence.

For peer review only

Title: **Factors that influence the feasibility and implementation of a complex intervention to improve the treatment of Peripheral Arterial Disease in primary and secondary care: a qualitative exploration of patient and provider perspectives**

Authors: Clair Le Boutillier[1,2], Athanasios Saratzis[3], Prakash Saha[4], Ruth Benson[5], Bernadeta Bridgwood[6], Emma Watson[3,6], Vanessa Lawrence*[1]

[1] Institute of Psychiatry, Psychology & Neuroscience, King's College London, UK
[2] Florence Nightingale Faculty of Nursing, Midwifery & Palliative Care, King's College London, UK
[3] University of Leicester, UK
[4]Guy's and St Thomas' NHS Foundation Trust, UK
[5]University of Birmingham, UK
[6]NIHR Cardiovascular Sciences, University of Leicester, Leicester, UK.

* Corresponding authors. Email: vanessa.c.lawrence@kcl.ac.uk; clair.le_boutillier@kcl.ac.uk

Division of Methodologies, Florence Nightingale Faculty of Nursing, Midwifery & Palliative Care, King's College London, James Clerk Maxwell Building, 57 Waterloo Road, London SE1 8WA. Orcid iD: 0000-0003-3665-0315

Funding

The study was funded by the National Institute for Health Research (NIHR) – Research for Patient Benefit (RfPB) Programme (Reference: NIHR202008).

Category

Original article

For peer review only - http://bmjopen.bmj.com/site/about/guidelines.xhtml

Abstract

Objectives: Our aim was to examine the feasibility and implementation of a complex intervention to improve the care of patients with Peripheral Arterial Disease (the LEGS intervention) from the perspective of patients, general practitioners and secondary care clinicians.

Design: A qualitative study involving semi-structured individual interviews with patients and providers to gain an understanding of the feasibility of the LEGS intervention as well the barriers and facilitators to implementation in secondary and primary care.

Setting: Primary and secondary care settings across two NHS Trusts.

Participants: Twenty-five semi-structured telephone interviews were conducted with i) patients who had received the intervention (n=11), ii) secondary care clinicians responsible for delivering the intervention (n=8), and iii) general practitioners (n=6).

Analysis Data were initially analysed using inductive descriptive thematic analysis. The consolidated framework for implementation research was then used as a matrix to explore patterns in the data and to map connections between the three participant groups. Lastly, interpretive analysis allowed for refining, and a final coding frame was developed.

Results: Four overarching themes were identified: i) The potential to make a difference, ii) A solution to address the gap in no man's land, iii), Prioritising and making it happen and iv) Personalised information and supportive conversations for taking on the advice. The impetus for prioritising and delivering the intervention was further driven by its flexibility and adaptability to be tailored to the individual and to the environment.

Conclusions: The LEGS intervention can be tailored for use at early and late stages of Peripheral Arterial Disease, provides an opportunity to meet patient needs and can be used to promote shared working across the primary-secondary care interface.

For peer review only - http://bmjopen.bmj.com/site/about/guidelines.xhtml

Strengths and limitations

- A strength of this study is the focus on rigor and triangulation of participant perspectives.
- We used implementation theory to guide analysis and to provide a layered coding approach, to ensure that intervention and implementation recommendations are novel, grounded in the data, and useful.
- While the anonymity of telephone use can allow participants to disclose sensitive information, telephone interviews have received criticism for compromising interviewer/participant rapport and interaction, and for limiting contextual data due to the absence of face-to-face contact and visual cues. However, this method of data collection is convenient, in that it is flexible (in terms of time and location), accessible (i.e., remote research conducted during the COVID-19 pandemic), and allows for a wide reach (e.g., accessing a diverse population from both urban and rural areas).
- Although the sample size for this study may seem small, the volume of interview data we collected is unique for this patient population. Living with the symptoms of Peripheral Arterial Disease is challenging, and patients also experience barriers to accessing healthcare. Engaging in research alongside managing these specific health care and health needs can be burdensome.

List of abbreviations

PAD	Peripheral Arterial Disease
IC	Intermittent Claudication
CLTI	Chronic Limb Threatening Ischaemia
GP	General Practitioner
CHABLIS	Community and Hospital cAre Bundle to improve the medical treatment of cLaudIcation and critical limb iSchaemia
LEGS	LEaflet Gp letter Structured checklist
UHL	University Hospitals of Leicester NHS Trust
GSTT	Guy's and St Thomas' NHS Foundation Trust
CFIR	The consolidated framework for implementation research
NHS	National Health Service

For peer review only

Background

Peripheral Arterial Disease (PAD) affects a fifth of people over the age of sixty in the United Kingdom. This prevalence is also expected to rise due to sedentary lifestyles, poor dietary choices, a longer life-expectancy, and rising numbers of those with diabetes (1, 2). Patients with PAD who develop symptoms present either with Intermittent Claudication (IC) or in some cases, Chronic Limb Threatening Ischaemia (CLTI) where the reduction in blood flow is so severe that it causes pain at rest, ulceration, or gangrene (3). Patients with IC are more likely to suffer a cardiovascular event compared to age-matched individuals, and the one-year risk of limb amputation for those with CLTI is 30%. More than half of those diagnosed with symptomatic PAD are expected to die, have an amputation or a major cardiovascular event within five years (4-7).

The treatment of these high-risk individuals is shared between primary and secondary care. While established national and international guidance (8) outlines how PAD risk-factors should be addressed to prevent cardiac events and amputations, a study conducted by our group found that only a tenth of those diagnosed with PAD were prescribed appropriate preventive medication, and almost none received structured lifestyle or dietary support (9). As a result, and following patient and public engagement (that is, interviews and workshops with patients and a national consultation with primary and secondary care clinicians) and a review of the existing evidence, a complex intervention in the form of a care bundle was developed to support patients, General Practitioners (GPs), and secondary care clinicians to actively manage cardiovascular risk-factors, and to improve implementation of existing guidance when treating patients with PAD. The care bundle, called the LEGS intervention (Leaflet, Gp letter, Structured checklist) has three elements: i) a one-page clinical checklist for the ward and a separate one-page clinical checklist for the secondary care clinic, ii) a patient and carer education leaflet (developed with patients), and iii) a GP action letter, that can be sent to GPs at regular intervals to inform them of the PAD diagnosis and corresponding practice guidance.

For peer review only - http://bmjopen.bmj.com/site/about/guidelines.xhtml

The LEGS intervention and full study protocol are available online (isrctn.com/ISRCTN13202085).

This qualitative study was embedded within the Community and Hospital cAre Bundle to improve the medical treatment of cLaudIcation and critical limb iSchaemia (CHABLIS) study. CHABLIS is a multicentre prospective mixed-methods cohort study that aims to determine the feasibility and effectiveness of the LEGS intervention. The aim of the qualitative study was to gain an understanding of the feasibility and implementation of the LEGS intervention, by exploring patient and provider perspectives on receiving and delivering the intervention (10). Guided by the MRC framework for developing and evaluating complex interventions, the objectives of the qualitative study were: 1) to explore patient perspectives on receiving the LEGS intervention, 2) to gather secondary care clinician views on feasibility, and to understand factors that influence the success of implementation, and 3) to identify barriers and facilitators that influence the success of future implementation from the GP perspective.

METHODS

Study design

The study used qualitative methods to provide insights into the feasibility and acceptability of the intervention as well as the complexities of implementation (10). Semi-structured individual interviews were conducted with patients and providers to gain an understanding of the barriers and facilitators to implementation in secondary and primary care. The research was reviewed and approved by the NHS Wales Research Ethics Committee (REC), reference number20/WA/0319 and the NHS Health Research Authority (HRA). The consolidated criteria for reporting qualitative research (COREQ) guideline was used to ensure quality when reporting the research (11).

To meet objective 1, semi-structured individual interviews were conducted with patients with symptomatic PAD (i.e., IC, ischaemic rest pain, or CLTI), who had received the intervention

For peer review only - http://bmjopen.bmj.com/site/about/guidelines.xhtml

(either in an outpatient clinic or as a part of inpatient treatment). To meet objective 2, semi-structured individual interviews were conducted with secondary care clinicians who were responsible for delivering (that is, delivering or intending to deliver) the intervention, including junior doctors, vascular nurses, and vascular surgeons. Staff and patients were recruited from two implementation sites: University Hospitals of Leicester NHS Trust (UHL) and Guy's and St Thomas' NHS Foundation Trust (GSTT). To meet objective 3, semi-structured individual interviews were conducted with GPs to gain an understanding of the barriers and facilitators to future implementation in primary care and across the NHS. GPs were not involved in delivering the study intervention and were asked to report on perceived factors that might influence implementation beyond the context of the study.

Participants were selected purposively (both those who were receiving or responsible for delivering the LEGS intervention) on the basis that they are the target adopters of behaviours that influence the success of future implementation and/or could offer a particular perspective on implementation. In total, 106 patients received the intervention. All patients who had received the intervention and who gave consent for the researcher to make contact about the qualitative study were approached for participation (n=26). All secondary care clinicians responsible for delivering the intervention (n=13) were invited to take part. Secondary care clinicians were invited to participate following an in-person meeting with the local principal investigator at each site. A participant information sheet that outlined the study was provided to all potential participants with time to consider whether or not they would like to take part. GP participants were recruited from Clinical Commissioning Groups linked to the two secondary care implementation sites, chosen to enhance joined up working and to provide a local perspective on implementation across community and hospital settings. Potential GP participants (n=7) were first approached by the local Clinical Research Network research delivery manager or local contacts, and subsequently recruited by the lead author via telephone or email. Written or verbal informed consent was obtained from each participant after a full explanation and information leaflet was given and time allowed for consideration.

For peer review only - http://bmjopen.bmj.com/site/about/guidelines.xhtml

Participants were not reimbursed for participating in the research. The right of each participant to refuse to participate without giving reasons was respected. All participants were free to withdraw from the study at any time without giving reasons and without prejudicing further treatment or employment.

Data collection

Interviews used flexible open-ended questions for early data collection to gather a rich and detailed understanding of participants' perspectives (included in Online Data Supplement 1). The patient and provider interview schedules were revised iteratively in response to the priorities and concerns of participants and gathered information on factors that facilitate or hinder implementation and on possible solutions to overcome any identified barriers. Interviews were conducted by telephone by the lead author (a qualitative research fellow), lasted around 45 minutes, were audio-recorded and transcribed verbatim. Researcher reflexive notes were kept after each interview to consider the interaction with the participant, and to detail initial thoughts. Interviews were conducted between September and November 2021.

Data Analysis

Data from the three participant groups (patients, secondary care clinicians and GPs) were first analysed separately using inductive thematic analysis, where analytical concepts and perspectives are generated from the data in a deliberate and systematic way (12). Data analysis began with repeated re-reading of individual transcripts and re-listening of sound files for data immersion. This was followed by line-by-line open coding, where extracts were coded under one or several descriptive themes, that is organising the data according to semantic content, to capture their meaning and reflect the content of the data. Each theme was refined, and where data allowed, further sub-themes were developed. The Consolidated Framework for Implementation Research (CFIR) was then used as a matrix to organise the early themes, to explore patterns and relationships in the data, and to map connections between the themes

For peer review only

For peer review only - http://bmjopen.bmj.com/site/about/guidelines.xhtml

and the three participant groups (13). Lastly, interpretive analysis allowed for the refining of the specifics of themes and thematic patterns, and a final coding frame for patients and providers was developed. Refinements to the specifics of themes, and thematic patterns continued until a useful and meaningful analysis was achieved (14).

Data collection occurred concurrently with data analysis; NVivo QSR International qualitative analysis software (version 12) was used to manage the data. (15). The lead author (CL) directed and conducted the analysis. Coding by a second analyst (VL) was undertaken to provide an opportunity to reflect on the coding approach, and to enhance the interpretive depth of the data (14). This involved an independent review of the data, the themes, and the CFIR mapping/tabulation. The overarching coding framework was developed following discussion with both analysts.

Patient and public involvement

The LEGS intervention and CHABLIS mixed-methods study design were developed in collaboration with a stakeholder PPI group that included patients, family members and carers, and clinicians working in primary and secondary care. (16).

RESULTS

Participants

A total of 25 individual interviews were conducted with i) patients who had received the intervention (n=11), ii) secondary care clinicians responsible for delivering the intervention (n=8), and iii) GPs (n=6). Two of the eight secondary care clinicians were actively delivering the intervention (in an in-patient setting), and all GPs provided insights on future implementation. Six patients had received the intervention as a part of in-patient care and five patients provided perspectives on receiving the intervention from out-patient vascular, ulcer, or specialised CLTI clinics. Patient participants spoke about living with multiple conditions including diabetes, stroke, and amputation and receiving care from primary care (GPs, district

For peer review only - http://bmjopen.bmj.com/site/about/guidelines.xhtml

nurses, diabetic nurses) secondary care (vascular teams), social care (carers), and family. Three patients were >70 years old but all patients were retired or unemployed and spoke about the impact of PAD on quality of life including managing pain, slow wound healing, poor sleep, reduced daily functioning, and limited mobility. Patient characteristics are shown in Table 1 and provider characteristics are detailed Table 2.

Table 1: Patient participant characteristics

**Patient characteristics** n (%)	Patients n=11
**Age**	
41-50 years	1 (9.1)
51-60 years	0 (0.00)
61-70 years	2 (18.2)
>70 years	8 (72.7)
**Gender**	
Male	8 (72.7)
Female	3 (27.3)
**Ethnicity**	
White British	9 (81.8)
White Irish	1 (9.1)
Black/Black British-Caribbean	1 (9.1)
**Marital status**	
Single	2 (18.2)
Married	7 (63.6)
Widowed	2 (18.2)
**Time since diagnosis**	
>1 year	3 (27.3)
1-4 years	4 (36.35)
5-9 years	0 (0.00)
10 years +	4 (36.35)
**No. of surgical interventions**	
None	1
Angioplasty	8
Bypass graft	4
Amputation	2

For peer review only

Table 2: Provider participant characteristics

**Provider characteristics** n (%)	Hospital staff n=8	GPs n=6
**Age**		
21-30 years	3 (37.5)	1 (16.7)
31-40 years	5 (62.5)	3 (50.0)

41-50 years	0 (00.0)	2 (33.3)
**Gender**		
Male	4 (50.0)	4 (66.7)
Female	4 (50.0)	2 (33.3)
**Ethnicity**		
White British	2(25.0)	3 (50.0)
White Other	1 (12.5)	1 (16.7)
Asian/Asian British-Indian	2 (25.0)	2 (33.3)
Mixed. White & Asian	1 (12.5)	0 (00.0)
Chinese	1 (12.5)	0 (00.0)
Other	1 (12.5)	0 (00.0)
**Core Profession**		
Nurse	1 (12.5)	0 (00.0)
Vascular surgeon	7 (87.5)	0 (00.0)
GP trainee	0 (00.0)	1 (16.7)
General practitioner	0 (00.0)	5 (83.3)
**Grade**		
Band 8a	1 (12.5)	0 (00.0)
Senior House Officer	2 (25.0)	0 (00.0)
Specialty trainee	1 (12.5)	1 (16.7)
Speciality registrar	2 (25.0)	0 (00.0)
Consultant	2 (25.0)	0 (00.0)
GP	0 (00.0)	5 (83.3)
**Time since qualification**		
0-2 years	0 (00.0)	1 (16.7)
2 years + -5 years	0 (00.0)	1 (16.7)
5 years + -10 years	3 (37.5)	2 (33.3)
10 years + -15 years	5 (62.5)	2 (33.3)
**Time in current post**		
>6 months	5 (62.5)	1 (16.7)
7-12 months	0 (00.0)	0 (00.0)
13-24 months	2 (25.0)	2 (33.3)
25-36 months	0 (00.0)	1 (16.7)
37-48 months	1 (12.5)	0 (00.0)
49-60 months	0 (00.0)	0 (00.0)
61-75 months	0 (00.0)	1 (16.7)
13 years	0 (00.0)	1 (16.7)

Of the patient participants contacted by the researcher, (11/26) 42% agreed to take part in an interview (fifteen people declined) and (8/13) 82% of staff agreed to take part (1 person declined participation and 4 people did not respond to the invitation). Reasons for non-participation for patients included being unable to remember the intervention (n=5), deteriorating health (n=7), just home from an in-patient admission (n=1), being admitted for angioplasty (n=1) and getting back on with life (n=1). The reason provided by providers for declining participation was moving to a new role and lack of time.

For peer review only

For peer review only - http://bmjopen.bmj.com/site/about/guidelines.xhtml

Secondary care clinicians and GPs spoke about all three elements of the intervention and patients provided insights on the education leaflet and GP action letter. The descriptive themes were mapped to implementation theory using the CFIR, to explore connections between the themes, and to compare findings across participant groups.

Using the CFIR to facilitate analysis

The CFIR comprises five domains that provide an overarching typology of implementation theory (in terms of what works where and why): i) intervention characteristics (features of the intervention such as complexity), ii) outer setting (the external context that might influence implementation such as health policy and the extent to which patient needs, as well as barriers and facilitators to meet those needs are known and prioritised), iii) inner setting (features of the implementing organisation such as leadership, implementation readiness), iv) characteristics of individuals involved in implementation (such as beliefs and knowledge about the intervention), iv) implementation process (13). The themes map to all five overarching CFIR domains across participant groups: intervention characteristics, outer setting, inner setting, characteristics of individuals and process. In summary, for intervention characteristics, all participant groups spoke about the advantage of implementing the care bundle, provided insights on improvements to the design quality and packaging, and acknowledged the need to adapt and tailor the intervention to meet the needs of individuals. Complexity was identified in terms of implementing the intervention across the primary-secondary care interface.

For outer setting, the specific needs of the patient group and barriers and facilitators to meet those needs were highlighted, with all participant groups explaining that a delayed referral to secondary care is a barrier to implementing the intervention. Staff participants went on to speak about external policies and incentives that might influence the spread and uptake of the intervention. For inner setting, the structural characteristics and the quality of networks and communications within and across each organisation were identified by all groups as influences on implementation. For structural characteristics, participants explained that the

For peer review only - http://bmjopen.bmj.com/site/about/guidelines.xhtml

ability to follow the advice in the education leaflet is often determined by the availability of supervised exercise programmes, difficulty ordering medication, and relying on carers for food shopping. For networks and communications, participants spoke about the need to work as a three-way team, and to have ongoing access to the specialist vascular team. For characteristics of individuals, staff values, attitude, knowledge, and skills to provide the intervention and patient readiness to take on the advice of the intervention were highlighted across participant groups. The patient-healthcare provider relationship was identified as a central influence on implementation and there was a particular emphasis on individual stage of change, with staff participants highlighting a need to engage patients in their care and to promote shared decision making. The mapping of themes across CFIR domains and participant groups is included in Online Data Supplement 2.

A final coding frame for patients, secondary care clinicians and GPs was developed following interpretive analysis. Because of space limitations, a summary of each theme is provided with subthemes being used to define each overarching theme; the full coding framework is included in Online Data Supplement 3. Four overarching themes were identified: *the potential to make a difference, a solution to address the gap in no man's land, prioritising and making it happen* and *personalised information and supportive conversations for taking on the advice.* Similarities and differences between participant groups are presented where they arise.

Theme 1: The potential to make a difference

GPs and secondary care clinicians felt that the LEGS intervention provides an opportunity to meet patient need and to support implementation of NICE guidance and spoke optimistically about using the intervention to improve patient outcomes and to standardise a minimum quality of care.

> *…it standardises the quality of care and standardisation is a big problem that we have in vascular surgery because guidance is so vague. So, I think it raises awareness and standardises that at least we ensure the patient gets a minimum of care* (QR15, secondary care clinician, UHL).

For peer review only - http://bmjopen.bmj.com/site/about/guidelines.xhtml

GPs and secondary care clinicians went on to explain how the intervention can be used to inform, empower, and support relationship building with patients.

> *I've gone through the leaflet with them… and knowing that the patient has understood I will spend a couple of minutes going through the leaflet to make sure. If they've got any questions I will try and take the opportunity to build my relationship with the patient so that they do understand* (QR08, secondary care clinician, UHL).

To further improve quality of care, providers identified the intervention as a way to support shared working across primary and secondary care, promoting a team approach and providing an opportunity for collaborative working. One GP explained how the '*patient is in the middle'* (QR23) and a secondary care clinician felt that '*we don't sing off the same hymn sheet'* (QR08). One GP explained that w*e don't ever see secondary care specialists* and a secondary care clinician explained *there's always this assumption that the GP will sort it out* (QR13). It was acknowledged that shared working provides an opportunity to understand each other's roles, to manage expectations and to learn from one another. Providers went on to report patient benefits in terms of access to specialist clinicians and working as a multi-disciplinary team, *I've always found that patients who have the opportunity to talk to multiple members of the medical team rather than a doctor or a nurse come out with better outcomes* (QR08, secondary care clinician, UHL).

Patients also spoke about the benefit of joined-up working, with one person stating;

> *…you attend lots of different clinics, you've got different people involved in your healthcare. It's not just one thing and everything's linked… it's what they call whole person care. It's not just your blood pressure, it's also the vascular team, it's the podiatrist, it's the diabetes specialist* (QR07, out-patient clinic, GSTT).

Theme 2: A solution to address the gap in no man's land

All participant groups spoke about a need to target patients earlier and identified a service provision gap in preventative medicine and early diagnosis. One secondary care clinician stated;

> *I think, almost targeting people earlier on, so claudicants would be really good… claudicants at the very first stage of their peripheral arterial disease, they're not really managed in secondary care, but they're also not really managed in primary care… this gap where they're in no man's land and they almost wait until it's severe enough to managed into secondary care* (QR18, secondary care clinician, UHL).

For peer review only - http://bmjopen.bmj.com/site/about/guidelines.xhtml

Patients explained how it took time to be referred to secondary care, and in one person's situation, it became an emergency;

> *…my doctor prescribed pills and some cream for my foot but they didn't do anything. And then it was only when I went back to podiatry after three months of constant pain, it was a case of just wait and see how things went… I went one day in the November, and they said you need to have your toe off almost immediately… they sent me to [hospital] and [hospital] say 'your toe needs to come off and it needs to come off in 20 minutes time'* (QR04, In-patient care, UHL).

Difficulties with patients presenting late, high secondary care referral thresholds resulting in delayed referrals to secondary care and a wait for secondary care to make the diagnosis, alongside lack of support for primary care to commence diagnostics in the community were also listed as barriers to accessing timely care. Some secondary care clinicians felt that the care of those with IC should be managed in primary care.

> *I think even the way that if the GP could start the patient, the first medical therapies, like, clopidogrel and statins, if he also could start the patient on exercise therapies. So, it should be implemented at the same kind of wave of the first medical therapy.* (QR19, secondary care clinician, UHL).

GPs offered their support and made a request to do more sooner. Some suggested using the intervention in general practice as part of a PAD prevention programme. They acknowledged the value of using the intervention to promote earlier conversations with patients, manage patient expectations, and support primary care to start best medical therapy. GPs also spoke about how being involved sooner, and completing the clinical checklist, could be used to support triage of referrals to secondary care. GPs noted that preventative medicine and health promotion is core to their practice, and that sharing information across services and with patients reinforces the message and supports implementation.

Theme 3: Prioritising and making it happen

Secondary care clinicians spoke about the need to prioritise the intervention alongside limited resources and the burden of work. One secondary care clinician explained, *it shouldn't be seen as an extra thing* (QR08). Individual levels of confidence, attitude, knowledge and experience of the LEGS bundle, or a similar checklist/patient education intervention influenced

For peer review only - http://bmjopen.bmj.com/site/about/guidelines.xhtml

prioritisation with some staff identifying checklists as a quick and easy-to-use memory prompt. However, another reported that;

> *…people feel that it's just a tick-box exercise rather than a safety mechanism, and therefore no engagement with it… [it's] another piece of paper… I think part of the challenge is engagement with clinicians. Clinicians don't like threats to their autonomy… doctors, surgeons in particular are reticent, or certainly some are reticent to engage with checklists because they feel that it is doubling down or they already know this or that, you know, this simplifies things* (QR13, secondary care clinician, UHL).

Secondary care clinicians spoke about the extra resources required to deliver the intervention; it takes time to build relationships and to have conversations with patients. One secondary care clinician stated that finding the time to talk would require extra staff, this in an already stretched environment with staff shortages and long waiting times.

> *That will take more time, to make it personalised, to make it meaningful… you could almost imagine that they have a consultation and then some of those patients might benefit from sitting down with one of the specialist nurses… you could almost benefit from a 20-minute appointment after the standard clinic appointment to go through all of that. But again, that's a resource burden, that means that you've got to have someone extra in clinic* (QR13, secondary care clinician, UHL).

Secondary care clinicians felt that the intervention fits the in-patient environment neatly and could be used as a record of the out-patient clinic consultation. GPs felt that it could support the complexity of assessing patients remotely in the community. The need for training was recognised;

> *I think there is a huge lack in primary care education, and I think that goes to show by the starting people coming in are only on like a 40% of them are on Best Medical Therapy so I think a big thing to target would be primary care to inform them* (QR18, secondary care clinician, UHL).

GPs went on to request that all primary care staff be involved and not just GPs, and that the primary care training be delivered by the vascular team.

Theme 4: Personalised information and supportive conversations for taking on the advice

Providers and patients spoke about the delivery of the intervention, recognising the importance of offering personalised care and tailoring patient information to individual needs. Secondary

For peer review only - http://bmjopen.bmj.com/site/about/guidelines.xhtml

care clinicians felt that the intervention should be adapted to identify realistic targets and individual goals, *or the advice rings hollow* (QR13), and to offer a different approach for each patient. Staff reported;

> *I think the hard thing as well is that with the LEGS bundle, sometimes you're giving it to someone who's just had an amputation or about to have an amputation so it's just to be mindful you're talking about things like exercise they look at you very disparagingly and that can be a bit tricky… I think it's a really good checklist and bundle for claudicants and I think that's where it really shines but I think for when you get to the stage of CLTI, I think those individuals often feel that they're too far gone for you to talk to them about lifestyle modification and things like that* (QR18, secondary care clinician, UHL).

Patients also spoke about the importance of timing in terms of when they received the lifestyle information.

> *If I was a bit younger and I'd got more get-up-and-go, I would gladly have a leaflet and go through it and take up advice and what have you. But as I am at the moment, my health is far from good* (QR14, in-patient care, UHL).

> *…it is a bit too late in that respect so I can only do what I can do. I mean yesterday I was out in the garden doing some weeding but I have to use a seat to do it* (QR05, in-patient care, UHL).

Patients explained that they like to receive information face-to-face and suggested that the education leaflet could be used as a supportive conversation guide. They emphasised the importance of the conversation that goes alongside the advice and information, and the relationship that they have with their health professional(s) in terms of asking questions and being able to talk about making changes like smoking cessation. One patient explained,

> *… the only thing I've got to do is drink and smoke and watch the tv – cause if I didn't have those three things, I would be pulling my hair out… to be quite honest, I'm quite happy at the moment to be carrying on the way I am. It might sound silly to you, but I enjoy smoking. I love a drink, I like my telly… if you're brow-beaten into packing up when you don't really want to, all this, it can have a great effect on people* (QR14, in-patient care, UHL).

GPs and secondary care clinicians acknowledged the need to support patient preference, to involve patients in the conversation and to share decision making.

> *…you have to strike a balance, and therefore taking the patient's preferences into account is important. We assume that patients want to live the longest life they can, I'm not sure that's always correct. Some patients would rather accept that they live a slightly shorter life but with the benefit of enjoying certain things that we all know are less healthy* (QR13, secondary care clinician, UHL).

For peer review only - http://bmjopen.bmj.com/site/about/guidelines.xhtml

It was acknowledged that everyone knows they need to stop smoking and it is easy to bombard people with information. Solutions such as motivational interviewing techniques were identified by one GP as a way to support engagement conversations. Secondary care clinicians felt that providing information on the benefits of following the advice versus taking pleasure away might help. For others, frank conversations that explain the risks help;

> *I think honesty is the most important aspect of this and bringing people in front of the data, highlighting the data, and actually not sugar coating anything. We know, for example, that if you have critical ischemia, so severe peripheral arterial disease, if your prognosis is worse, your five-year survival is worse than some kinds of cancer. So, if you tell someone that unless you actually take this intervention seriously, your likelihood of not being around in five years is very high. I know it sounds bleak, but it may be the wake-up call that someone needs. Or explaining that from the moment you have severe arterial peripheral disease, the likelihood of you losing your leg and being wheelchair bound may be an important thing to highlight as opposed to speaking in a circumspect way. I think just being honest and explaining the risks may be a way of engaging patients.* (QR15, secondary care clinician, UHL).

A patient explained how the honest frank information helped;

> *The doctor actually threatened me. He said to me, if I'm going to do this operation, you got to stop smoking. So, I did. That helped, or I could lose my leg. I thought it was brilliant the way [the doctor] come out with it and I've never stopped thanking him for it. It works* (QR06, out-patient care, GSTT).

Discussion

The aim of the study was to provide an understanding of the feasibility and implementation of the LEGS (LEaflet Gp letter Structured checklist) intervention, a complex intervention in the form of a healthcare bundle, designed to improve the care of patients with PAD in the NHS. Our inductive approach to analysis found four overarching themes: i) *the potential to make a difference, ii) a solution to address the gap in no man's land, iii) prioritising and making it happen* and *iv) personalised information and supportive conversations for taking on the advice.* These themes provide an overarching message of acceptability for the LEGS intervention by highlighting benefits at the individual and system-level and by outlining strategies for increasing impact and uptake. We used the Consolidated Framework of Implementation Research (CFIR) to guide our analysis, and all five CFIR domains are

For peer review only - http://bmjopen.bmj.com/site/about/guidelines.xhtml

represented (13). The CFIR is well-placed for developing a model for implementing secondary care-initiated treatment in primary care (17).

All participants spoke positively about using the intervention to make a difference to patient care, as an opportunity to meet patient needs, and as a way to enhance the quality of PAD care through improved working practices. This promotes a streamlined patient pathway from primary care to secondary care and to shared care. This reflects the value of an agreed pathway of care and shared partnership working that provides an opportunity for the intervention to be delivered as part of a wider team and to prioritise patient-centred care (17).

There was a consensus of optimism in participant accounts about the potential to make a difference. While barriers to seeking care for those with PAD are well-documented, (18, 19) the findings confirm that the period between diagnosis of early-stage PAD (e.g., claudication) and subsequent potential diagnosis of advanced PAD with severe symptoms, is a time of no man's land, when patients do not receive any help, education, support, or appropriate medication to address their condition (9). As such, the benefits of the LEGS intervention were identified in terms of meeting the needs of this subgroup of patients, and to address the gap in care. GPs were optimistic that they can deliver the LEGS intervention with training and ongoing sustained communication with secondary care. The findings highlight the need to consider training requirements and systems for maintaining open primary-secondary care communication to support implementation. For example, education on tailoring the intervention for patients at both early and late stages of PAD, that can be used across primary and secondary care settings, similar to the model used with other chronic conditions (17, 20) Improving the awareness of PAD among healthcare providers and supporting patients to take responsibility for their own health are therefore important determinants in the success of implementation (21).

For peer review only - http://bmjopen.bmj.com/site/about/guidelines.xhtml

While the three elements of the LEGS intervention have been designed to facilitate interaction between secondary and primary care following diagnosis of PAD, the intervention focus until now, has been on secondary care services, that is, hospital admission or out-patient clinic. However, our findings indicate that the intervention can be used to address the gap in service provision for patients who do not yet meet the referral criteria for secondary care services. We therefore propose to extend the use of the LEGS intervention across the primary-secondary care interface. Our findings also confirm the need for individualised patient care, supportive communication and shared decision making in order to take on the advice in a more effective way (22). The finalised LEGS intervention should therefore promote supportive conversation between patient and healthcare staff, both in primary and secondary care settings. The open approach to changing lifestyle and behaviour could be built on techniques like motivational interviewing, which is already commonly used in primary care practice (23). Lastly, our findings confirm that healthcare providers are optimistic and positive about the introduction of an intervention which will help them to deliver more consistent and guideline-based PAD treatment. The challenge now is to take this enthusiasm together with the lessons learned from this qualitative research to adapt the LEGS intervention before wider testing and adoption in the NHS. The main changes will relate to usability in primary care after diagnosis of early-stage PAD, facilitation of effective communication not only between primary and secondary care staff, but also patients and staff, as well as focussing on specific needs of patients by prioritising areas to focus on in an incremental or stepwise manner.

Strengths and limitations

The paper adds new knowledge on the development and implementation of a community and hospital intervention for PAD. It is important to note that the findings are specific to the CHABLIS study. It is possible, however, to enhance transferability by describing the research context and assumptions, and by making connections between the analysis of participants accounts and claims in the extant literature.

For peer review only - http://bmjopen.bmj.com/site/about/guidelines.xhtml

While the sample size for this study may seem small, the volume of interview data we collected is unique for this patient population. Many factors influence the success of recruiting participants in research and engaging vulnerable patient populations brings additional complexity (24). PAD is a chronic and progressive disease with burdensome symptoms including physical disability, cardiovascular risks and mortality (25). Those who live with PAD are vulnerable because they are living with extreme frailty, often with the threat or result of limb loss, and with risks associated with poorer socio-economic conditions (26). Alongside, patients with PAD might be less receptive to engaging with research because they experience specific barriers to accessing healthcare meaning that taking part in research alongside managing health care and health needs might be considered onerous (19).

A strength is the focus on rigor and triangulation of participant perspectives. We also acknowledged the complexity of intervention implementation across the secondary-primary care interface and therefore used a systems research perspective for addressing implementation at the outset of the research. We used implementation theory to guide analysis and to provide a layered coding approach, to ensure that intervention and implementation recommendations are novel, grounded in the data, and useful (13).

While the anonymity of telephone use can allow participants to disclose sensitive information, telephone interviews have received criticism for compromising interviewer/participant rapport and interaction, and for limiting contextual data due to the absence of face-to-face contact and visual cues. However, this method of data collection is convenient, in that it is flexible (in terms of time and location), accessible (i.e., remote research conducted during the COVID-19 pandemic), and allows for a wide reach (e.g., accessing a diverse population from both urban and rural areas) (27).

Conclusions

For peer review only - http://bmjopen.bmj.com/site/about/guidelines.xhtml

The LEGS intervention can be tailored for use at early and late stages of PAD, can be provided across primary and secondary care settings, and provides an opportunity to promote shared working across the primary-secondary care interface. These findings are important to the specifics of the CHABLIS study but can also go some way in informing the wider learning for other interventions that are implemented across the primary-secondary care interface.

Funding

This article presents independent research funded by National Institute for Health Research for Patient Benefit (RfPB) Programme (funder's reference: NIHR202008). The views expressed are those of the author(s) and not necessarily those of the NIHR or the Department of Health and Social Care.

Competing interests

The authors declare that they have no competing interests.

Authors' contributions

CL contributed to the qualitative study design, coordinated the study, led data collection and analysis, and drafted the manuscript. AS is principal investigator to the CHABLIS study, acquired funding, participated in the study design, contributed to data collection, and drafted the manuscript. PS contributed to data collection and reviewed the manuscript. RB and BB drafted the manuscript. EW contributed to data collection and reviewed the manuscript. VL designed the qualitative study, provided oversight on study coordination, data collection and analysis, and drafted the manuscript. All authors read and approved the final manuscript.

Consent for publication

Consent for publication has been obtained from all research participants.

Word count

4,775

For peer review only - http://bmjopen.bmj.com/site/about/guidelines.xhtml

Availability of data and materials

This study uses data (containing potentially identifying and/or sensitive information) collected from a small group of staff participants and a vulnerable patient population, and involves indirect identifiers (such as sex, ethnicity, location, etc.) that may risk the identification of study participants. Sharing data outside of the anonymised excerpts and quotations included in the paper will violate the agreement to which the participants consented.

Acknowledgements

We would like to thank all patients, secondary care staff and GPs who participated in this study and who generously gave their time and honest thoughts. We would like to thank the local research teams for their support in recruiting to this qualitative study and we are also grateful to the NIHR Clinical Research Network Research Delivery Managers that helped to recruit GP participants. We would also like to thank the stakeholder PPI group for their time and input to the CHABLIS study.

Ethics Statement

The research was reviewed and approved by the NHS Wales Research Ethics Committee (REC), reference number20/WA/0319 and the NHS Health Research Authority (HRA).

References

1. Herrington W, Lacey B, Sherliker P, Armitage J, Lewington S. Epidemiology of Atherosclerosis and the Potential to Reduce the Global Burden of Atherothrombotic Disease. Circ Res. 2016;118(4):535-46.

2. Cea-Soriano L, Fowkes FGR, Johansson S, Allum AM, García Rodriguez LA. Time trends in peripheral artery disease incidence, prevalence and secondary preventive therapy: a cohort study in The Health Improvement Network in the UK. BMJ Open. 2018;8(1):e018184.

3. Dhaliwal G, Mukherjee D. Peripheral arterial disease: Epidemiology, natural history, diagnosis and treatment. Int J Angiol. 2007;16(2):36-44.

4. Parvar SL, Fitridge R, Dawson J, Nicholls SJ. Medical and lifestyle management of peripheral arterial disease. J Vasc Surg. 2018;68(5):1595-606.

For peer review only - http://bmjopen.bmj.com/site/about/guidelines.xhtml

For peer review only

5. Nehler MR, Duval S, Diao L, Annex BH, Hiatt WR, Rogers K, et al. Epidemiology of peripheral arterial disease and critical limb ischemia in an insured national population. J Vasc Surg. 2014;60(3):686-95.e2.

6. Dua A, Lee CJ. Epidemiology of Peripheral Arterial Disease and Critical Limb Ischemia. Tech Vasc Interv Radiol. 2016;19(2):91-5.

7. Morley RL, Sharma A, Horsch AD, Hinchliffe RJ. Peripheral artery disease. Bmj. 2018;360:j5842.

8. National Institute for Health and Care Excellence. Peripheral arterial disease: diagnosis and management. 2020.

9. Saratzis A, Jaspers NEM, Gwilym B, Thomas O, Tsui A, Lefroy R, et al. Observational study of the medical management of patients with peripheral artery disease. Br J Surg. 2019;106(9):1168-77.

10. Skivington K, Matthews L, Simpson SA, Craig P, Baird J, Blazeby JM, et al. A new framework for developing and evaluating complex interventions: update of Medical Research Council guidance. Bmj. 2021;374:n2061.

11. Tong A, Sainsbury P, Craig J. Consolidated criteria for reporting qualitative research (COREQ): a 32-item checklist for interviews and focus groups. Int J Qual Health Care. 2007;19(6):349-57.

12. Braun V, Clarke V. Using thematic analysis in psychology. Qualitative Research in Psychology. 2006;3(2):77-101.

13. Damschroder LJ, Aron DC, Keith RE, Kirsh SR, Alexander JA, Lowery JC. Fostering implementation of health services research findings into practice: a consolidated framework for advancing implementation science. Implement Sci. 2009;4:50.

14. Braun V, Clarke V. One size fits all? What counts as quality practice in (reflexive) thematic analysis? Qualitative Research in Psychology. 2021;18(3):328-52.

15. QSR International Pty Ltd. NVivo. 2018.

16. Bammer G. Key issues in co-creation with stakeholders when research problems are complex. Evidence & Policy. 2019;15(3):423-35.

17. Le Boutillier C, Ahmed H, Patel V, McPhail M, Carter B, Ward C, et al. General Practitioner perspectives on factors that influence implementation of secondary care-initiated treatment in primary care: exploring implementation beyond the context of a clinical trial. PLoS One. 2022.

18. Meffen A, Houghton JSM, Nickinson ATO, Pepper CJ, Sayers RD, Gray LJ. Understanding variations in reported epidemiology of major lower extremity amputation in the UK: a systematic review. BMJ Open. 2021;11(10):e053599.

19. Tan TW, Crocker RM, Palmer KNB, Gomez C, Armstrong DG, Marrero DG. A qualitative study of barriers to care-seeking for diabetic foot ulceration across multiple levels of the healthcare system. J Foot Ankle Res. 2022;15(1):56.

20. Smith SM, Cousins G, Clyne B, Allwright S, O'Dowd T. Shared care across the interface between primary and specialty care in management of long term conditions. Cochrane Database Syst Rev. 2017;2(2):Cd004910.

21. Bridgwood BM, Nickinson AT, Houghton JS, Pepper CJ, Sayers RD. Knowledge of peripheral artery disease: What do the public, healthcare practitioners, and trainees know? Vasc Med. 2020;25(3):263-73.

22. Barry MJ, Edgman-Levitan S. Shared decision making--pinnacle of patient-centered care. N Engl J Med. 2012;366(9):780-1.

For peer review only - http://bmjopen.bmj.com/site/about/guidelines.xhtml

23. Rubak S, Sandbaek A, Lauritzen T, Christensen B. Motivational interviewing: a systematic review and meta-analysis. Br J Gen Pract. 2005;55(513):305-12.

24. Le Boutillier C, Snowdon C, Patel V, McPhail M, Ward C, Carter B, et al. Using a theory-informed approach to explore patient and staff perspectives on factors that influence clinical trial recruitment for patients with cirrhosis and small oesophageal varices. PLoS One. 2022;17(2 February).

25. Adam DJ, Beard JD, Cleveland T, Bell J, Bradbury AW, Forbes JF, et al. Bypass versus angioplasty in severe ischaemia of the leg (BASIL): multicentre, randomised controlled trial. Lancet. 2005;366(9501):1925-34.

26. Brostow DP, Hirsch AT, Kurzer MS. Recruiting older patients with peripheral arterial disease: evaluating challenges and strategies. Patient Prefer Adherence. 2015;9:1121-8.

27. Irvine A. Duration, Dominance and Depth in Telephone and Face-to-Face Interviews: A Comparative Exploration. International Journal of Qualitative Methods. 2011;September:202-20.

For peer review only

Interview Topic Guide for Patients

Title of the study: A **C**ommunity and **H**ospital c**A**re **B**undle to improve the medical treatment of c**L**aud**I**cation and critical limb i**S**chaemia. **(**The CHABLIS study)

Welcome and introduction

[Seek consent to continue and to audio-record the interview (if applicable). Participant has to provide written informed consent (see separate form). Let them know that no personal identifiable data will be recorded and a participant number will be allocated to them.]

The main aim of this study is to assess whether a dedicated intervention (a care bundle) can be used in the routine care of patients who present with symptomatic Peripheral Arterial Disease (PAD). The care bundle includes a number of checklists and leaflets for doctors and patients. The intervention is aimed at reducing the risk of major cardiovascular events (i.e. heart attacks and strokes), and preventing amputation, by making sure patients with PAD receive the appropriate treatment and management as recommended by national guidelines. We have called this intervention LEGS (the LEGS bundle), given that the aim is to save legs from amputation and improve vascular care.

The LEGS intervention:

ELEMENT 1: In-patient checklist/Outpatient Checklist

The doctors who see you will be given a checklist of the common things that patients with PAD should be offered in terms of medications and / or tests. All of these suggestions are already part of what is the standard NHS care for PAD. Using your medical notes, we will record your blood pressure, your medications (current), you medical history, previous surgeries, imaging results, your routine blood test results (cholesterol, lipid profile, full blood count), smoking status, and demographic information (age, height, weight, gender). This is standard information that the NHS already collects in order to be able to offer you treatment for your blocked arteries.

ELEMENT 2: 2nd (follow-up) letter to the GP

ELEMENT 3: Patient & carers' leaflet

You will be given a leaflet which will provide you with information about PAD and other things you can do to help reduce your risks.

You will have an appointment to see us again in six months to check your legs and your overall health. This is standard for someone with PAD. We will again record your blood pressure, your medications (current), your medical history, previous surgeries, imaging results, your routine blood test results (cholesterol, lipid profile, full blood count), smoking status, and demographic information (age, height, weight, gender). During that appointment we will ask you to fill in a questionnaire which assesses how well people are taking their prescribed medication, called the MARS questionnaire.

For peer review only - http://bmjopen.bmj.com/site/about/guidelines.xhtml

The interview will explore your experience of receiving the intervention. We will ask about your opinion of NHS care relating to your leg artery problems in greater detail. We will ask you how you have found the management of your care at hospital and your GP practice, what your thoughts are of the information leaflet, and if you think it has been of any benefit to you or not.

Questions:

1. How have you found the management of your care at hospital and your GP practice?

- the initial consultation between patient and healthcare professional after the diagnosis of peripheral arterial disease has been made
  - what should be covered in the initial consultation?
  - Anything missing from this consultation?
  - Are you able to ask questions?

- the frequency and duration of follow-up

2. What are your thoughts on the information leaflet?

- Is there enough information about peripheral arterial disease? Too much information? Is the information clear? Signposts for where to go for more information?
- Is there anything on the leaflet that you don't understand or would like more information about?
- What would you like to see included in a leaflet designed to inform patients about peripheral arterial disease care and therapies?
- What about the length of the leaflet?

3. Do you think it has been of any benefit to you or not?

4. In your opinion, are there any factors that might influence someone's ability to follow the guidance in the patient information leaflet? (i.e. barriers to taking medication, managing diet)

- Is there anything that could help? Solutions to any barriers for following the advice?

5. Any ideas for improving any of the elements of the intervention?

   ELEMENT 1: Inpatient/outpatient checklist (& patient consultation)
   ELEMENT 2: 2nd (follow-up) letter to the GP (& patient consultation)
   ELEMENT 3: Patient & carers' leaflet

6. What do you need for your health to improve? (wider experiences and perceived needs)

Anything not covered? Is there anything that we haven't covered in the interview that you think we should know or think about?

Closing and thanks - check that the participant is still happy for you to use all the information provided and offer the possibility to erase sections of the recording.

For peer review only - http://bmjopen.bmj.com/site/about/guidelines.xhtml

Thank them for their time and contribution.

Interview Topic Guide for Secondary Care Clinicians & GPs

Title of the study: A **C**ommunity and **H**ospital c**A**re **B**undle to improve the medical treatment of c**L**aud**I**cation and critical limb i**S**chaemia. **(**The CHABLIS study)

Welcome and introduction

[Seek consent to continue and to audio-record the interview (if applicable). Participant has to provide written informed consent (see separate form). Let them know that no personal identifiable data will be recorded and a participant number will be allocated to them.]

The main aim of this study is to assess whether a dedicated intervention (a care bundle) can be used in the routine care of patients who present with symptomatic Peripheral Arterial Disease (PAD). The care bundle includes a number of checklists and leaflets for doctors and patients. The intervention is aimed at reducing the risk of major cardiovascular events (i.e. heart attacks and strokes), and preventing amputation, by making sure patients with PAD receive the appropriate treatment and management as recommended by national guidelines. We have called this intervention LEGS (the LEGS bundle), given that the aim is to save legs from amputation and improve vascular care.

ELEMENT 1: In-patient checklist/Outpatient Checklist

ELEMENT 2: 2nd (follow-up) letter to the GP

ELEMENT 3: Patient & carers' leaflet

The interview will explore your experiences/perspectives of delivering the intervention. This will help us to evaluate the ability/feasibility of staff in delivering the intervention, overall impression, validity, usability, acceptability and engagement with the intervention. It will include barriers to engagement or delivery and ideas for improving the intervention.

This is a GUIDE for topics. Further topics will and can be explored if necessary.

The purpose of the study is to find out about staff views and experiences of implementing the LEGS intervention as a part of routine clinical care. This provides us with information on factors that facilitate or hinder implementation and on possible solutions to overcome any identified barriers.

Key objectives of the interview session: i) to identify barriers to and facilitators of each of the key intervention targets; ii) identify issues around delivery, training needs and competencies.

We would like to ask you about what helps and what hinders success in implementing the LEGS intervention as a part of routine clinical care.

- To get started, we would like to ask about your views on existing NICE guidance and factors that might influence implementation of NICE guidance on cardiovascular risk reduction amongst patients with peripheral arterial disease. Please comment on: i)

For peer review only - http://bmjopen.bmj.com/site/about/guidelines.xhtml

prescribing, ii) awareness of NICE guidance by primary and secondary care, iii) adherence by patients (when they have been prescribed their medication), iv) follow-up practices in primary and secondary care.

- What do you think about using the LEGS intervention with this group of patients? Are there any benefits? Or challenges?

Understanding implementation

Aims:
1) To explore what participants think helps or hinders implementation.

2) To explore what participants' identify as potential solutions to implementation barriers.

What helps implementation?

- What factors do you perceive might support implementing the LEGS intervention in routine clinical practice?
- What helps/would help you (and your colleague) to provide the intervention a part of routine clinical care?
- What are your views on the LEGS intervention and the acceptability and feasibility of delivering the intervention?
- Do you feel that you have the knowledge and skills to deliver this intervention?
- What implementation strategies might help? Training?

What prevents or hinders implementation?

- What prevents or hinders success in implementing the LEGS intervention as a part of routine clinical care?
- Are there/or might there be challenges around implementation?
- What would further support you to implement the intervention?

Prompts / Continuation Questions:

- What resources would better equip you to implement the intervention?
- What would you say is the ideal environment to implement the LEGS intervention in?

Implementation solutions

- What solutions would you recommend to address these identified barriers?
- Any other influences on implementation? COVID? Other clinical priorities?
- Any changes needed to the intervention? Ideas for improving the intervention?

  (Can you advise us on the 3 most important things you would like to see covered in a patient leaflet for patients with peripheral arterial disease? Can you please advise us

For peer review only - http://bmjopen.bmj.com/site/about/guidelines.xhtml

UNIVERSITY OF LEICESTER

KING'S College LONDON

on the 3 most important things you would like to see covered in a checklist which supports the delivery of peripheral arterial disease care for patients?)

This interview was designed to help us understand the extent to which the CHABLIS intervention has been implemented, and what has helped or hindered you in doing this. Are there any other important points that you would like to discuss before we close the discussion?

Closing and thanks - check that the participant is still happy for you to use all the information provided and offer the possibility to erase sections of the recording.

Thank them for their time and contribution.

Ensure that they have received a PIS and the CF has been handed back for photocopying.

For peer review only

For peer review only - http://bmjopen.bmj.com/site/about/guidelines.xhtml

CFIR Domains and the LEGS bundle	Patients	Secondary care clinicians	GPs
**THE LEGS BUNDLE**			
Element 1: Clinical checklist		X	X
Element 2: Patient and carer education leaflet	X	X	X
Element 3: GP action letter	X	X	X
**CFIR DOMAINS**			
**Domain 1: INTERVENTION CHARACTERISTICS**	**X**	**X**	**X**
**1.1 Intervention source:** Perception about whether the intervention is externally or internally developed.			
The whole bundle		X	X
I sat down with the developers, and they gave me the background		X	
Perception about how the intervention was developed			X
Involving stakeholders in the development process will promote uptake			X
**1.2 Evidence strength and quality:** Perceptions of the quality and validity of evidence supporting the belief that the intervention will have desired outcomes.			
Clinical checklist		X	
Built on existing NICE guidance		X	
The guidance is vague		X	
The guidance is clear, but we don't follow it		X	
**1.3 Relative advantage (or not):** Perception of the advantage of implementing the intervention versus an alternative solution.			
The whole bundle		X	X
Improves care and outcomes		X	
Standardises a minimum quality of care		X	
Allows us to make a difference to patients		X	
Joins up primary and secondary care working			X
Improves awareness of NICE guidance in primary & secondary care		X	X
Improves awareness and understanding of PAD		X	X
Clinical checklist		X	
Builds your relationship with the patient		X	
Improves medication compliance		X	
Patient and carer education leaflet	X	X	X
Patients are empowered by being informed		X	
I don't want any more leaflets	X		
District nurses can answer my questions	X		
A conversation guide for primary care			X
Existing relationships to reinforce the message			X
GP action letter		X	X
A standardised letter with clear information		X	
Useful for non-medical led community vascular clinics			X
Repeat GP action letter is a duplicate, adds to the workload and sets the wrong tone			X
It gives the impression that secondary care do not trust GPs			X

For peer review only

CFIR Domains and the LEGS bundle	Patients	Secondary care clinicians	GPs
**1.4 Adaptability:** The degree to which an intervention can be adapted, tailored, refined, or reinvented to meet local needs.			
Clinical checklist		X	
Adapting to the environment		X	
Fits the in-patient environment neatly		X	
Incorporate into a universal admission checklist		X	
Limited access to blood pressure monitoring or cholesterol tests in out-patient clinic		X	
Promote checklist in primary care to be used alongside secondary care referral letter		X	
Patient and carer education leaflet	X	X	X
Offer personalised care and tailor patient education to individual needs		X	X
A different approach for each type of patient			X
Different information for each patient			X
Existing info most likely to suit those with intermittent claudication			X
Offer realistic targets or the advice rings hollow		X	
Consider co-exiting morbidities			X
Too much information	X		
Change information layout to provide summary at outset		X	
Missing information	X		X
Understanding what causes PAD and preventing further blockages	X		X
Managing the psychological impact of PAD	X		
Maintaining movement and physiotherapy input	X		
Impact on work, social and family life	X		
Acknowledgement of reliance and impact on family	X		
Carer and family support	X		
Managing future risks and when to seek support		X	X
**1.5 Trialability:** The ability to test the intervention on a small scale in the organisation, and to be able to reverse course (undo implementation) if warranted.			
Clinical checklist	X	X	
A quick quality check		X	
Patient and carer education leaflet	X	X	
Leaflet is a resource for referring back	X	X	
**1.6 Complexity:** Perceived difficulty of implementation, reflected by duration, scope, radicalness, disruptiveness, centrality, and intricacy and number of steps required to implement.			
Clinical checklist		X	X
Simplicity		X	
Improvements that make it even simpler		X	
It needs to be a bit more, no and why		X	X
GP action letter	X	X	X
A template for new staff		X	
A time-consuming manual coding process with potential for error			X
Waiting times for primary care/test appointments	X		X
Difficult to assess patients remotely			X

For peer review only - http://bmjopen.bmj.com/site/about/guidelines.xhtml

CFIR Domains and the LEGS bundle	Patients	Secondary care clinicians	GPs
Lack of continuity			X
60 calls and 40 letters a day			X
Up to three months to receive hospital letters			X
Patients to hand deliver letter to GP surgery			X
**1.7 Design quality and packaging:** Perceived excellence in how the intervention is bundled, presented, and assembled.			
Clinical checklist		X	
Working across two different systems - Paper and electronic		X	
Paper checklists tend to get missed		X	
Electronic option required		X	
Patient and carer education leaflet	X	X	X
Accessibility	X		
Stored in a safe place	X		
Lost the leaflet	X		
Would be improved if available by email	X		
Presentation & format	X		
Paper leaflet		X	
Larger print has been requested		X	
Language can be hard to understand	X		X
Write for a lower reading age and provide additional information or additional resources for people who are able to access more detailed information			X
Keep it simple, give less information, in different formats and in a way that is understandable			X
Avoid didactic language and reframe the approach			X
There's a lot of 'do nots'			X
Ensure accuracy of information			X
Confusing dietary advice			X
Provide more infographics and pictures	X	X	X
Include diversity in visuals (e.g., pictures of leg ulcers/skin changes in different skin colours)		X	
Add web links			X
Could be supported by existing NHS software like QRX, direct information to patient phones			X
Offer an audio and video version	X	X	X
Patient information to be offered as a group intervention	X	X	
Opportunity for group to be peer-led		X	
GP action letter			X
Same information and same system across primary and secondary care			X
Include information on the discussion around contraindications and side effects of any newly prescribed medication with patient			X
Any contraindications primary care should be aware of, useful for clarification			X
Offered and accepted/declined smoking cessation referral			X
Provide a clear plan for if the situation deteriorates			X
Copy of letter to patient – shared message – reinforcement			X

For peer review only

CFIR Domains and the LEGS bundle	Patients	Secondary care clinicians	GPs
**1.8 Cost:** Costs of the intervention and costs associated with implementing that intervention including investment, supply, and opportunity costs.			X
It is not commissioned or financially viable			X
Primary care time			X
Maintaining equipment			X
**Domain 2: OUTER SETTING**	X	X	X
**2.1 Patient needs and resources:** The extent to which patient needs, as well as barriers and facilitators to meet those needs are known and prioritised.			
The whole bundle	X	X	X
Working as part of a whole team			X
Promoting joined up working across primary and secondary care		X	X
Enabling specialist care from different people			X
Expectations and understanding of each other's roles			X
Timely access back into secondary care if needed			X
Secondary care oversight			X
Accessible referral pathway	X		X
We can't make a referral anyway			X
Secondary care referral thresholds too high			X
You don't fit the criteria so wait until it gets worse			X
Delayed referral to secondary care	X	X	X
Presenting too late		X	
Can I do some of the diagnostics in the community?			X
Time wastage		X	X
A geographical consideration	X		X
Locality of secondary care services	X		X
Transport needs	X		X
Clinical checklist		X	X
Use in primary care to start best medical therapy		X	
Would promote earlier conversations with patients in primary care		X	
Use at point of admission and discharge for in-patients		X	
Adapt for primary care to support referral			X
Patient and carer education leaflet	X	X	X
Patient education provided too late	X	X	
Too far gone to talk about lifestyle modification		X	
Level of functioning & mobility - I can only do what I can do	X		
It is crucial whenever it happens		X	
Introduced sooner in primary care as part of a PAD prevention programme			X
Informing patients to reduce the chance of presenting late/in crisis			X
Managing patient expectations			X
**2.2 Cosmopolitanism:** The degree to which an organisation is networked with other external organisations.			
The whole bundle		X	
Shared primary & secondary care working		X	
Patient and carer education leaflet			X

For peer review only

CFIR Domains and the LEGS bundle	Patients	Secondary care clinicians	GPs
Copy of leaflet to primary care to reiterate message			X
GP action letter		X	
Clear open communication across community and hospital services		X	
Joined up clinical record systems		X	
We don't sing off the same hymn sheet		X	
**2.3 External policies and incentives:** A broad construct that includes external strategies to spread interventions including policy and regulations (governmental or other central entity), external mandates, recommendations and guidelines, pay-for-performance, collaboratives, and public or benchmark reporting.			
The whole bundle		X	
Additional funding		X	
**Domain 3: INNER SETTING**	X	X	X
**3.1 Structural characteristics:** The social architecture, age, maturity, and size of an organisation.			
The whole bundle		X	X
Impact of staff shortages		X	
Reduced staffing due to covid		X	
Involving the multi-disciplinary team			X
GP action letter		X	X
Time delay between writing and getting to primary care		X	X
Patient and carer education leaflet	X	X	X
Being able to follow the advice	X	X	
Difficulty ordering repeat medication	X		
Relying on others for food shopping	X		
Availability of supervised exercise		X	
It's easy to bombard somebody with information			X
**3.2 Networks and communications:** The nature and quality of webs of social networks and the nature and quality of formal and informal communications within an organisation.			
The whole bundle	X	X	X
A team approach		X	
Building relationships			X
Clear communication			X
The patient is in the middle			X
Involve all multi-disciplinary professionals (including pharmacists, nurses, physios)		X	
Access to the vascular team or not	X		X
Advice and information	X		X
**3.3 Culture:** Norms, values, and basic assumptions of a given organisation.			
The whole bundle		X	
Target patients earlier		X	
The gap in no man's land		X	
**3.4 Implementation climate - tension for change:** The degree to which stakeholders perceive the current situation as intolerable or needing change.			
The whole bundle		X	X
Primary care needs to do more sooner		X	
Secondary care referral threshold is too high			X
**3.5 Implementation climate - Compatibility:** The degree of tangible fit between meaning and values attached to the intervention by involved individuals, how those align with individuals' own norms, values, and			

For peer review only

CFIR Domains and the LEGS bundle	Patients	Secondary care clinicians	GPs
perceived risks and needs, and how the intervention fits with existing workflows and systems.			
The whole bundle			X
Existing systems in place for primary care medication management and monitoring			X
Provide 28 days medication to allow for delay in seeing GP			X
Preventative medicine and health promotion is core to primary care practice			X
Ability to offer smoking cessation support			X
Local variation in set-up of smoking cessation, dietary advice, exercise therapy			X
No formal set-up in primary care to pick it up			X
GP relationship with patients			
Not commissioned			
Secondary care to initiate smoking cessation referral			X
National protocol for weight reduction			X
Existing relationships with Diabetes nurses/clinics			X
**3.6 Implementation climate - Relative priority:** Individuals' shared perception of the importance of the implementation within the organisation.			
Clinical checklist		X	
Take the opportunity		X	
It shouldn't be seen as an extra thing		X	
A threat to autonomy		X	
Checklists are tick-box exercises rather than safety mechanisms		X	
**3.7 Implementation climate - Learning climate**			
The whole bundle			X
Two-way learning			X
Secondary care can learn from this too			X
Holistic picture of medication profile, side effects, competing medication			X
Teams are not static so require ongoing access to training		X	X
**3.8 Readiness for implementation – leadership engagement:** Commitment, involvement, and accountability of leaders and managers with the implementation.			
The whole package		X	
Provide a named leader		X	
External oversight is preferable		X	
**3.9 Readiness for implementation – available resources:** The level of resources dedicated for implementation and on-going operations including money, training, education, physical space, and time.			
Clinical checklist		X	X
Checklists are quick		X	
Managing it alongside the burden of work		X	
The assumption that the GP will sort it – putting it back into GP land		X	X
Lack of access to testing equipment in community			X
Limited community workforce (i.e., reduced number of district nurses) capacity for conducting ABPIs			X
Secondary care are gatekeepers for ABPIs and duplexes			X

For peer review only

CFIR Domains and the LEGS bundle	Patients	Secondary care clinicians	GPs
Waiting for secondary care to make diagnosis			X
Patient and carer education leaflet		X	
Time to talk requires extra staff		X	
GP action letter		X	X
Time burden and impact of dictating long clinic letters		X	
Joining it up - We've done our bit and now pass it on to the GP			X
Routine practice or adding to the workload			X
Feels top down and with didactic language			X
Keep the GP onside and change phraseology			X
**3.10 Readiness for implementation - access to information and knowledge:** Ease of access to digestible information and knowledge about the intervention and how to incorporate it into work tasks.			
The whole bundle		X	
Training on the purpose of the intervention		X	
Written information for those unable to attend training		X	
Prioritise training and supervision for junior staff – the people who do the day-to-day work		X	
Consider involving medical students		X	
Highlight the link between PAD and cardiovascular death		X	
Education on managing CLTI and Non-CLTI patients in primary care		X	
Knowing the risk factors for CLTI and how to make a referral to secondary care		X	
Taking an ABPI in primary care		X	X
Support the triage for secondary care			X
We're not commissioned to do it			X
Visibility of vascular teams at primary care conferences		X	
Learning on the job		X	X
Informal training to colleagues i.e., when seeing a medical outlier		X	
Use available supervision/debriefing opportunities			X
Webinar-ed out		X	
Training delivered by vascular team to primary care			X
Collaborative working and building relationships			X
We don't ever see secondary care specialists			X
Involve all primary care staff and not just GPs			X
Learning gap in exercise and lifestyle modification			X
**DOMAIN 4: CHARACTERISTICS OF INDIVIDUALS**			
The whole bundle	X	X	X
Taking on the advice	X	X	X
Familiarity and understanding of PAD		X	X
**4.1 Knowledge and beliefs about the intervention**			
Clinical checklist		X	X
Prioritising the checklist (or not)		X	
Previous experience of using checklists and identifying benefits		X	
Checklists are a useful memory prompt		X	
Patient and carer education leaflet	X		

For peer review only

CFIR Domains and the LEGS bundle	Patients	Secondary care clinicians	GPs
Targets that are impossible to reach	X		
**4.2 Self-efficacy**			
The whole bundle		X	
Confidence to implement guidelines		X	
**4.3 Individual stage of change**			
Clinical checklist		X	
Put myself forward to find out more		X	
Patient and carer education leaflet	X	X	X
If I was younger and I had more get-up-and-go	X		
It's down to willpower at the end of the day (I like smoking, I like drinking, I like the telly) – patient engagement & stage of change – resistance from patients	X	X	X
The only thing left is something nice to eat	X		
Resistance from patients		X	
Secondary care doctors tell people to do things			X
Everybody knows they need to stop smoking			X
**4.4 Individual identification with organisation:** how individuals perceive the organization and their relationship and degree of commitment with that organisation.			
The whole bundle		X	
Joined up working with primary care		X	
Patient and carer education leaflet	X	X	X
The patient – health professional relationship	X	X	
It takes time to build the relationship and have the conversation		X	
Clear open communication	X		
Frank conversations for some	X	X	
Be honest and explain the risks		X	
Information on the benefits versus taking pleasure away		X	
Striking a balance to optimise people's quality of life. You can't take everything away.		X	
Patient preferences and shared decision making		X	
Online tools to support the clinical conversation		X	
Language and approach used to engage people			X
Consider use of motivational interviewing techniques in secondary care			X
GP action letter			X
Partnership working			X
Avoid the extra step and order the test in secondary care			X
**4.5 Other personal attributes:** other personal traits such as tolerance of ambiguity, intellectual ability, motivation, values, competence, capacity, and learning style.			
Clinical checklist		X	
Team members who prioritise PAD		X	
Patient and carer education leaflet	X		
Living with other health conditions alongside (arthritis, diabetes)	X		
Managing the pain	X		
Medication side-effects	X		

CFIR Domains and the LEGS bundle	Patients	Secondary care clinicians	GPs
**DOMAIN 5: PROCESS**			
**5.1 Engaging:** Attracting and involving appropriate individuals in the implementation and use of the intervention through a combined strategy of social marketing, education, role modelling, training, and other similar activities.			
The whole bundle		X	
Championing the cause		X	
Doing the bare minimum and going home on time		X	
Using research to demonstrate a difference		X	
Involving stakeholders at an early stage		X	
Clinical checklist		X	
Posters in clinic to remind staff		X	
**5.2 Executing:** Carrying out or accomplishing the implementation according to plan.			
Patient and carer education leaflet	X		
A direct contact number for the vascular team	X		
Primary care capacity	X		
My GP and practice nurses keep an eye on me	X		
Sometimes it can take weeks to get them to answer a query over the phone	X		
GP action letter	X		
It can take weeks to get information on their system	X		
It's lots of different clinics and different people involved in your healthcare (whole person care)	X		

CFIR matrix and distribution of descriptive themes

For peer review only

Full coding framework

Theme 1: The potential to make a difference

1.1 An opportunity to meet patient need

1.1.1 Improves care and outcomes
1.1.1.1 Standardises a minimum quality of care
1.1.2 Improves awareness and understanding of PAD
1.1.3 Improves awareness of existing NICE guidance
1.1.3.1 Provides a tool to support implementation of NICE guidance
1.1.3.1.1 The guidance is clear but we don't always follow it
1.1.4 Builds your relationship with the patient
1.1.4.1 Empowers patients by being informed

1.2 Shared primary and secondary care working

1.2.1 Working as a part of a whole team
1.2.1.1 Allows for secondary care oversight
1.2.1.2 An opportunity for collaborative working
1.2.1.2.1 Managing expectations and understanding each other's roles
1.2.1.2.2 We don't sing off the same hymn sheet
1.2.1.2.2.1 The patient is in the middle
1.2.1.3 Multidisciplinary working
1.2.1.3.1 Enabling specialist care from different people
1.2.1.4 Promoting continuity of care
1.2.1.4.1 There's lots of different clinics and different people involved in your care
1.2.1.4.2 Timely access back into secondary care if needed
1.2.2 Joining up and building relationships across the team
1.2.2.1 We don't ever see secondary care specialists
1.2.2.2 Access to the vascular team (or not)
1.2.2.2.1 A direct contact number for the vascular team
1.2.2.3 We've done our bit and now it's over to the GP
1.2.3 Clear open communication across community and hospital services
1.2.4 Working together and learning together
1.2.4.1 An opportunity for two-way learning
1.2.4.1.1 Secondary care can learn from this too
1.2.4.1.1.1 Support for screening questions on prescribing and contraindications
1.2.4.1.1.1.1 Holistic picture of medication profile, side effects, competing medication
1.2.4.1.1.2 Familiarity (or not) with reviewing cholesterol and HbA1c's – it's a GP thing

Theme 2: A solution for addressing the gap in no man's land

2.1 We need to target patients earlier

2.1.1 Patients presenting too late
2.1.1.1 Delayed referral to secondary care
2.1.1.1.1 We can't make a referral anyway
2.1.1.1.1.1 Secondary care referral thresholds are too high
2.1.1.1.1.1.1 Patients don't meet the criteria so have to wait until it gets worse
2.1.1.2 Waiting for secondary care to make the diagnosis
2.1.1.2.1 Support general practice to do some of the diagnostics
2.1.1.2.1.1 Lack of access to testing equipment in the community
2.1.1.2.1.2 Secondary care are gatekeepers for ABPIs and duplexes

2.2 Primary care needs to do more sooner

2.2.1 Adapt for use in general practice as part of a PAD prevention programme

For peer review only

2.2.1.1 Would promote earlier conversations with patients
2.2.1.2 Useful for managing patient expectations
2.2.1.3 Would support primary care to start best medical therapy
2.2.1.4 Could be used to provide additional information for referral to vascular team
2.2.1.4.1 Would support triage in secondary care
2.2.2 Use as an ongoing conversation guide
2.2.2.1 Existing relationships with patients will help to reinforce the message
2.2.2.2 Informing patients reduces the chance of presenting late or in crisis

Theme 3: Prioritising and making it happen

3.1 Prioritising alongside the burden of work
3.1.1 It shouldn't be seen as an extra thing
3.1.1.1 Previous experience of using intervention/checklists
3.1.1.2 Checklists are a quick quality check
3.1.1.3 Checklists are a useful memory prompt
3.1.1.4 Checklists are a threat to autonomy
3.1.1.5 Checklists are tick-box exercises rather than safety mechanisms
3.1.2 Confidence to implement guidelines
3.1.2.1 It's essentially what I always do
3.1.3 It takes time to build the relationship and have the conversation
3.1.3.1 Time to talk requires extra staff
3.1.3.2 Time burden of dictating long clinic letters
3.1.3.3 Limited access to blood patient monitoring and cholesterol tests in out-patient clinics
3.1.4 Impact of staff shortages
3.1.4.1 Reduced staffing due to covid
3.1.4.2 Reduced district nurse staffing for conducting ABPIs
3.1.5 Primary care is not an infinite resource
3.1.5.1 Long waiting times to be seen or to get tests
3.1.5.1.1 Sometimes it can take weeks to get them to answer a query over the phone
3.1.5.2 Sixty calls and forty letters a day
3.1.5.3 Patients don't always see the same GP
3.1.5.4 Championing the cause or doing the bare minimum and going home on time
3.1.5.4.1 Using research to demonstrate a difference

3.2 Working across primary and secondary care systems
3.2.1 Preventative medicine and health promotion is core to primary care practice
3.2.1.1 Existing systems in place for primary care medication management and monitoring
3.2.1.1.1 It can take weeks to get information on the system
3.2.1.1.2 Time delay between writing and receiving clinic letters
3.2.1.1.3 It can take up to three months to receive hospital letters
3.2.1.1.3.1 Provide 28 days medication to allow for delay in seeing GP
3.2.1.1.3.2 Patient difficulty ordering repeat medication
3.2.1.1.4 Avoid the extra step and order the test in secondary care
3.2.1.1.5 Secondary care to initiate smoking cessation referral
3.2.1.1.5.1 Local variation in community set-up of smoking cessation, dietary advice, exercise therapy support
3.2.1.1.5.1.1 No formal set-up in primary care to pick it up
3.2.1.1.5.1.2 Link with national protocol for weight reduction
3.2.1.1.6 It's not commissioned or financially viable
3.2.2 Provide a copy of the clinic/GP letter to the patient to reinforce shared messaging
3.2.2.1 Patients to hand deliver letters to general practice
3.2.3 Repeat GP action letter is a duplicate, adds to the workload, and sets the wrong tone
3.2.3.1 It gives the impression that secondary care do not trust GPs

3.3 Adapting the intervention to the environment

3.3.1 Fits the in-patient environment neatly
3.3.1.1 Incorporate into a universal admission/discharge bundle
3.3.1.1.1 Use at point of admission and discharge for in-patients
3.3.2 Use the intervention to support the outpatient clinic consultation
3.3.2.1 A record of the consultation conversation
3.3.2.2 A resource for referring back
3.3.3 Complexity in assessing patients remotely in the community

3.4 Training to support implementation

3.4.1 Teams are not static so require ongoing access to training
3.4.1.1 Prioritise junior staff – the people who do the day-to-day work
3.4.1.1.1 Consider including medical students
3.4.1.2 Written information for those unable to attend training
3.4.1.3 Webinar-ed out
3.4.2 Training delivered by vascular team to primary care
3.4.2.1 Involve all primary care staff and not just GPs
3.4.2.1.1 Highlight the link between PAD and cardiovascular death
3.4.2.1.2 Provide education on managing CLTI and non-CLTI patients in primary care
3.4.2.1.3 Taking an ABPI in primary care
3.4.2.1.4 Learning gaps in exercise and lifestyle modification
3.4.2.2 Visibility of vascular teams at primary care conferences
3.4.2.3 Use available supervision and de-briefing opportunities

Theme 4: Personalised information and supportive conversations for taking on the advice

4.1 Offer personalised care and tailor patient information to individual needs

4.1.1 Existing information does not suit all
4.1.1.1 Currently most likely to suit those with IC
4.1.1.2 Targets are impossible to reach
4.1.1.2.1 If I was younger and had more get-up-and-go
4.1.1.2.2 Availability of supervised exercise
4.1.1.2.3 Relying on others for food shopping
4.1.2 A different approach for each patient
4.1.3 Offer realistic targets or the advice rings hollow
4.1.4 Patient education leaflet is provided too late
4.1.4.1 Too far gone to talk about lifestyle modification
4.1.4.1.1 I can only do what I can do
4.1.4.2 Consider co-existing morbidities
4.1.4.3 Offer advice on moderating activity levels and pain management
4.1.4.4 Discuss concerns about medication side-effects

4.2 Everyone knows they need to stop smoking

4.2.1 Acknowledge and support patient preference and promote shared decision making
4.2.1.1 It's easy to bombard someone with information
4.2.1.2 Secondary care doctors tell people to do things
4.2.1.2.1 Consider the language and approach to engage patients
4.2.1.2.2 Be honest and explain the risks
4.2.1.2.2.1 Use available online tools to support the conversation
4.2.1.2.3 Use motivational interviewing techniques
4.2.1.2.4 Frank conversations for some
4.2.2 It's down to willpower at the end of the day
4.2.2.1 I like smoking, I like drinking, I like the telly

For peer review only

4.2.2.2 The only thing left is something nice to eat
4.2.2.2.1 Provide information on the benefits versus taking pleasure away
4.2.2.2.2 Strike a balance to optimise people's quality of life
4.2.2.2.2.1 You can't take everything away
4.2.3 Patient education leaflets are not the best way for everyone
4.2.3.1 I don't want any more leaflets
4.2.3.1.1 District nurses can answer my questions

For peer review only

COREQ checklist

Item number	Descriptor	Reported on page number
1	Interviewer/facilitator: Which author/s conducted the interview or focus group?	8
2	Credentials: What were the researcher's credentials? E.g. PhD, MD	8
3	Occupation: What was their occupation at the time of the study?	8
4	Gender: Was the researcher male or female?	
5	Experience and training: What experience or training did the researcher have?	8
6	Relationship established: Was a relationship established prior to study commencement?	N/A
7	Participant knowledge of the interviewer: What did the participants know about the researcher? E.g. personal goals, reasons for doing the research	N/A
8	Interviewer characteristics: What characteristics were reported about the interviewer/facilitator? E.g. bias, assumptions, reasons and interests in the research topic	8
9	Methodological orientation and theory: What methodological orientation was stated to underpin the study? E.g. grounded theory	6-7
10	Sampling: How were participants selected? E.g. purposive, theoretical	7
11	Method of approach: How were participants approached? E.g. face-to-face, telephone, email	7
12	Sample size: How many participants were in the study?	9
13	Non-participation: How many people refused to participate or dropped out? Reasons?	9-10
14	Setting of data collection: Where was the data collected? E.g. clinic, work place	8
15	Presence of non-participants: Was anyone else present besides the participants and researchers?	N/A
16	Description of sample: What are the important characteristics of the sample? E.g. demographic data, date	9
17	Interview guide: Were questions, prompts, guides provided by the authors? Was it pilot tested?	7-8, Data supplement
18	Repeat interviews: Were repeat interviews carried out? If yes, how many?	N/A
19	Audio/visual recording: Did the research use audio or visual recording to collect the data?	8
20	Field notes: Were field notes made during and/or after the interview or focus group?	8
21	Duration: What was the duration of the interviews or focus group?	8
22	Data saturation: Was data saturation discussed?	N/A
23	Transcripts returned: Were transcripts returned to participants for comment and/or correction?	N/A
24	Number of data coders: How many data coders coded the data?	8-9
25	Description of the coding tree: Did authors provide a description of the coding tree?	10-11, data supplement
26	Derivation of themes: Were themes identified in advance or derived from the data?	8
27	Software: What software was used to manage the data?	8
28	Participant checking: Did participants provide feedback on the findings?	N/A

For peer review only

29	Quotations presented: Were participant quotations presented to illustrate the themes/findings? Was each quotation identified? E.g. participant number	11-16
30	Data and findings consistent: Was there consistency between the data presented and the findings?	11-16 All findings illustrated with quotes
31	Clarity of major themes: Were major themes clearly presented in the findings?	11-16
32	Clarity of minor themes: Is there a description of diverse cases or discussion of minor themes?	11-16

Reference: Tong A, Sainsbury P, Craig J. Consolidated criteria for reporting qualitative research (COREQ): a 32-item checklist for interviews and focus groups. International Journal for Quality in Health Care. 2007; 19(6): 349-357.

For peer review only
